# Update of the Brazilian Guideline on Nuclear Cardiology - 2020

**DOI:** 10.36660/abc.20200087

**Published:** 2020-02

**Authors:** Luiz Eduardo Mastrocola, Barbara Juarez Amorim, João Vicente Vitola, Simone Cristina Soares Brandão, Gabriel Blacher Grossman, Ronaldo de Souza Leão Lima, Rafael Willain Lopes, William Azem Chalela, Lara Cristiane Terra Ferreira Carreira, José Roberto Nolasco de Araújo, Cláudio Tinoco Mesquita, José Claudio Meneghetti

**Affiliations:** 1 Hospital do Coração (HCor), São Paulo, SP - Brazil; 2 Universidade Estadual de Campinas (Unicamp), Campinas, SP - Brazil; 3 Sociedade Brasileira de Medicina Nuclear (SBMN), São Paulo, SP - Brazil; 4 Quanta Diagnóstico e Terapia, Curitiba, PR - Brazil; 5 Hospital das Clínicas da Universidade Federal de Pernambuco, Recife, PE - Brazil; 6 Hospital Moinhos de Vento, Porto Alegre, RS - Brazil; 7 Clínica Cardionuclear, Porto Alegre, RS - Brazil; 8 Universidade Federal do Rio de Janeiro (UFRJ), Rio de Janeiro, RJ - Brazil; 9 Fonte Imagem Medicina Diagnóstica, Rio de Janeiro, RJ - Brazil; 10 Clínica de Diagnóstico por Imagem (CDPI), Grupo DASA, Rio de Janeiro, RJ - Brazil; 11 Instituto do Coração (Incor) do Hospital das Clínicas da Faculdade de Medicina da Universidade de São Paulo (HCFMUSP), São Paulo, SP - Brazil; 12 Cardiologia Nuclear de Curitiba (CNC), Curitiba, PR - Brazil; 13 Diagnose - Centro de Diagnóstico por Imagem, Maceió, AL - Brazil; 14 Universidade federal Fluminense (UFF), Rio de Janeiro, RJ - Brazi

**Table:** 

Declaration of potential conflict of interest of authors/collaborators of Update of the Brazilian Guidelines on Nuclear Cardiology - 2020If the last three years the author/developer of the Update:							
Names Members of the Update	Participated in clinical studies and/or experimental trials supported by pharmaceutical or equipment related to the guideline in question	Has spoken at events or activities sponsored by industry related to the guideline in question	It was (is) advisory board member or director of a pharmaceutical or equipment	Committees participated in completion of research sponsored by industry	Personal or institutional aid received from industry	Produced scientific papers in journals sponsored by industry	It shares the industry
Barbara Juarez Amorim	No	No	No	No	No	No	No
Claudio Tinoco Mesquita	NIH	Bayer	No	No	Pfizer	No	No
Gabriel Blacher Grossman	No	No	No	No	No	No	No
João Vicente Vitola	No	No	No	No	No	No	No
José Claudio Meneghetti	No	No	No	No	No	No	No
José Roberto Nolasco de Araújo	No	No	No	No	No	No	No
Lara Cristiane Terra Ferreira Carreira	No	No	No	No	No	No	No
Luiz Eduardo Mastrocola	No	No	No	No	No	No	No
Rafael Willain Lopes	No	No	No	No	No	No	No
Ronaldo de Souza Leao Lima	No	No	No	No	No	No	No
Simone Cristina Soares Brandão	No	No	No	No	No	No	No
William Azem Chalela	No	No	No	No	No	No	No

**List of Abbreviations and Acronyms**
HED - ^11^C - meta-hydroxyephedrine labeled with Carbon-11
PIB-^11^C - PET - pittsburgh B compound labeled with carbon-11 by PET imaging
MIBG-^123^I - metaiodobenzylguanidine labeled with iodine 123
^13^NH^3^ - ammonia labeled with Nitrogen-13
H_2_O-^15^O - water labeled with Oxygen-15
FDG-^18^F - fluorodeoxyglucose labeled with Fluorine-18
FDG-^18^F - PET/TC - fluorodeoxyglucose labeled with fluorine-18 by hybrid imaging (positron emission tomography coupled with computerized tomography)
Sodium fluoride-^18^F - fluorine-18 labeled Sodium Fluoride for PET Amyloid Imaging
^201^Hg - mercury-201
^201^Tl - thallium-201
^82^Rb - rubidium-82
^82^Sr - strontium-82
^99m^Tc - technetium-99m
MIBI-^99m^Tc - technetium-99m-labeled SESTAMIBI or MIBI
Pyrophosphate-^99m^Tc - technetium-99m-labeled pyrophosphate
ACEI - angiotensin converting enzyme inhibitors
ACS - acute coronary syndrome
Aden - adenosine
ADMIRE-HF - AdreView Myocardial Imaging for Risk Evaluation in HF
AF - atrial fibrillation
AHA - American Heart Association
AL - light chain immunoglobulin
ALARA - as low as reasonably achievable
AMI - acute myocardial infarction
angio-CT - angiotomography of coronary arteries
ARB - angiotensin receptor blockers
ATP III - Adult Treatment Panel, from the Program for Detection, Evaluation, and Treatment of High Cholesterol in Adults
AUC - area under the curve
AVB - atrioventricular blockage
BMI - body mass index
BNP - B-natriuretic peptide
CA - cardiac amyloidosis
CABG - coronary artery bypass graft
CC - coronary calcium
CAD - coronary artery disease
CCA - coronary cineangiography
CFR - coronary flow reserve
CHF - congestive heart failure
CIED - cardiac implantable electronic devices
CMR - cardiac magnetic resonance
CONFIRM - Coronary CT Angiography Evaluation for Clinical Outcomes: an International Multicenter Registry
COURAGE - Clinical Outcomes Utilizing Revascularization and Aggressive druG Evaluation Trial
CPU - chest pain unit
CRP - C reactive protein
CRT - cardiac resynchronization therapy
CS - calcium score
CTX - cardiotoxicity
CV - cardiovascular
Cx - circumflex coronary artery
CZT - cadmium zinc telluride semiconductors
DDD - artificial pacemaker stimulation mode
DG1 - diagonal 1 coronary artery
Dipy. - dipyridamole
DM - diabetes mellitus
Dobut. - dobutamine
DS - duke score
ECG - 12-lead electrocardiogram
ECHO - echocardiogram
EDV - end diastolic volume
ERASE Chest Pain -The Emergency Room Assessment of Sestamibi for Evaluation of Chest Pain Trial
ESV - end systolic volume
ET - exercise testing
FAME - Fractional Flow Reserve versus Angiography for Guidance of PCI in Patients with Multivessel Coronary Artery Disease
FBP - filtered back-projection
FDA - food and drug administration
FDG-6-P - fluorodeoxyglucose - 6 - phosphate
FFA - free fatty acids
FFR - fractional flow reserve
FRS - Framingham risk score
Gated-SPECT - myocardial perfusion imaging by single photon emission computed tomography technique synchronized with electrocardiogram
HBP - high blood pressure
HF - heart failure
HFpEF - heart failure with preserved ejection fraction
HFrEF - heart failure with reduced ejection fraction
HMR - heart to mediastinum ratio
HR - heart rate
IAEA - International Atomic Energy Agency
ICD - implantable cardioverter defibrillator
ICNC - International Conference of Nuclear Cardiology
IE - infectious endocarditis
IFR - instantaneous flow reserve/instantaneous wave-free ratio
INCAPS - IAEA Nuclear Cardiology Protocols Cross-Sectional Study
ISCHEMIA - International Study of Comparative Health Effectiveness with Medical and Invasive Approaches
IV - intravenously/intravenous
keV - kilo-electron volts
LAD - left anterior descending coronary artery
LAFB - left anterior fascicular block
LBBB - left bundle branch block
LV - left ventricle
LVAD / VAD - left ventricular assist device / ventricular assist devices
LVEF - left ventricular ejection fraction
MBF - myocardial blood flow
MBFR - myocardial blood flow reserve
MBq - megabequerel
mCi - milicurie
MET - metabolic equivalent.
MFR - myocardial flow reserve
MIBI / SESTAMIBI - 2-methoxy-isobutyl-isonitrile
MPS - myocardial perfusion scintigraphy
MR - magnetic resonance
MRS - myocardial revascularization surgery
mSv - millisieverts
MVO_2_ - myocardial oxygen consumption
NaI - sodium iodine
NE - norepinephrine
NPV - negative predictive value
NSTEMI - non-ST segment elevation myocardial infarction
NSVT - nonsustained ventricular tachycardia
NYHA HF - New York Heart Association Heart Failure Class
OMT - optimized medical therapy
OR - odds ratio
OSEM - ordered subset expectation maximization
PAREPET - prediction of arrhythmic events with positron emission tomography
PARR-2 - PET and Recovery after Revascularization study
PCI - percutaneous coronary intervention
PET - positron emission tomography
PET/CT - positron emission tomography coupled with computed tomography (hybrid imaging)
PET/MR - positron emission tomography coupled with magnetic resonance (hybrid imaging)
PM - pacemaker
PREMIER - Performance of Rest Myocardial Perfusion Imaging in the Management of Acute Chest Pain in the Emergency Room in Developing Nations
PROCAM - PROSpective CArdiovascular Munster Study
PROMISE - Prospective Multicenter Imaging Study for Evaluation of Chest Pain
RCA - right coronary artery
Regad. - regadenoson
RESCUE - Randomized Evaluation of patients with Stable angina Comparing diagnostic Examinations
ROC - receiver operating characteristics
ROI - regions of interest
ROMICAT II - rule out myocardial infarction by cardiac computed tomography
RV - right ventricle/ventricular
SBC - Brazilian Society of Cardiology
SBMN - Brazilian Society of Nuclear Medicine
SBP - systolic blood pressure
SCORE - Systematic Coronary Risk Evaluation Study
SDS - summed difference score
Shining / Shine Through - residual activity effect
SPECT - myocardial perfusion imaging by single photon emission computed tomography
SRS - summed rest/redistribution score
SSS - summed stress score
STEMI - ST segment elevation myocardial infarction
STICH - Surgical Treatment for Ischemic Heart Failure study
SUS - Brazil’s public Single Health System (acronym in Portuguese)
SUV - standard uptake value
TIA - transient ischemic attack
TID - transient ischemic dilatation
TOF - time of flight
TTR - transthyretin
TTR CA - transthyretin cardiac amyloidosis
UA - unstable angina
USA - United States of America
VAD - ventricular assist devices
VF - ventricular fibrillation
VT - ventricular tachycardia
WR - myocardial washout rate

## 1. Introduction

Nuclear cardiology is a non-anatomical, physiological imaging method. The use of radioactive or radiopharmaceutical substances makes it possible to study several physiopathological mechanisms of cardiovascular disease *in vivo*. Via this imaging technique, it is also possible to visualize and accompany an instituted therapy’s physiological effects on cardiac function, on the cellular and biochemical level. Of all the applications of nuclear medicine in cardiology, scintigraphy or myocardial perfusion imaging with technetium-99m-labeled radiopharmaceuticals synchronized with electrocardiogram (Gated-SPECT), is the most common exam in clinical practice. For this reason, this technique will be the most discussed in these Guidelines.

Recent years have, however, seen a growing concern among the scientific community regarding rational and optimized use of ionizing radiation in medicine. Cardiovascular imaging, moreover, encompasses all functional and anatomical imaging techniques and should, in this context, be used rationally and cost-effectively. Other applications of nuclear medicine in cardiology have also emerged and gained prominence during the past decades, especially positron emission tomography (PET) for the study of coronary flow reserve, cardiac sympathetic activity, and inflammatory/infectious processes, and cardiac amyloidosis (CA). All of these aspects have been taken into consideration and will be covered in detail in the chapters developed herein.

Guidelines recommendations are highly valuable tools for medical activity of the highest quality. The objective is to support and aid doctors in making decisions regarding their patients, by elaborating orientations which may be useful as part of the decision-making process. No Guidelines, however, should be replaced by the abilities, experience, and clinical judgments of specialized professionals who are have the final say in their decisions concerning each individual patient.

In general, whenever possible and applicable, classifications of recommendation have been adopted for indicating cardiac scintigraphy, supported by levels of evidence, in accordance with the recommendations established by classical cardiology guidelines (Table 1).

**Table 1:** Classes of recommendation and levels of evidence

Classes of recommendation
Class I - Conditions for which there is conclusive evidence or, in the absence of conclusive evidence, general consensus that the procedure is safe and useful/effective
Class II - Conditions for which there are conflicting evidence and/or divergent opinions regarding the procedure's safety and usefulness/effectiveness
Class IIA - Weight or evidence/opinion in favor of the procedure. The majority of studies/experts approve.
Class IIB - Safety and usefulness/effectiveness less well established, with no prevailing opinions in favor
Class III - Conditions for which there is evidence and/or consensus that the procedure is not useful/effective and could, in some cases, be harmful
**Levels of Evidence**
Level A - Data obtained from multiple concordant large randomized trials and/or robust meta-analysis of randomized clinical trials
Level B - Data obtained from less robust meta-analysis, from a single randomized trial, or from non-randomized (observational) trials
Level C - Data obtained through consensus of expert opinion

Based on current evidence, this document, which does not function as a substitute, practically and objectively adds important data to and updates the Brazilian Cardiology Society’s (SBC) **First Guidelines** and **Update** on **Nuclear Cardiology**, both of which were published by the Brazilian Archives of Cardiology (Arquivos Brasileiros de Cardiologia), in 2002 and 2005, respectively.

As in the previously mentioned documents, those who participated in the elaboration of these Guidelines are considered specialists in their respective areas and were, for this reason, chosen to develop the chapters thereon. The committed involvement of all colleagues representing the SBC and the Brazilian Society of Nuclear Medicine (SBMN) have made the elaboration of these **new update of Brazilian Guidelines on Nuclear Cardiology** possible. It is our hope that they will be of great use, especially to Cardiologists and Nuclear Medicine and Clinical Physicians in Brazil. The Organizing Committee appreciates the collaboration of all those involved.

## 2. Addendum to the ISCHEMIA Study*

At the time of publication of this guideline, the International Study of Comparative Health Effectiveness with Medical and Invasive Approaches (ISCHEMIA) had not been published yet, although the main findings were presented on November 16, 2019 at the American Heart Association (AHA) annual congress in Philadelphia, USA, available on the study’s website. Considering its importance for medical decision-making and the potential implications for nuclear cardiology, a few relevant concerns should be highlighted on the findings available so far:

1. The main objective of the ISCHEMIA study was to assess whether patients (P) with at least moderate ischemia on a functional examination would benefit from myocardial revascularization (coronary artery bypass grafting or percutaneous coronary intervention) added to optimal medical therapy). Were randomized 5,179 patients with stable CAD and myocardial ischemia documented by one of many different methods (myocardial perfusion scintigraphy, stress echocardiography, cardiac magnetic resonance imaging, exercise testing not associated with cardiac imaging). These noninvasive methods were used to define the etiology of chest pain and for cardiovascular risk stratification, a management approach established in clinical practice that is not invalidated by the findings of the study. Prior knowledge indicates that patients with lower ischemic burden have a better prognosis than individuals with larger and more intense ischemia;
2. The ISCHEMIA trial demonstrated no benefit of myocardial revascularization (Invasive Group - IG) versus optimal medical therapy (OMT) to reduce the major outcomes of “death” and “acute myocardial infarction.” Despite the methodological differences, these results were somewhat similar to those of the COURAGE study. It is noteworthy that the mortality curves began to separate after two years of medical follow-up, apparently benefiting the IG and potential long-term implications, which justified the increased clinical follow-up of P, underway at the moment. Note that the IG had an improved quality of life assessment, reduced frequency of angina and lower use of specific medication compared to the OMT group;
3. The ISCHEMIA trial is one of the most relevant studies on stable CAD, with important messages for clinical practice. The validity of the results is emphasized for the population sample evaluated in the study and for the definitions of ischemia and its severity levels employed. However, for exclusion situations, such as P with left main disease, recent acute coronary syndrome, angioplasty in the previous 12 months, ejection fraction < 35% and progressive or unstable symptoms, prior knowledge remains unchanged. Both CAD and ischemic heart disease represent a broad spectrum of patients, with inherent heterogeneity and important prognostic implications (extensive evidence base in the literature and described in detail in the current guideline). Were excluded from the trial an impressive number of P that had at least moderate angina and ischemia in the absence of coronary obstructions, showing the diversity of the disease and the value of functional assessment;
4. The main question is whether the ISCHEMIA study has properly evaluated a significant number of P with moderate/severe ischemia, aiming to determine whether myocardial revascularization adds prognostic benefit to these patients, as documented by scintigraphy, which was not the exclusive method of documentation. There was the inclusion (randomization) of cases with nonexistent or mild ischemia (12% of the total randomized), which is surprising for a study that was initially intended to include only patients with moderate to severe ischemia. There was also a change in the criteria for inclusion of P with severe ischemia in the study, with a significant number based on the results of exercise testing, without imaging, a decision made after the study was in progress. From this change, the percentage of these P that would effectively have severe myocardial ischemia on scintigraphy is questioned;
5. Therefore, the Editorial Board of this guideline believes that the definitive analysis of the results will only be possible after the formal publication of the trial results.

## 3. The Application of Nuclear Medicine Techniques to Justify Financial Resources Available for Attending Cardiology Patients in Brazil

### 3.1. Introduction

Cardiovascular diseases are the main cause of death in Brazil, and they are responsible for 30% of deaths worldwide every year.^1^ They are responsible for approximately 8% of total healthcare costs in Brazil, a figure which has been increasingly annually, in parallel with population aging.^1^ Teich and Araújo estimated that in 2011, approximately 200,000 events associated with acute coronary syndromes occurred in Brazil, entailing a massive impact of 3.88 billion Brazilian reals, considering only hospital and indirect costs, associated with loss of productivity.^2^ Considering these findings, it has been demonstrated that (preventive) measures play a crucial role in reducing morbidity and mortality, and they should be a priority in national healthcare policy design, as they have profound additional impacts on reducing costs and maintaining productivity. Another significant point, however, which has contributed to reducing the outcome of “cardiovascular death” and to justifying expenses, involves the use of tools which make accurate diagnosis of a determined condition possible (?) and which aid and guide the conduct of physicians, based on these results. Myocardial perfusion scintigraphy (MPS) plays a significant role in justifying financial resources for attending patients with established or potential cardiovascular disease.

### 3.2. Cost-Effectiveness in Comparison with Cardiac Catheterization

One of the main fundaments of MPS is its good ability to identify low-risk patients, who do not require invasive intervention, in spite of established coronary disease, such as anatomical lesions on coronary angiography.^3^ Observational studies in the 1990’s have demonstrated that MPS was able to identify high- and low-risk groups, resulting in reduced costs for patients with coronary artery disease (CAD) and avoiding procedures that are not associated with improved patient health outcomes. A major prospective study carried out in the United States of America (USA) recruited 11,372 patients with stable angina, who were referred to either MPS or cardiac catheterization. Patients were adjusted by clinical risk, and the costs of direct cardiac catheterization (aggressive strategy) were compared to initial scintigraphy followed by selective catheterization in high-risk patients (conservative strategy). Although both strategies had similar adverse outcomes, such as cardiac death and non-fatal myocardial infarction, revascularization rates were higher (between 13% and 50%) in patients who underwent catheterization directly.^4^ This reflex of revascularizing anatomical lesions which do not determine ischemia led to unnecessary associated medical costs of around 5,000 dollars per patient in this study.^4^ Currently, the use of medical resources for conditions that do not have consequences for patients or that could be managed conservatively is known as “overtreatment.”^5^ The study of the impact of MPS on reducing costs has shown that its main function is to prevent patients who have low or moderate risks on single photon emission computed tomography (SPECT) from being treated with unnecessary catheterizations and revascularizations. Similarly to this North American study, Underwood et al.^6^ have demonstrated that strategies which incorporate myocardial scintigraphy to evaluate patients with stable coronary diseases are both cheaper than and as effective as strategies involving invasive anatomical assessment.^6^ Cerci et al.^7^ evaluated the impact of diagnostic exams on patients with CAD in different scenarios within Brazil’s public Single Health System (SUS, acronym in Portuguese). The study’s most relevant finding is that, although non-invasive functional tests are the most frequently solicited exams for evaluating patients with suspected or known CAD, the majority of healthcare costs for these patients are related to procedures/invasive treatment. In other words, in the Brazilian context, the costs of diagnostic exams continue to be significantly lower than those of invasive and therapeutic procedures. In this manner, it seems logical to affirm that, if scintigraphy exams are made available to patients attended by the SUS, there will be a similar impact on the reduction of healthcare costs, which has been the case in the USA and some countries in Europe. Another relevant piece of data from this study refers to the fact that the majority of patients who were revascularized had not undergone tests to document ischemic burden; only anatomical diagnostic techniques had been applied.^7^

### 3.3. Cost-Effectiveness of Myocardial Perfusion Scintigraphy in Relation to Coronary Angiotomography

Angiotomography (angio-CT) of coronary arteries offers very accurate, non-invasive anatomical assessment, and it has proved to be an excellent technique for ruling out obstructive coronary disease in low- to intermediate-risk patients. Angio-CT, however, has presented results similar to those of cardiac catheterization in relation to triggering a higher number of myocardial revascularizations, which do not necessarily (means) reduced cardiovascular outcomes. In a recent meta-analysis comparing angio-CT to functional methods, no differences were observed regarding the outcomes of death or cardiac hospitalization, but there was a 29% reduction in the number of non-fatal infarctions. On the other hand, the use of this method was associated with 33% and 86% higher rates of invasive coronary angiography and myocardial revascularization, respectively. It is not known whether the reduction in non-fatal infarctions may be attributed to the higher number of revascularizations, which is (unlikely) considering in light of other studies on stable CAD, or to the higher use of statins and aspirin associated with the recognition of anatomical coronary lesions.^8^ With the objective of elucidating the role of angio-CT on cost-effectiveness of approaches to stable CAD in comparison with myocardial scintigraphy, the Randomized Evaluation of Patients With Stable Angina Comparing Diagnostic Examinations (RESCUE) study, which is being developed, is expected to compare these strategies in a prospective, randomized manner.^9^

The authors of a recent meta-analysis published by the American Heart Association (AHA)/Circulation, have reinforced 2 important aspects of cost-effectiveness:^10^

- The importance of performing appropriate exams as a way of (ensuring) their cost-effectiveness, especially techniques like MPS.
- The results of appropriate exams should effectively lead to appropriate decision making in clinical conduct and patient management.

## 4. Indications for Myocardial Perfusion Scintigraphy

Over the past years, different medical societies have published criteria for defining scenarios in which myocardial scintigraphy may be adequately utilized. In addition to traditional classification of recommendation and levels of evidence, more recent criteria on appropriate MPS exam referral have been suggested, dividing indications into appropriate, possibly appropriate, and rarely appropriate, resulting from the application of scores constructed based on clinical scenarios and specific methodologies.^11^ In this classification, indications with scores from 1 to 3 are considering rarely appropriate; 4 to 6, possibly appropriate; and 7 to 9, appropriate. Published documents are based on evidence from American and European Guidelines, as well as the recently published Brazilian Guidelines on stable coronary disease.^12-15^

Regardless of classification type, there is consensus that symptomatic patients with intermediate risks of ischemic heart disease are the ones who most benefit from MPS in terms of diagnostic and prognostic evaluation. The exam should preferably be performed in association with physical exercise in patients with sufficient physical and clinical conditions (estimated ability for activities of daily living with metabolic expenditure greater than 5 METs), in order to measure their functional capacity, hemodynamic responses (heart rate and blood pressure behavior), stress-induced arrhythmias, and other responses. It is recommended that patients with complete left bundle branch block, regardless of functional ability, undergo MPS under pharmacological stress (dipyridamole or adenosine). In the same manner, regardless of pretest probability of ischemic heart disease, patients with low functional ability or uninterpretable electrocardiogram (ECG) are indicated to undergo MPS. On the other hand, patients with low probability of ischemic heart disease, higher functional ability, and interpretable ECG are not indicated for MPS (Table 2).

**Table 2:** Indication criteria for myocardial perfusion scintigraphy in symptomatic patients

Assessment of patients with non-acute chest pain or ischemic equivalent	Score
Low pretest probability of CAD, with interpretable resting ECG and ability to exercise	3
Low pretest probability of CAD, with uninterpretable resting ECG or inability to exercise	7
Intermediate pretest probability of CAD, with interpretable resting ECG and ability to exercise	7
Intermediate pretest probability of CAD, with uninterpretable resting ECG or inability to exercise	9
High pretest probability of CAD, regardless of interpretable resting ECG and ability to exercise	8

ACS: acute coronary syndrome; CAD: coronary artery disease; ECG: 12-lead electrocardiogram.

In patients with heart failure (HF) and left ventricular systolic dysfunction or recent-onset atrial fibrillation (AF), ventricular tachycardia (VT) or syncope, the indication for MPS is appropriate or possibly appropriate, unless the patient in question is low risk or has low pretest probability. Asymptomatic patients with no history of ischemic heart disease and without abnormal exercise testing (ET) generally do not benefit from undergoing MPS. In specific situations, in patients with high calcium scores (greater than or equal to 400), diabetes, chronic renal insufficiency, or a prevalent family history of ischemic heart disease, performing MPS may aggregate value to the medical decision-making process, with satisfactory cost-effectiveness. Asymptomatic patients with abnormal stress ECG who are re-stratified with the use of prognostic scores, such as the Duke score, may also benefit from complementary investigation via MPS, especially if their risk scores are intermediate or high (Table 3). Diverse examples of clinical situations cited in Table 3 may also be found in the *section on integration of diagnostic modalities*.

**Table 3:** Indication criteria for myocardial perfusion scintigraphy in asymptomatic patients and/or patients with prior exams

Asymptomatic patients - detection of CAD/risk stratification	Score
Low risk (ATP III criteria)	1
Intermediate risk (ATP III criteria) - interpretable ECG	3
Intermediate risk (ATP III criteria) - uninterpretable ECG	5
High risk (ATP III criteria)	7
High risk and calcium score (Agatston) between 100 and 400	7
Calcium score (Agatston) > 400	7
Low-risk Duke score (> +5)	2
Intermediate-risk Duke score (between -11 and + 5)	7
High-risk Duke score (< -11)	8

Agatston: score that defines the presence and quantity of calcium in coronary arteries, characterizing atherosclerosis; ATP III: Adult Treatment Panel, from the program for detection, evaluation, and treatment of high cholesterol in adults; CAD: coronary artery disease.

When patients have established ischemic heart disease and are asymptomatic, early myocardial perfusion studies with radiopharmaceuticals should be avoided following percutaneous coronary intervention and/or myocardial revascularization surgery procedures. In the event of percutaneous coronary intervention and myocardial revascularization surgery, the application of MPS has been observed to have a favorable cost-benefit ratio for follow up after more than 2 and 5 years, respectively, even in asymptomatic patients. Symptomatic patients with specific clinical conditions (or equivalent manifestations) may benefit from the exam before this period (Table 4).

**Table 4:** Indication criteria for myocardial perfusion scintigraphy in patients who have undergone revascularization procedures (CABG or PCI)

Previous percutaneous revascularization or surgical procedures	Score
Symptomatic	8
Asymptomatic, CABG less than 5 years prior	5
Asymptomatic, CABG 5 or more years prior	7
Asymptomatic, percutaneous revascularization less than 2 years prior	3
Asymptomatic, percutaneous revascularization 2 or more years prior	6

CABG: myocardial revascularization surgery; PCI percutaneous coronary intervention.

For patients with previous exams who manifest new symptoms or who require assessment of the repercussion of diagnosed intermediate lesions and characterization of arteries with obstructive lesions “responsible” for a larger myocardial area at risk, as well as patients with multivascular diseases, the indication for MPS is classified as appropriate or possibly appropriate. In patients with established coronary disease and worsening symptoms, MPS may aid in quantifying ischemic burden (extent and intensity of defects) and in determining medical management. In clinically stable patients with previous exams performed more than 2 years prior, MPS may be appropriate (Table 5).

**Table 5:** Indication criteria for myocardial perfusion scintigraphy for risk stratification and prognostic assessment of patients with proven stable coronary artery disease and/or prior exams

Asymptomatic patients or patients with stable symptoms - previously "normal" stress imaging exams	Score
Intermediate/high risk (ATP III) - stress imaging exam ≥ 2 years prior	6
**Asymptomatic patients or patients with stable symptoms - CCA or abnormal imaging exams, without prior CABG**	
CAD on CCA or "abnormal" stress imaging exam (exam performed > 2 years prior)	5
CAD on CCA or "abnormal" stress imaging exam (exam performed < 2 years prior)	3
Previously "unclear," "contradictory," or "borderline" non-invasive assessment - obstructive CAD as initial concern	8
**New, recent, or progressive symptoms**	
Abnormal CCA or abnormal stress imaging exam	9
Normal CCA or normal stress imaging exam	6
**Coronary cineangiography (invasive or non-invasive)**	
Coronary stenosis or anatomical abnormality whose significance is unclear	9

ACS: acute coronary syndrome; ATP III: Adult Treatment Panel, from the program for detection, evaluation, and treatment of high cholesterol in adults; CAD: coronary artery disease; CCA: coronary cineangiography; CABG: myocardial revascularization surgery.

In patients who present acute chest pain, with clinical suspicion of acute coronary syndrome (ACS), normal or uninterpretable ECG (old left bundle branch block or pacemaker) and normal biomarkers, resting myocardial scintigraphy may exclude acute cardiovascular events with a high degree of safety (high negative predictive value [NPV]), allowing patients to be discharged from the emergency room. If the exam is normal, investigation may continue with outpatient tests involving physical or pharmacological stress, whether associated or non-associated with non-invasive imaging, and even anatomical assessment via coronary angio-CT, in specific conditions. For patients with ACS who are clinically stable, with neither recurring chest pain nor HF, and who have not undergone any invasive exam, MPS is useful for detecting presence and extent of myocardial ischemia (Table 6).

**Table 6:** Indication criteria for myocardial perfusion scintigraphy in patients with acute chest pain or post-acute coronary syndrome

Assessment of patients with acute chest pain	Score
Resting image only	
Possible ACS - ECG without ischemic alterations or LBBB or pacemaker; low-risk TIMI score; borderline, minimally elevated, or negative troponin	8
Possible ACS - ECG without ischemic alterations or LBBB or pacemaker; high-risk TIMI score; borderline, minimally elevated, or negative troponin	7 / 8
Possible ACS - ECG without ischemic alterations or LBBB or pacemaker; negative initial troponin. Recent (up to 2 hours) or evolving chest pain	7
**Assessment of post-ACS patients (infarction with or without elevated ST segment)**	
Stable, post-AMI patients, with ST segment elevation, for assessment of ischemia; cardiac catheterization not performed	8
Stable, post-AMI patients, without ST segment elevation, for assessment of ischemia; cardiac catheterization not performed	9

ACS: acute coronary syndrome; AMI: acute myocardial infarction; CAD: coronary artery disease; ECG: 12-lead electrocardiogram; LBBB: left bundle branch block.

Indications for MPS to assess pre-operative risk of non-cardiac surgeries and vascular surgeries have also been recently revised.^16^ Patients who will undergo low-risk surgeries do not need to undergo MPS. If the surgery is not low-risk, functional capacity is the factor that determines whether MPS will be necessary. In patients with functional capacity estimated at greater than or equal to 4 METs, without cardiac symptoms, regardless of clinical or surgical risk, non-invasive assessment of myocardial ischemia is generally not recommended. However, for patients with low functional capacity and elevated clinical/surgical risks, there is an indication to perform MPS under pharmacological stress. The following are considered clinical risks: history of ischemic heart disease, congestive heart failure (CHF), cerebrovascular disease, diabetes mellitus (DM), and renal insufficiency (creatinine > 2.0 mg/dl). In the absence of these risk factors, regardless of functional capacity, surgery may be performed without complementary functional exams (Table 7).

**Table 7:** Indication criteria for myocardial perfusion scintigraphy for pre-operative assessment of non-cardiac surgeries

Pre-operative assessment of non-cardiac surgeries	Score
Low-risk surgery	1
Intermediate-risk surgery or vascular surgery Functional capacity greater than or equal to 4 METs	1
Intermediate-risk surgery or vascular surgery Functional capacity unknown or less than 4 METs No clinical risk factors	1
Intermediate-risk surgery Functional capacity unknown or less than 4 METs One or more clinical risk factors	7
Vascular surgery Functional capacity unknown or less than 4 METs One or more clinical risk factors	8

MET: metabolic equivalent.

In patients with accentuated left ventricular dysfunction who are eligible for myocardial revascularization, assessment of myocardial viability may aid selection of patients who will benefit from this treatment (Table 8).

**Table 8:** Indication criteria for myocardial perfusion scintigraphy for assessment of myocardial viability

Assessment of myocardial viability	Score
Accentuated left ventricular dysfunction	9
Eligible for myocardial revascularization	9

MPS is, therefore, an appropriate indication in diverse clinical manifestations of ischemic heart disease, from acute manifestations in the emergency room to diagnostic investigation of stable patients, aiding in therapeutic decision making through various tools which make it possible to define disease severity, as well as in pre-operative assessment in specific situations and in defining the benefits of revascularization for patients with significant myocardial viability. It is worth noting that, for diagnostic investigation, patients with intermediate probability of ischemic heart disease are those who most benefit from MPS and that it is rarely appropriate in patients with low probability.

## 5. Myocardial Perfusion Scintigraphy Methods - types of Cardiovascular Stress

### 5.1. Radiopharmaceuticals Used to Perform Myocardial Perfusion Scintigraphy

In Brazil, the main radiopharmaceuticals available for obtaining images of the myocardium are thallium-201 (^201^Tl) and those labeled with technetium-99m (^99m^Tc), which mainly include 2-methoxy-isobutyl-isonitrile, known as Sestamibi (or MIBI), and tetrofosmin. Given that these are the most widely used, the specific methods used for acquiring images with them will be presented.

**Thallium-201 or ^201^Tl** ^17^ is a monovalent cation with biological properties analogous to those of potassium. It is both intracellular and absent in scar tissue, and it is thus designated for differentiating ischemic myocardium from fibrosis. It has a physical half-life of 73 hours, and it decays by electron capture to mercury-201 (^201^Hg), and the photons emitted for imaging are primarily x-rays (of ^201^Hg itself) between 68 and 80 kilo-electron volts (keV), in addition to lower quantities of gamma radiation in the energy range of 135 keV and 166 KeV. Upon intravenous injection, initial myocardial uptake is proportional to regional blood flow, depending on the integrity of the cellular membrane. It penetrates the cellular membrane via active transport, involving energy expenditure (Na^+^/K^+^ATPase system), with a high first-pass extraction fraction in the myocardium (the proportion of ^201^Tl which is extracted from blood and absorbed by myocytes), of around 70% to 85%.

Maximum concentration of thallium-201 in the myocardium occurs approximately 5 minutes after injection, which is generally administered during peak exercise or clinical and/or electrocardiographic alterations triggered during an ET or a pharmacological test. It presents rapid disappearance or clearance from the intravascular compartment. Following initial distribution of the radioisotope throughout the myocardium, related to blood flow, the phenomenon of redistribution begins 10 to 15 minutes after injection. This is dependent on clearance or washout of thallium-201 from the myocardium, which no longer depends on blood flow but rather on the concentration gradient between myocytes and blood levels. Redistribution of thallium-201 is quicker in normal myocardium than in ischemic myocardium, resulting in different activities in these tissues (differential “washout”).

**Figure 1:**
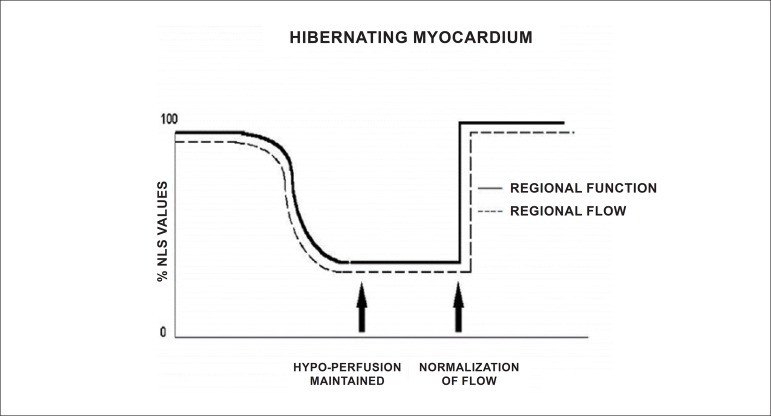
Hibernation represented as persistent decrease of blood flow and contractile function. Recovery of function is immediate following restoration of coronary flow. %: percent values; NLS: normals.

Source: Adapted from Dilszian.^277^

Due to the characteristics described and the ability to evaluate the integrity of the cellular membrane, thallium-201 has the additional property of studying myocardial viability, predominantly related to hibernating myocardium (Figure 1).^18-20^ This represents the condition of resting left ventricular dysfunction, resulting from chronic hypoperfusion in myocardial regions where, although the myocytes have remained viable (alive), they have chronically depressed contractile function. Hibernation may also be seen as a “flow-contraction” agreement process, where metabolism remains dependent on residual myocardial flow in a manner sufficient for minimum substrate supply and inhibitory substance removal. Therefore, the condition of hibernation, notwithstanding reduced resting coronary flow, is not necessarily associated with the presence of chronic ischemia, given that oxygen supply and consumption ratio may be preserved.^21,22^

**Technetium-99m-labeled SESTAMIBI or MIBI (MIBI- ^99m^Tc):**^23,24^ The most frequently used marker for myocardial perfusion studies, is 2-methoxy-isobutyl-isonitrile, a stable, lipophilic, cationic compound belonging to the isonitrile family, which has the property of crossing cellular (sarcolemmal) membranes and binding to myocyte mitochondria through the mechanism of passive diffusion, depending on the electrochemical transmembrane gradient. It therefore involves no energy expenditure. It has a lower first-pass extraction fraction in the myocardium than thallium-201, of approximately 60%.^25^

**Figure 2:**
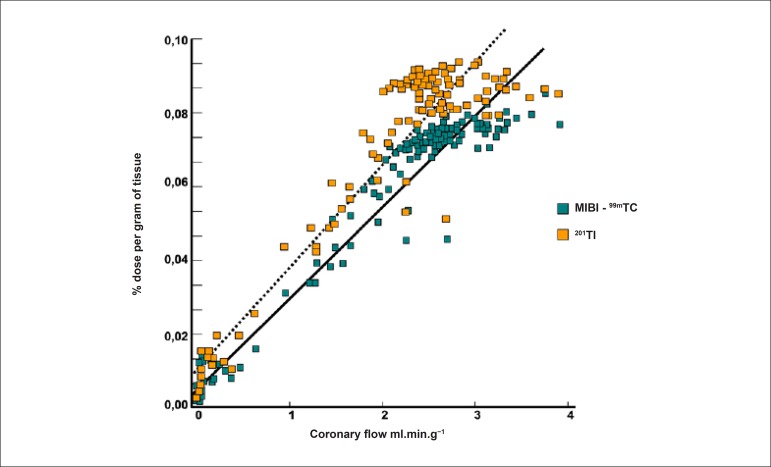
Linear association between intravenous dose per gram of myocardium and blood flow per minute, using the radiopharmaceuticals ^201^Tl and MIBI-^99m^Tc. Once coronary flow exceeds 2.5 ml.min.g^-1^, a loss of linear relationship is observed (phenomenon of “roll off”).

Source: Adapted from Berman DS.^116^

It does not expressively present the phenomenon of redistribution, largely remaining retained within mitochondria. This property makes it necessary to deliver 2 separate injections of the radiopharmaceutical, 1 during the resting and 1 during the stress phase. This may be done either on the same day or on different days. As MIBI is not radioactive, it must be labeled with technetium-99m (^99m^Tc), which has a physical half-life of 6 hours and emits gamma photons in the energy range of 140 keV (photopeak). Similarly to thallium-201, initial myocardial uptake is proportional to regional blood flow, depending on the integrity of the cellular membrane. In this manner, a linear relationship is observed between the intravenous dose per gram of myocardium and blood flow per minute (Figure 2), starting at minimal flow ranges of approximately 2.0 to 2.5 milliliters per gram.minute^-1^, values normally found in maximum exercise testings. When very high coronary flow are reached, generally over 3.0 mililiters per gram.minute ^-1^, the linear relationship between this variable and myocardial uptake is lost, with decreased blood extraction of the radiopharmaceutical, in a phenomenon known as “roll off”.^26-28^ Nonetheless, owing to higher energy emission (higher photopeak), measured in keV, it presents higher quality images, in comparison with thallium-201. Finally, the elimination of MIBI-^99m^Tc takes place through the hepatobiliary system, whereas elimination of thallium-201 is mainly achieved through the renal system. Regarding other isonitriles approved by the FDA for assessment of obstructive CAD, only tetrofosmin, whose properties are similar to those of MIBI-^99m^Tc, has been made available for clinical use.

### 5.2. Myocardial Perfusion Scintigraphy with Tomography Imaging (SPECT)

Technological evolution of computerized systems has made it possible to divide the myocardium of the left ventricle (LV) into tomographic slices measuring only a few millimeters. In conventional gamma cameras (with iodide sodium crystals) the size of a pixel (the smallest component of a digital image) is 6.4 mm, and in CZT (cadmium zinc telluride semiconductors) technology it is 4 mm, representing related cross sections and, consequently, the method’s spatial resolution.^29-31^ The resulting images facilitate the separation of nearby regions, improving contrast resolution and allowing for better detection of differences in concentrations of radioactivity in the myocardium. The SPECT technique also allows for detection of ischemic regions, even those that are small in size, i.e., approximately 2% of LV mass, in tissue with relatively normal tracer concentration.

**Figure 3:**
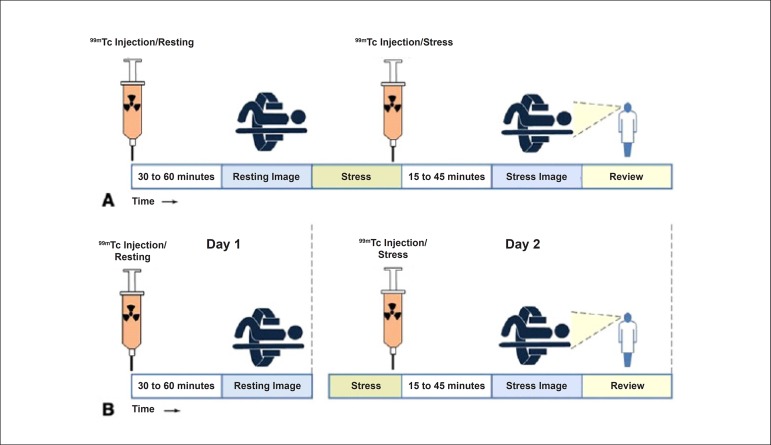
Perfusion image acquisition and myocardial function with the radiopharmaceuticals sestamibi (MIBI) or tetrofosmin labeled with technetium-99m or ^99m^Tc: “one-day” protocol (A) and “two-day” protocol (B). The legends “^99m^Tc Injection/Resting” and “^99m^Tc Injection/Stress” represent administration of the radiopharmaceutical MIBI-^99m^Tc during both stages, with dosage measured in millicuries (mCi), established in accordance with equipment and acquisition model used, as well as patient weight. In Protocol A, the stress dose is 3 times higher than the resting dose; in Protocol B, the resting and stress doses are similar, considering an interval of 24 hours between image acquisition.

**Protocols:** The preferred means of obtaining perfusion images of the myocardium and LV function with tracers labeled with technetium-99m (^99m^Tc) is known as the “1-day protocol” (Figure 3A), made up of 2 stages, (resting-stress or stress-resting). During the first step, the injected dose of MIBI-^99m^Tc, measured in millicuries (mCi) or megabecquerels (mBq), is three times lower than the dose administered during the second phase, thus avoiding the residual activity effect or “shining through” phenomenon. Another option is the “2-day protocol” (Figure 3B), where in each phase is performed on a separate day. In this case, similar doses and acquisition parameters are used. It is important to emphasize that, in situations where stress images are taken before resting ones, even if the perfusion images are normal, it is nevertheless important to obtain resting images, except in specific cases, given that analysis of LV function in both situations may provide relevant information, including the possibility of detecting patients with homogenous tracer distribution due to balanced severe coronary diseases. Furthermore, the detection of transient LV dilatation may also be useful in this case, and this requires that both phases be performed. However, in asymptomatic patients who have intermediate/low risks and no clinical evidence of CAD, who have undergone the stress phase as the initial MPS stage and whose perfusion images are normal, it is possible to dispense with the resting phase, in what is known as the “stress only protocol.” In this situation, recent studies have provided evidence that the test’s prognostic value is maintained and that diagnostic ability is similar to the costs of high sensitivity. Furthermore, the patient receives a lower dose of radioactive activity, and total exam time is reduced.^32,33^

### 5.3. Myocardial Perfusion Scintigraphy with Tomographic Images Synchronized with Electrocardiogram (Gated-SPECT)^34-41^

Cardiac images should be acquired synchronized with patient ECG, allowing for additional analysis of ventricular function, simultaneous with myocardial perfusion evaluation. This information adds data to the medical decision-making process within known incremental prognostic values, and it improves test accuracy, especially regarding specificity values. Considering this aspect, in situations where there are doubts between persistent perfusion defects and/or artifacts (due to breast or diaphragmatic attenuation), analysis of ventricular wall motility and thickness may contribute to differentiating these two causes. When apparent reduced relative uptake of a radiopharmaceutical is due to an artifact, the motility and systolic thickness of this wall are normal.

The estimated results of left ventricular ejection fraction (LVEF) that are conventionally considered normal vary according to technique and methodology employed. With the Gated-SPECT technique, this value is ≥ 50% for both sexes; there are few references with differentiated values for men and women, in addition to different established limits of normality. Due to specific aspects related to methodologies used to calculate LVEF, values found in individuals who are shorter and individuals with smaller ventricular cavities and/or hypertrophic ventricles, especially in women, may be overestimated, at times exceeding values of 75% to 80%.

Calculations of LVEF and ventricular volumes obtained by Gated-SPECT may be utilized for prognostic stratification. LVEF < 45% and end systolic volume (ESV) > 70 ml are associated with increased risks of cardiac death.^42,43^ This analysis may be carried out either while resting or under stress; it should preferably be done during both steps, however, considering the possibility of detecting transient LV dysfunctions induced by physical exercise or pharmacological stress.

Cardiac arrhythmias pose difficulties to the acquisition of ECG-synchronized images and may significantly influence the results obtained for ejection fraction and produce artifacts in myocardial perfusion images. There is a technically defined time window for RR interval variation, generally around 20%, after which point heartbeats are rejected. This situation means that if there is an arrhythmia which produces variations between RR intervals above these established limits, such as persistent AF, the corresponding data from that specific cardiac cycle will be rejected, and there will consequently be lower counting statistics. In these cases, images should be acquired without ECG synchronization in order to avoid the occurrence of artifacts.

### 5.4. Cardiovascular Stress

The basic principle of using cardiovascular stress associated with myocardial perfusion images consists of creating heterogeneity in blood flow between vascular territories irrigated by normal coronary arteries with significant obstructive stenoses.^44,45^ The use of myocardial perfusion agents makes it possible to visualize this heterogeneity in regional blood flow. In practice, of all existing cardiovascular stressors, only ET and pharmacological tests have been used.

Both stress modalities, physical exercise and pharmacological vasodilation, have similar sensitivity and specificity for the detection of CAD via analysis of perfusion images.^46-48^

**Physical stress:** ET is the associated method of choice for diagnostic and prognostic values, which have already been established in conformity with clinical, hemodynamic, and electrocardiographic variables obtained during exercise, which add incremental data to myocardial perfusion study. Stress tests have a higher chance of revealing abnormalities in patients with more severe and extensive obstructive arterial disease. Chest pain and/or decreased systolic blood pressure (SBP) during low levels of exercise are highly important findings that are associated with adverse prognoses and multivessel coronary disease. Other markers of unfavorable prognosis include high-magnitude ST segment depression, with a horizontal or downsloping aspect, which may appear early during low workloads or be characterized by late recovery after stress has ceased, present in multiple leads, among others (Table 9).

**Table 9:** Exercise testing parameters associated with unfavorable prognosis and multivessel coronary disease.

• ECG:
- ST-segment depression ≥ 2 mm, with descending morphology and early appearance (metabolic load < 5 - 6 METs), involving multiple leads, usually lasting for ≥ 5 minutes of recovery
- Exercise-induced ST-segment elevations
- Reproducible, symptomatic, or sustained ventricular tachycardia (> 30 s)
• Metabolic load < 5 - 6 METs*
• Chronotropic incompetence
• Systolic blood pressure: inability to reach values ≥ 120 mmHg, or sustained decrease ≥ 10 mmHg, or fall below resting values during progressive exercise
• Symptoms: angina pectoris when performing a lower workload , generally during the beginning of exercise, when conventional protocols are applied

ECG: electrocardiogram; MET: metabolic equivalent. (*1 MET = oxygen consumption in supine resting conditions, equivalent to 3.5 mL.kg^-1^.min^-1^)

Some studies have incorporated stress test variables into diagnostic and prognostic scores.^49^ The most widely used in our context is the Duke prognostic score. Using Cox’s regression analysis, Mark DB et al. proposed^50^ and validated^51^ this score for use with the exercise treadmill test and the Bruce protocol. It is calculated by the following formula:

$$\mathrm{DS}=T\left(\min\right)-\left(5\mathrm{xST}\right)-\left(4\mathrm{xAI}\right)$$

or

$$\begin{array}{c} \mathrm{Duke}\;\mathrm{Score}\;=\;\mathrm{exercise}\;\mathrm{time}\left(\mathrm{in}\;\min\mathrm{utes}\right)-\left(5\times \mathrm{ST}\;\mathrm{deviation}\right.\\ \left.\mathrm{in}\;\mathrm{mil}\lim\mathrm{eters}\right)-\left(4\times \mathrm{angina}\;\mathrm{index}\right) \end{array}$$

The angina index has a value of 0 (zero) if there are no symptoms during exercise, 1 (one) if non-limiting chest pain occurs, and 2 (two) if the pain is impeditive (growing intensity) as exercise proceeds. In accordance with the results of the regression equation, patients are classified as follows:

- **High-risk group**: patients with scores ≤ -11, with an annual cardiovascular mortality rate ≥ 5%.
- **Low-risk group**: patients with scores ≥ 5, with an annual cardiovascular mortality rate < 1%. In clinical practice, when patients are considered high-risk, this reinforces *a priori* the indication for invasive study with the aim of managing and directing medical treatment, be it interventional or not, while always taking the possibility of improving morbimortality and quality of life into account. In patients with intermediates results, i.e., scores between > -11 and < +5, in order to reclassify risk, complementary exams associated with imaging, such as the following, may be required:
  - Myocardial perfusion scintigraphy (MPS) with ET or vasodilators.
  - Vasodilator stress cardiac magnetic resonance (technique associated with inability to exercise).
  - Doppler echocardiogram under stress or specific conditions.
  - Computerized angiotomography of coronary arteries.

Finally, in patients considered low-risk, medical management is related to prevention measures. On the other hand, based on a growing base of evidence, these methods,^52^ especially MPS, have become of paramount importance for quantifying ischemic area, even in patients who are considered high-risk, with the aim of assisting and directing the medical approach to be adopted,^53-58^ notwithstanding the unavailability of information from randomized clinical trials such as the “Ischemia Study,” which will be able to assist in better management of patients with extensive areas of the myocardium at risk.^59^

Furthermore, emphasis given to exercise as the primary stress-producing agent of choice within the cardiovascular system has become clear, given that it is the most physiological method for triggering myocardial ischemia, based on sympathetic stimulation and the increase in the main determinants of myocardial oxygen consumption (MVO_2_), such as HR, blood pressure, and myocardial contractility. Likewise, exercise leads to coronary vasodilation through biochemical mechanisms, resulting in increased blood flow to the myocardium and greater oxygen supply, thus meeting the necessary demands imposed during the application of extreme effort. This ability to increase coronary blood flow, which reaches three to four times baseline values during peak exercise, in the absence of significant obstructive coronary lesions, conceptually represents the phenomenon known as “coronary reserve,” considered the main characteristic of MPS with radiopharmaceuticals. Moreover, with respect to the limitations and contraindications of this methodology,^60^ joint analysis of both stress test and cardiac imaging exams will play a fundamental role in the medical decision-making process, albeit in view of previous clinical information or pretest probability of obstructive CAD.

With relation to the main methodological aspects, the following stand out:

- Prior venous access in an arm, in a “Y” shape (separate routes), for radiopharmaceutical injection during peak exercise and subsequent flush with saline solution, respectively.
- Safety criteria for administering and interrupting stress should be in accordance with established guidelines, reinforcing the need for a maximum test.^61^
- Following intravenous administration of the radiopharmaceutical, stimulate continuation of stress for 1 more minute.
- When using MIBI-^99m^Tc (absolute preference in Brazil), image acquisition follows conventional protocols (30 to 60 minutes after stopping stress). Variations in initial acquisition time depend on patient type (obesity, prior abdominal surgery, prominent extracardiac activity in the resting images phase.
- When using thallium-201, considering the phenomenon of redistribution, images should be taken 10 to 15 minutes after stopping stress.

**Pharmacological tests:** Represent excellent alternatives for evaluating patients with physical limitations or clinical impediments to undergoing efficacious exercise testing . The most frequent conditions are found in Table 10. They represent around 20% to 30% of all cases of scintigraphy referral and approximately 50% of elderly patients.^62^ The drugs used in these circumstances are dipyridamole, adenosine or regadenoson, and dobutamine. These drugs induce maximum vasodilation and increase coronary flow, allowing for assessment of coronary reserve, with diagnostic and prognostic power similar to that of exercise,^63,64^ which has recently been extended to elderly patients and women.^65,66^

**Table 10:** Main indications for use of pharmacological stress in patients with contraindications or limitations to undergoing exercise stress^24,46^

• Motor sequelae from cerebral vascular insufficiency and degenerative or inflammatory musculoskeletal pathologies
• Compensated congestive heart failure
• Chronic pulmonary obstructive disease with important functional restriction, but without recent hyperresponsiveness
• Low functional capacity
• Other non-cardiac conditions that result in an inability to exercise efficiently
• Severe arterial hypertension
• Complex ventricular arrhythmias triggered by effort
• Pre-operative cardiological assessment for major abdominal vascular surgery
• Presence of left bundle branch intraventricular conduction disorders
• Risk stratification for recent evolution of myocardial infarction
• Use of drugs that interfere with oxygen consumption elevation
• Presence of artificial electric stimulation

In cases of left His bundle branch block or artificial pacemaker with ventricular stimulation, the first option is a pharmacological test with dipyridamole or adenosine, with the aim of avoiding what are known as false-positive results (alterations in relative radiopharmaceutical uptake, in the absence of obstructive lesions). These are caused by atypical movement of the interventricular septum, which occurs in these situations and is accentuated when myocardial scintigraphy is performed with ET. Reduced radiopharmaceutical uptake is often observed in these patients and is most frequently related to the septal region, which may be exacerbated by the stress test, as increased HR increases paradoxical septal motion and, consequently, reduces perfusion in this wall.^67,68^

**Primary vasodilators:** Dipyridamole, adenosine, and regadenoson (not available for routine clinical practice in Brazil) provoke a significant increase in coronary flow in normal arteries and a small or nonexistent increase in arteries with functionally significant stenosis, thus resulting in relative heterogeneity of flow between LV walls. During maximum vasodilatation, when the radioisotope is injected, the difference in relative radiopharmaceutical uptake in LV walls will also be observed, making it possible to diagnose coronary disease:

- **Dipyridamole:** the total dose of dipyridamole is 0.56 mg.kg^-1^ up to a maximum dose of 60 mg or 6 vials (a 2-ml vial = 10 mg), administered intravenously (IV), preferably with a 4-minute infusion pump, diluted in 50 ml of saline solution (SS). It may, alternatively, be injected manually (with a 20-ml syringe), using the same dilution. Alternatively, a more elevated dose of 0.84 mg.kg^-1^ may be used in select cases. The radiopharmaceutical is administered IV during hyperemia or maximum vasodilation, 2 to 4 minutes after the end of dipyridamole infusion (Figure 4). Dipyridamole inhibits the action of the enzyme adenosine deaminase, wich degrades endogenous adenosine, in addition to blocking reuptake of adenosine into the cellular membrane, with a consequent increase in extracellular concentration and resulting coronary vasodilation. Its biological half-life is approximately 45 minutes.
  **Figure 4:**
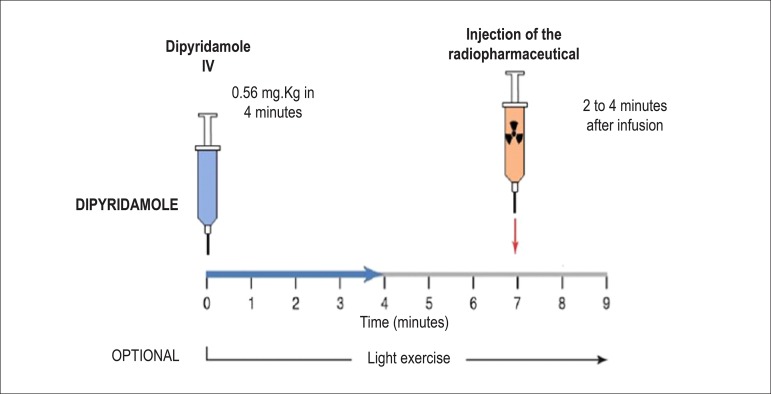
Myocardial perfusion scintigraphy associated with injection of dipyridamole. The moment of maximum vasodilation or coronary hyperemia occurs between 2 and 4 minutes after completing intravenous dipyridamole administration (blue arrow, 4 minutes), at which point the radiopharmaceutical (99mTc-tetrofosmin or MIBI-^99m^Tc, orange arrow) is injected. Clinical observation should be continuous throughout the exam, registering blood pressure, heart rate, and electrocardiogram every 2 minutes or in accordance with medical decision, with a typical total exam time of 9 to 10 minutes.^24,46^
- **Adenosine:** The usual dose is 140 µg.kg^-1^.min^-1^, and it must mandatorily be administered via a 6-minute continuous infusion pump, diluted in 50 ml of SS, with the injection of the radiopharmaceutical administered during the third minute via a different intravenous access (Figure 5). It is, also, possible to inject the solution for 4 minutes, in which case the radiopharmaceutical is administered during the second minute.^69^ Because xanthines block the vasodilation effect, patients should be instructed to suspend them for 24 hours before a scheduled exam with dipyridamole or 12 hours before a scheduled exam with adenosine, in addition to any other drug or product, food, or drink that contains methylxanthines or theophyllines, including coffee, tea, soft drinks, chocolate, energy drinks, compound analgesics containing caffeine, especially for treatment of muscular pain or migraines, et al. Reference lists are available for consultation.^70^ Adenosine induces coronary vasodilation via specific activation of A_2A_ receptors in the cellular membrane, resulting in increased coronary flow up to 4- or 5-fold resting values.
  **Figure 5:**
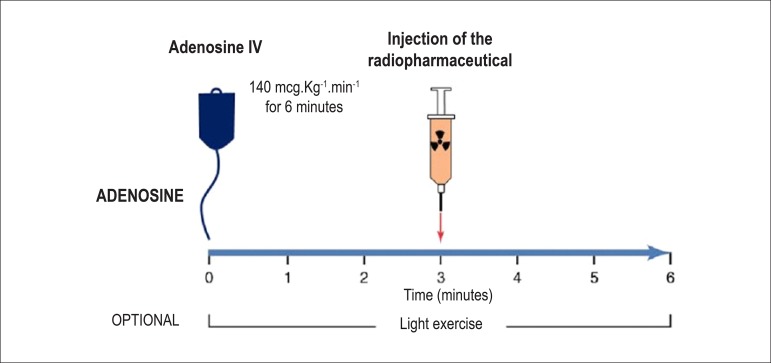
Myocardial perfusion scintigraphy associated with injection of adenosine. The need for continuous intravenous administration is due to the drug’s ultrashort plasma half-life (2 to 10 seconds), with the aim of maintaining coronary hyperemia, which reaches its peak close to the third minute. At this moment, the radiopharmaceutical (MIBI-^99m^Tc) is injected. After completing the solution at 6 minutes, frequent monitoring of blood pressure, heart rate, and electrocardiographic registers is maintained for a variable time of 4 to 6 minutes

Accuracy for detecting CAD with the use of MPS is comparable between both drugs. It is worth reiterating that, in exams using dipyridamole and adenosine, modifications in the ST segment occur relatively infrequently, even in patients with obstructive CAD (lower sensibility). In some instances, only the relative difference in flow observed in patients with different degrees of luminal obstruction and coronary reserve will determine perfusion defects, and ischemia will not necessarily be present. For this condition, collateral circulation is necessary, which causes coronary steal, with consequent alterations in contractility. Nevertheless, the sensitivity of scintigraphy images associated with the use of pharmacological agents or stress tests is similar. Adverse effects or “paraeffects” of using these drugs^23,71^ occur in approximately 50% of patients with dipyridamole and in up to 80% of patients with adenosine. Common side effects include headache, dizziness, flushed face, feeling hot, chest pain, ST alterations and others (Tables 11 and 12).^72^ These manifestations generally do not last long, and in most cases they may be reversed by administering intravenous aminophylline at 1 to 2 mg.kg^-1^ or 72 mg (3 ml) to 240 mg (10 ml or 1 vial) 2 minutes after injecting the radiotracer, when MPS is associated with dipyridamole. When adenosine is used, there is no need to inject an antagonist, given its ultrashort half-life, from 2 - 10 seconds, the recommendation being simply to interrupt the infusion. When it is not medically possible to perform either the physical stress or the pharmacological dilation modality with dipyridamole or adenosine, intravenous administration of dobutamine solution may be the best option for assessing coronary reserve flow, with regards to increased MVO_2_. Contraindications to dipyridamole and adenosine use are listed in Table 13.

**Table 11:** Adverse effects or "paraeffects" related to intravenous administration of dipyridamole for performance of myocardial perfusion scintigraphy^24,46^

Adverse effects or paraeffects	%
Chest pain	20
Headache	12
Dizziness	12
Alterations in ST	8
Ventricular extrasystoles	5
Nausea	5
Arterial hypotension	5
Facial flushing	3
Atrioventricular blockage	2
Fatal or non-fatal myocardial infarction	Extremely rare
Any minor event	50

**Table 12:** Adverse effects or "paraeffects" related to intravenous adenosine administration via infusion pump for performance of myocardial perfusion scintigraphy^24,46^

Adverse effects or paraeffects	%
Facial flushing	35 to 40
Chest pain	25 to 30
Shortness of breath	20
Dizziness	7
Nausea	5
Symptoms of hypotension	5
Atrioventricular blockage	8
Alterations in ST	5 - 7
Atrial fibrillation	Case reports
Convulsions	Case reports
Hemorrhagic/ischemic stroke	Case reports
Any minor event	80

**Table 13:** Contraindications to use of adenosine and dipyridamole^24,46^

**Absolute**
• Bronchospastic disease during activity, recent hyperreactivity (< 3 months), status asthmaticus
• Second- or third-degree atrioventricular blockage, in the absence of a pacemaker
• Arterial hypotension (systolic blood pressure less than 90 mmHg)
• Recent transient ischemic attack or cerebrovascular accident (< 2 months)
• Recent use (less than 24 hours) of dipyridamole in patients who are to receive adenosine
**Relative**
• History of reactive pulmonary disease, with no recent crises (> 3 months)
• Sinus node disease
• Severe sinus bradycardia
• Severe bilateral carotid disease

It is, finally, important to stress that, with both dipyridamole and adenosine, no significant increases are observed in MVO_2_, which, in clinical practice, is translated as the product of heart rate (HR) × systolic blood pressure (SBP), or the double product. During pharmacological stimulation, SBP values generally drop by around 10% while HR increases by approximately the same proportion, with no consequent increase in MVO_2_.

**Figure 6:**
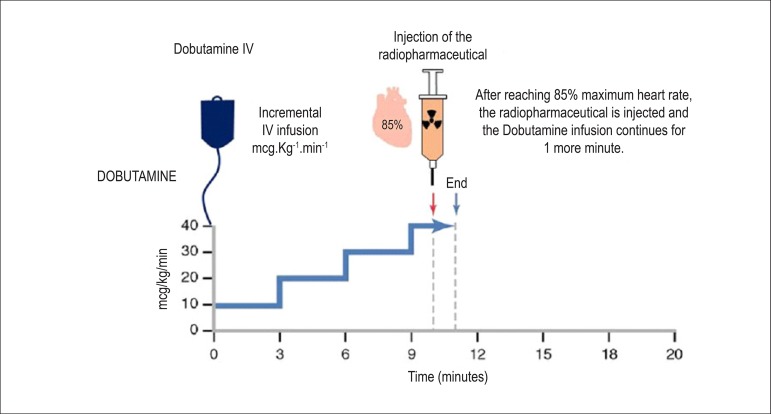
Myocardial perfusion scintigraphy associated with intravenous administration of dobutamine solution (250 mg or 1 vial diluted in 250 ml of saline solution). It may begin with an alternative initial dose of 5 mcg.kg^-1^.min^-1^, for 3 minutes, with sequentially increasing doses every 3 minutes, up to 40 mcg.kg^-1^.min^-1^ or until 85% of maximum heart rate has been reached (explained in the figure and the text), at which point the radiopharmaceutical (MIBI-^99m^Tc or ^99m^Tc-tetrofosmin) is injected. In the event of inadequate increase in heart rate and in the absence of contraindications (glaucoma, prostatic hypertrophy), atropine is additionally recommended, either early on or starting at the third step.

**Drugs that promote elevated myocardial oxygen consumption:** These drugs represent an alternative for patients who cannot undergo ET or pharmacological stress with *dipyridamole* or *adenosine*. Examples include patients who have contraindications or limitations for stress test, as well as pulmonary obstructive disease with recent crises of bronchial hyperreactivity, arterial hypotension (SBP < 90 mmHg), and significant obstructive carotid artery lesions on both sides. This is also an alternative modality in patients indicated for dipyridamole or adenosine who have ingested substances derived from caffeine or methylxanthines (competitive antagonists) over the past 24 and 12 hours, respectively. The most commonly used is *dobutamine*, which acts on beta-1 (β-1) adrenergic receptors, with chronotropic and inotropic stimulation, depending on the infused dose, in addition to direct effects on beta-2 (β-2) receptors, with peripheral vasodilation response. This results in an increase in cardiac output, HR, and SBP, leading to an increase in MVO_2_ and, consequently, in coronary vasodilation. **Protocol:** The protocol begins with venous administration of the solution (250 mg of dobutamine diluted in 250 ml of saline solution - 1 mg per 1 ml) via infusion pump at a dose of 10 ug.kg^-1^.min^-1^ for 3 minutes (first step), followed by 20 µg.kg^-1^.min^-1^ for 3 minutes (second step), adding 10 µg.kg^-1^.min^-1^ every 3 minutes (third and fourth steps) until the maximum dose of 40 µg.kg^-1^.min^-1^ has been reached (Figure 6).^73,74^ In patients who have not reached submaximal HR and who do not have evidence of ischemia, it is possible to associate intravenous atropine (0.25 to 2 mg) and perform isometric stress with hand grip maneuvers (e.g., compressing a tennis ball). A Brazilian study has demonstrated that early use of atropine (following the first phase of dobutamine infusion) is safe and that it reduces infusion time and complaints during stress, without affecting diagnostic precision.^75^ Furthermore, the presence of perfusion defects induced by pharmacological vasodilatation and motility abnormalities triggered by stress aggregate incremental prognostic value to the test, which has recently been validated with the use of ultrarapid cameras (CZT technology).^76^ Contraindications to dobutamine use may be found in Table 14. Patients on betablockers should stop taking these medications for 48 to 72 hours before the test. Special attention should be given to patients with bronchospasm undergoing MPS with dobutamine, whose plasma half-life is around 2 to 3 minutes, considering that its antagonist is metoprolol at an intravenous dose of 5 mg and that it is contraindicated in the presence of pulmonary obstructive disease. The most frequent adverse events or paraeffects associated with administration of dobutamine solution are listed in Table 15. To reverse them, in addition to metoprolol, other intravenous short-acting betablockers, such as esmolol (0.5 mg.kg), which is available, should be injected after the first minute of radiotracer injection.

**Table 14:** Contraindications to dobutamine use^24,46^

**Absolute**
• Cardiac arrhythmias including atrial fibrillation and ventricular tachycardia (sustained or non-sustained)
• Severe aortic stenosis and hypertrophic obstructive cardiomyopathy
• Systolic arterial hypotension (< 90 mmHg), uncontrolled systolic arterial hypertension (systolic > 200 mmHg), severe or stage III hypertension
• Unstable angina or recent myocardial infarction
• Aneurysms or aortic dissection
• Symptomatic vascular cerebral insufficiency
• Presence of implanted cardiac defibrillator
• Alterations in metabolism of potassium
**Relative**
• Abdominal aortic aneurysm (> 5 cm in diameter)
• Presence of thrombi in left ventricle
• Left ventricular ejection fraction < 25% (due to increased risk of ventricular arrhythmias)

**Table 15:** Adverse effects related to dobutamine infusion for myocardial perfusion scintigraphy^24,46^

Adverse effects	%
ST alterations	33
Precordial pain	31
Palpitation	29
Headache	14
Facial flushing	14
Dyspnea	14
Significant arrhythmias (supraventricular and ventricular)	8 to 10

**Combined stress:** The association of dynamic stress with low workloads (e.g., until the *second stage of the Bruce protocol* or *until feeling light fatigue*, equivalent to the number 13 on the subjective Borg stress scale) and vasodilators has been shown to reduce subdiaphragmatic (hepatic) activity and improve the ratio of radiation activity emitted between the target organ and the viscera (background), with consequent improvements in image quality.^77^ It has similarly shown a decrease in the occurrence of adverse effects resulting from the infusion of dipyridamole or adenosine, as well as the incidence of atrioventricular blockage. This protocol is ideal for patients who are able to exercise but who are using medications that limit increases in HR (betablockers, antiarrhythmic drugs, et al.).

**Figure 7:**
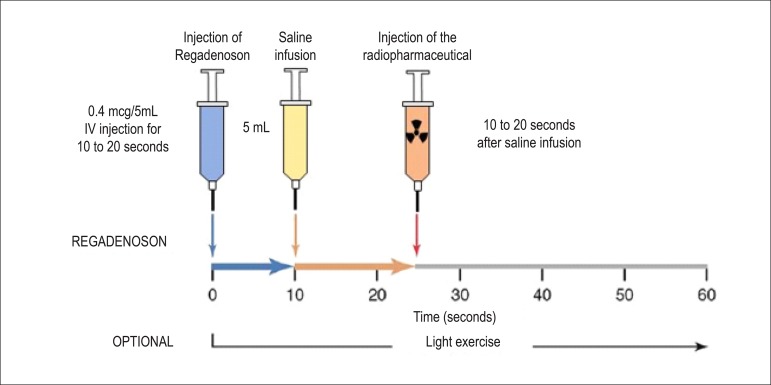
Myocardial perfusion scintigraphy associated with intravenous administration of regadenoson, a specific agonist of adenosine A_2_A receptors in the cellular membrane. Activation of the receptor produces coronary vasodilation with a consequent increase in flow, similar to dipyridamole and adenosine. Maximum plasma concentration is reached 1 to 4 minutes after injection, with a biological half-life of 2 to 4 minutes during the first phase. The intermediate and the late phases follow, with approximate duration of 30 minutes (loss of pharmocodynamic effect) and 2 hours (decline in plasma concentration). The radiopharmaceutical, MIBI-^99m^Tc or Tetrofosmin- ^99m^Tc, is injected at the moment of maximum hyperemia, close to 30 seconds after injection of regadenoson.

**New drugs:** There are 3 types of adenosine receptors (Table 16). The use of specific selective antagonists to A_2_ receptors has shown evidence of adequate coronary hyperemia and lower intensity of systemic effects, especially chest pain and atrioventricular blockage. A double-blind, randomized (regadenoson or adenosine), multicenter study^78^ involving 784 patients has shown that diagnostic information is similar and that there were no serious adverse effects; regadenoson, however, was tolerated better than adenosine. Second-degree atrioventricular blockage occurred in 3 patients with adenosine and in no patients with regadenoson. Regadenoson’s short biological half-life minimizes and limits the duration of adverse effects, diminishing monitoring time. It is administered via bolus, and it is not necessary to adjust dose to body weight (Figure 7). Its use is promising in patients with chronic obstructive pulmonary disease. The incidence of serious complications^79^ with the performance of cardiovascular stress is related in Table 17.

**Table 16:** Types of existing receptors in the cellular membrane and responses to stimuli

Type	Resulting effects
A1	Atrioventricular blockage
A2a	Coronary artery vasodilation
A2b	Peripheral vasodilation, bronchospasm
A3	Bronchospasm

**Table 17:** Serious adverse events related to cardiovascular stress methods (rate of events observed per 1,000 individuals)^79^

Serious events	ET	Dobut	Dipy	Aden	Regad
Any event	0.1 - 3.46	2.988	0.714 - 2.6	0.97	CR
Death	0 to 0.25	CR	0.5	CR	CR
VF/VT	0 to 25.7	0.6 - 1.35	NR	NR	NR
AMI	0.038	0.3 - 3	1	0.108	CR
Cardiac rupture	Unk	CR	NR	NR	NR
High-grade AVB / ASY	Unk	NR	CR	CR	CR
Bronchospasm	Unk	NR	1.5	0.76	CR
Stroke/TIA	Unk	CR	NR	NR	CR
AF	Unk	5 - 40	NR	NR	CR
Seizure	Unk	CR	NR	1.5	CR

Aden: adenosine; AVB: atrioventricular blockage; AF: atrial fibrillation; AMI: acute myocardial infarction; ASY: asystole; CR: case report; Dipy: dipyridamole; Dobut: dobutamine; ET: exercise testing; NR: not reported; Regad: regadenoson; TIA: transient ischemic attack; Unk: Unknown; VF/VT: ventricular fibrillation/ventricular tachycardia.

### 5.5. Image Generation and Perfusion Defects in Myocardial Scintigraphy with Radioisotopes

**Figure 8:**
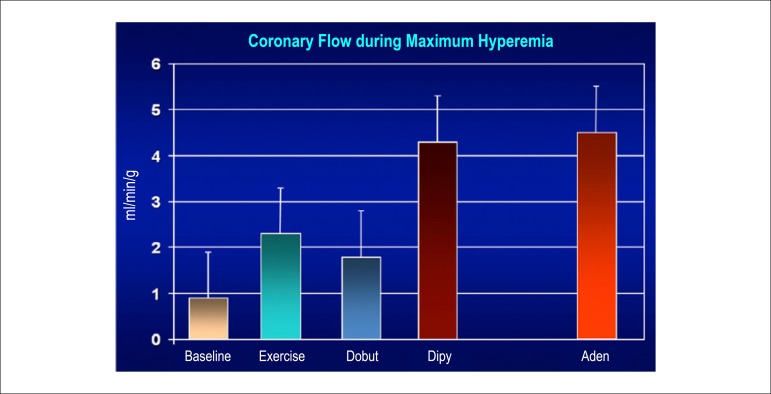
Effects of different types of stress methods on coronary flow elevation and values reached during maximum hyperemia. Baseline: resting coronary flow, 1 ml.min.g-1; Exercise: reaching values 2.5 to 3.5 times baseline coronary flow value; Dobut (dobutamine): reaching values around 2.0 to 2.5 times baseline coronary flow value; Dipy (dipyridamole) and Aden (adenosine): reaching values as high as 5.0 times baseline coronary flow value.^28^

Resting coronary flow is 1 ml.g.min^-1^, increased 3- to 5-fold during maximal vasodilation or hyperemia, under physical or pharmacological stress (Figure 8).^28^ In the presence of obstructive coronary lesions, resting coronary flow decreases when luminal narrowing is greater than 80%, due to exhaustion of the coronary reserve. When physical or pharmacological stress are applied, early exhaustion of the coronary reserve is observed, and it then exhibits a drop, generally beginning with lesions with luminal narrowing of 50%.^80^ This information has currently been validated based on invasive measures of coronary flow reserve (CFR), fractional flow reserve (FFR), and instantaneous flow reserve (IFR), considered “standard” for characterizing myocardial ischemia; some have also been reproduced by non-invasive PET methods.^81-86^ Tests with pharmacological stimulation using dipyridamole or adenosine associated with MPS are considered frequently to result in coronary flows in the range of 4 ml per gram of myocardium per minute,^87-89^ generating homogenous relative uptake patterns of the radioisotopes in the myocardium, and scintigraphy images are considered normal when the coronary arteries are free of atherosclerotic processes. There are, however, specific situations in which patients with balanced multivessel disease (lesions in 3 arteries with similar coronary reserve) in which perfusion images appear with apparently homogeneous radiopharmaceutical distribution.^90^

From the conceptual point of view, it is necessary to comprehend that the generation of scintigraphy images is based on relative radiopharmaceutical uptake, which is injected intravenously during physical exercise or pharmacological test, predominantly in the LV myocardium. Comparison of radiopharmaceutical uptake between ventricular walls is expressed in images based on a scale of colors, created by specific computer programs, which, in addition to allowing for subjective analysis of perfusion, make semi-quantitative and quantitative evaluation of affected myocardial area possible.

### 5.6. Possible Scintigraphy Imaging Results, Using Qualitative, Semi-quantitative, and Quantitative Analyses

**Figure 9:**
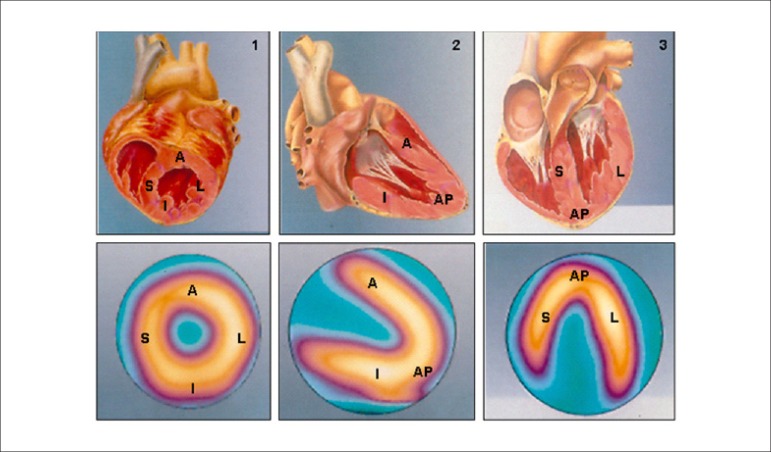
Two-dimensional reconstruction of scintigraphy images representing normal perfusion patterns (lower images), in line with minor axis (1), vertical long axis (2), and horizontal long axis (3) cross sections and their respective corresponding anatomical cross sections (upper images). A: anterior; AP: apical; I: inferior; L: lateral; S: septal. Adapted from Mastrocola LE.^195^

**Figure 10:**
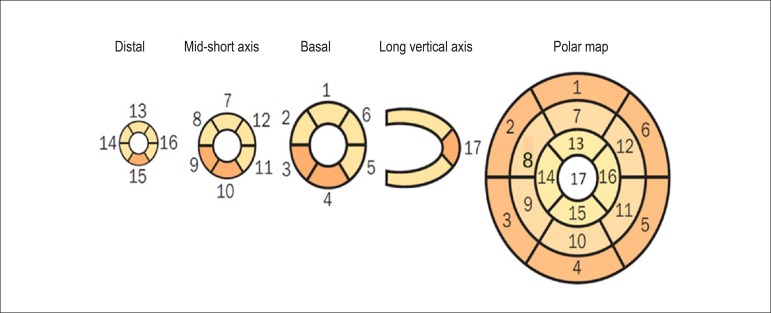
Numerical segmentation model of the left ventricular myocardium in 17 parts, considering tomographic slices of the minor and long vertical axes (distal or apical, middle, and basal or proximal portions), representing the myocardial regions; furthermore, correspondence of segments may be seen as presented in the polar map, which represents radiopharmaceutical distribution throughout the left ventricular myocardium in the form of a polar map, whose center corresponds to the apex and whose peripheries correspond to the basal portions. The correspondence between the numerical classifications and their respective segments is described in Table 18.

**Visual or qualitative analysis:** By simply inspecting images resulting from perfusion tomography and ventricular function exams (Gated-SPECT technique), it is possible to assess blood flow and regional contractility of the LV myocardium indirectly. Tomography images are reconstructed as multiple slices along the anatomical axis of the LV, defined as corresponding regions and respective relations with coronary territory. The slices are taken on the short, long vertical, and long horizontal axes (Figure 9). Characterization of uptake of the radiopharmaceutical MIBI-^99m^Tc or Tetrofosmin-^99m^Tc during both exam stages (resting and stress, 1 day protocol) and thallium-201 during the stress and redistribution phases focuses on the anterior, septal, inferior , lateral, and apical regions of the LV (Figure 9). The short-axis projection uses transverse tomographic slices of the LV, sweeping from the apex or distal portion, through the middle of the cavity, to the basal portion. All regions and subdivisions are numerically identified, in accordance with the established scoring system, with the aim of standardizing segmentary analysis of the LV myocardium for perfusion study. Division into 17 segments has consensually been accepted, resulting in less interpretation subjectivity (Figure 10 and Table 18). Different radiopharmaceutical uptake and retention patterns allow for differentiation of normal, ischemic, and fibrotic tissues. The normal myocardium has similar uptake during both the stress and resting/redistribution phases, whereas the ischemic myocardium shows reduced relative uptake in stress images and normal uptake during resting/redistribution. Fibrotic tissue, on the other hand, shows reduced relative uptake during both study phases. If fibrotic tissue coexists with an ischemic yet viable myocardium, reduced relative uptake will be observed during the stress phase, with partial improvement during the resting/redistribution phase. Hibernating myocardium will also show persistent reduced uptake, or be it, reduced uptake that is similar in both the stress and the resting phases. To differentiate it from fibrotic tissue, it is possible to perform assessment of viable myocardium with thallium-201, in which case it is sometimes necessary to add another phase or stage, namely that of late redistribution or reinjection, interpreted in the same manner.

**Table 18:** Numerical classification of segmentary division of the left ventricular myocardium into 17 parts, in cross sections (slices ?) of the minor and long-vertical axes

Regions/walls	Distal/apical portion	Middle portion	Proximal/basal portion
Anterior	13	7	1
Anteroseptal	-	8	2
Inferoseptal	-	9	3
Septal	14	-	-
Inferior	15	10	4
Inferolateral	-	11	5
Anterolateral	-	12	6
Lateral	16	-	-
Apex	17	-	-

Obs: apical region includes the apex and distal or apical portion of all walls (13,14,15,16).

**Semiquantitative analysis:** With the aim of numerically assessing the intensity of radiopharmaceutical uptake (perfusion), within the established standards (17-segment model), specific scores have been developed: **a) perfusion -** considers the following numerical scale: **0 =** normal; **1 =** mildly reduced radiopharmaceutical uptake; **2 =** moderately reduced uptake; **3 =** severely reduced uptake; **4 =** absence of radiopharmaceutical uptake. Scores of 3 or 4 are normally associated with coronary stenosis of > 90%. Therefore, the higher the number of affected segments is; the more extensive the process; the higher the summed scores, and the greater the severity will be. This has an unquestionable prognostic value for patients with CAD. The following calculations are achieved by the sum of values attributed to each segment: the sum of the values attributed to each segment during the stress phase is known as the “summed stress score” (**SSS**); this is repeated during the baseline or redistribution phase to obtain the “summed rest/redistribution score” (**SRS**). The difference between the SSS and the SRS is known as the “summed difference score” (**SDS**). According to Hachamovitch et al.^56,57^ numerical **SSS** values **< 4** are considered *normal*; between **4** and **8**, *mildly abnormal*; between **9** and **13**, *moderately abnormal*; and **> 13**, *severely abnormal*. It is worth emphasizing that **SSS** values **< 4**, which may not necessarily be zero, are understood as normal, because there are myocardial regions which show lower radiopharmaceutical concentrations in and of themselves and may, consequently, receive values other than zero.

**Quantitative analysis:** *Polar maps* are two- or three-dimensional reconstructions of the LV, initially elaborated with the proposal of encompassing relative radiopharmaceutical distribution throughout the heart in a single image. They are shown in circular form, resembling a target, for which reason they are also known as “bull’s eye plots.” Radiopharmaceutical uptake, which is representative of perfusion, is shown on a color scale, with the LV apex occupying the center of the target, while basal regions of the heart are represented by the outermost circle of the target (Figure 10). Programs capable of reconstructing these images also allow for percentage quantification of areas with reduced uptake by comparing the images to a databank of normal individuals of the same age and sex. Perfusion defects may also be quantified by the number of pixels in a determined region and by existing standard deviations in relation to normal perfusion areas.

We may also obtain polar maps with parameters relative to ventricular function, such as LV wall motility and systolic thickness. These methods of quantitative analysis serve as complements to assist in qualitative or semiqualitative visual analysis.

**Evaluation of ventricular function with perfusion agents:** In a manner analogous to that described for perfusion study, segmentary contractile analysis of the LV makes use of motility and systolic thickness scores for each segment, also considering division into 17 segments, visualized in the cross sections of the minor axis (distal, mid-cavity, and proximal regions) and the long vertical axis (anteroapical and inferoapical regions). Numerical values are attributed. Analysis of motility of LV walls is performed directly on the computer monitor, making the subendocardial contour visible. Analysis of systolic thickness should be directed to the color scale chosen for a group of images. When thickness is within normal limits, the color increment is observed toward the bottom of the scale. Furthermore, it is possible to obtain percentage of thickness in each region. For distal and middle slices of the minor axis, as well as for the apex, average normal thickness is around 40%, with a **score of zero (0)** Thicknesses between 30% and 40% are interpreted as borderline; those between 20% and 30% receive a **score of 1** (mild reduction); 10% to 20%, a **score of 2** (moderate to severe reduction); and less than 10%, a **score of 3** (absence of thickness)**.** In the proximal (or basal) cross section of the minor axis, thickness of around 20% is considered normal. A *score of 1* is not used, but rather only scores of 2 and 3. Abnormalities in motility and thickness generally go hand-in-hand, with slight differences in gradation between the two (Table 19) and in the resulting sums. In some cases, we may observe discrepancy between results, for instance, following revascularization surgery and in the presence of left bundle branch block, in which the motility of the interventricular septum is compromised, whereas thickness is not.

**Table 19:** Segmentary analysis of left ventricle motility and systolic thickness by single photon emission computed tomography synchronized with ECG (Gated-SPECT)

Score (points)	Motility	Thickness
0	Normal	Normal
1	Mild hypokinesis	Mild reduction
2	Moderate hypokinesis	Moderate/severe reduction
3	Severe hypokinesis	Absence of thickness
4	Akinesis	-
5	Dyskinesis	-

Whenever possible, analysis of ventricular function should be performed at the baseline phase and after stress, with the purpose of detecting additionally indicative alterations in stunned or hibernating myocardium. The validated scores, which have been previously described, are recommended. Regarding the effect of global analysis of LV systolic function, LVEF is the parameter with the best reliability, and scores predominantly concentrate on segmentary analysis.

Furthermore, the subjective or qualitative assessment of images regarding results related to myocardial perfusion study, which have been previously described (*homogenous distribution or normal uptake of the radiopharmaceutical in the myocardium; transient low uptake suggestive of ischemia; fixed low uptake suggestive of fibrosis; partially reversible low uptake suggestive of ischemia associated with fibrosis*), should take the presence of the following types of artifacts into account:

- Technical artifacts, resulting from inadequate image processing.
- Motion artifacts.
- Attenuation artifacts, due to interposition of mammary or diaphragmatic tissue (intestinal handles), which are factors that interfere with the specificity of the exam.

## 6. Current Utilization of Myocardial Perfusion and Ventricular Function Studies with Radiopharmaceuticals as Part of the Medical Decision-Making Process

MPS with the injection of radiopharmaceuticals associated with ET or the administration of coronary vasodilators is an established method for diagnosis and risk stratification of obstructive CAD,^91-100^ with the aim of guiding more effective clinical management of patients as part of the medical decision-making process.^101,102^ It currently integrates other non-invasive cardiovascular imaging techniques, such as Doppler echocardiography with color flow mapping, CS, coronary angio-CT, PET, and cardiac magnetic resonance (MR), to characterize risk and functional expression of atherosclerotic disease.^103^ Accuracy of method was, until recently, based on invasive coronary cineangiography, considered the standard for this comparison. The following stand out as highly relevant aspects, considered of paramount importance to the modality:

- Obtaining variables and parameters that are fundamental to incremental prognostic characterization of CAD, such as electrocardiographic response to exercise, functional capacity , chronotropic response, blood pressure, et al. Of all forms of stress associated to MPS, the exercise testing is, without a doubt, the one that adds the greatest amount of information.^104-106^
- When analyzing perfusion images, the possibility of quantifying area of myocardial ischemia or myocardium at risk has advanced a great deal over the past decades, undoubtedly participating in risk stratification and medical decision making for stable CAD, where it provides assistance for the choice between maintaining clinical treatment and interventional treatment.^107^ Even in the absence of randomized studies published to date, which might reaffirm this information (the Ischemia Study - report to the Addendum of this guideline),^108^ the evidence which has currently been accumulated and made available documents better evolution in patients with severe ischemic burden who undergo myocardial revascularization.^109-112^
- When analyzing ventricular function images, indirect observation of thickness and motility of the LV walls and comparison of resting and exercise ejection fractions greatly improve the method’s specificity for characterizing true ischemia and aggregate incremental prognostic value with the definition of markers of severity, such as transient ischemic dilatation (TID), representing ventricular dysfunction and / or subendocardic ischemia induced by applied stress.
- The ability to infer coronary flow reserve under applied stress or stimulus with elevated accuracy, superior to other conventional methods, with the exception of PET, is the most important physiological parameter for characterization of ischemia and the medical decision-making process, currently available in clinical practice with direct invasive measures of FFR and instantaneous wave-free ratio (IFR). Software currently in development for calculating coronary reserve in association with SPECT methodology and other non-invasive methods will likely aggregate unquestionable value to appropriate clinical or interventional treatment choices, in the near future, encompassing not only obstructive atherosclerotic disease, but also physiopathological conditions, including microvascular disease and endothelial dysfunction in the scenario of ischemic heart disease.^85,113-115^

**Figure 11:**
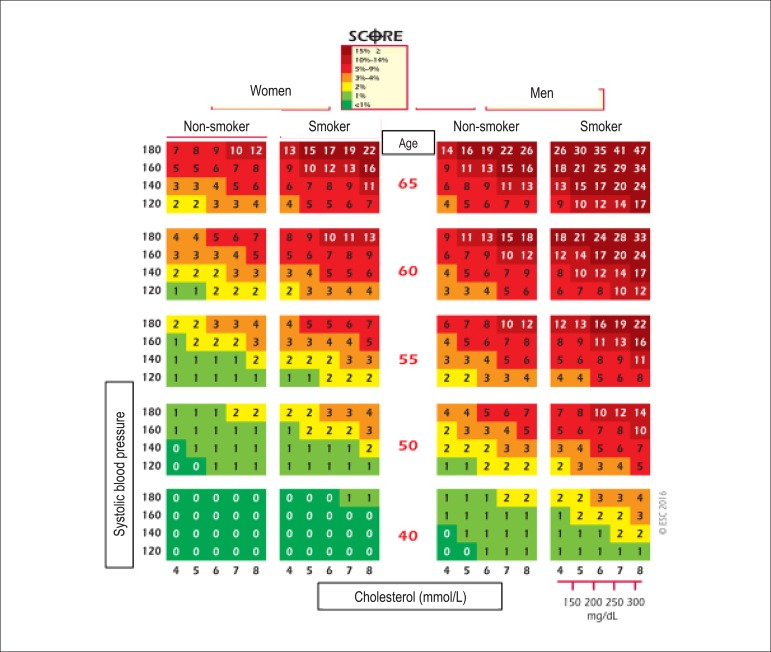
Calculation of 10-year risk of fatal cardiovascular event, in populations of countries with high cardiovascular risk, considering the risk factors of age, sex, smoking, total cholesterol, and systolic blood pressure. Cart from the SCORE (Systematic Coronary Risk Evaluation) Study. The color scale varying from red to green corresponds to percent risk over 10 years, described in the upper part of the Table. Adapted from Piepoli MF et al.^120^

The main applications with the best cost-effectiveness are shown in patients with intermediate pre-test probability of CAD, estimated based on the integration of clinical variables which have been established and documented in Brazilian and international guidelines, with their respective recommendations and levels of evidence (Tables 20 and 22 and Figure 11). Ideal diagnostic and prognostic capacities have for decades been considered with regard to severe coronary lesions. Nonetheless, exercise testing are indicated as the ideal and preferred association for myocardial scintigraphy, considering the physiological nature of the form of applied exercise and the established clinical value of the variables obtained during and after work.^116-120^

**Table 20:** Recommendations for cardiovascular risk assessment, considering the presence or absence of known risk factors. European Guidelines on cardiovascular disease prevention in clinical practice^119^

Recommendations	Class of recommendation	Level of evidence
CV risk assessment in individuals with family history of premature CV disease, family history of dyslipidemia, major risk factors (smoking, HBP, DM, raised lipid levels), or specific comorbidities that increase CV risk.	I	C
Repeat risk assessment every 5 years; repeat more often in individuals with risks close to levels which treatment is mandatory	I	C
Consider CV risk assessment in men > age 40 and women > age 50 or post-menopausal with no known risk factors	IIb	C
CV risk assessment in men < age 40 and women < age 50 with no known risk factors is not recommended	III	C

C: level of evidence based on consensus of expert opinion and/or small studies, registries, or retrospective studies; CV: cardiovascular; DM: diabetes mellitus; HBP: High blood pressure; I, IIb, and III: class of recommendation.

**Table 22:** Percent probability of obstructive coronary artery disease, considering the presence of chest pain, sex, and age. Comparison between LR and HR patients. Adapted from Gibbons RJ et al. and the Brazilian Cardiology Society's Third Guidelines on Exercise Testing^117,119^

Age	Non-anginal chest pain				Atypical angina				Typical angina			
	Men		Women		Men		Women		Men		Women	
	LR	HR	LR	HR	LR	HR	LR	HR	LR	HR	LR	HR
35	3	35	1	19	8	59	2	39	30	86	10	78
45	9	47	2	22	21	70	5	43	51	92	20	79
55	23	59	4	25	45	79	10	47	80	95	38	82
65	49	69	9	29	71	86	20	51	93	97	56	84

CAD: coronary artery disease; HR: high risk (smoking, diabetes, or dyslipidemia); LR: low risk (without smoking, diabetes, or dyslipidemia).

Pharmacological tests performed in nuclear cardiology represent good alternatives for assessing patients with physical limitations or clinical limitations to undergoing efficient exercise testings. They include approximately 20% to 30% of all cases referred for scintigraphy and approximately 50% of elderly patients.^121^ In these circumstances, the drugs utilized are dipyridamole, adenosine,^69,122,123^ and regadenoson.^124^ (Additional details described in Methodology.)

Similarly, in practice, when comparing conventional algorithms used for established the probability of CAD and major adverse events in stable chest pain patients or asymptomatic patients, such as Framingham Risk Score (FRS), PROCAM, SCORE, Diamond Forrester,^125^ or Global Risk;^126^ the estimated prevalence (EP) of the disease is observed to be significantly higher than the observed prevalence (OP), when coronary angio-CT is used to characterize luminal obstruction, ≥ 50% and ≥ 70%, respectively. In this situation, an international multicenter study (CONFIRM)^127^ of 14,048 consecutive patients with clinical suspicion of coronary obstructive atherosclerosis who underwent angio-CT showed that, in all age and sex categories, guidelines for calculating probability overestimated prevalence in the general population (51% EP × 18% OP for lesions ≥ 50% and 42% EP × 10% OP for obstructions ≥ 70%, p < 0.001), directed by accentuated differences between patients with typical angina (86% EP × 29% OP for lesions ≥ 50%) and atypical angina (47% EP × 15% OP for lesions > 50%). Considering this information to be true, more evidence has arisen within the literature in the search for new markers which might aggregate value and assist in more objective and realistic restratification of cardiovascular risk, with specific guidelines^128^ dealing with critical questions regarding, for instance, what types of evidence will contribute to risk assessment or reclassification when new markers are added to traditional scores, with emphasis on functional capacity and CS (Table 23).

**Table 23:** New markers for cardiovascular risk stratification

• High-sensitivity CRP
• Apolipoprotein B
• Glomerular filtration rate
• Microalbuminuria
• Ankle-brachial index
• Family history
• Functional capacity
• Mean carotid intima thickness
• Coronary calcium score

CRP: C-reactive protein.

### 6.1. The Application of Bayes’ Theorem to Analysis of Myocardial Perfusion Images with Radiopharmaceuticals

**Figure 12:**
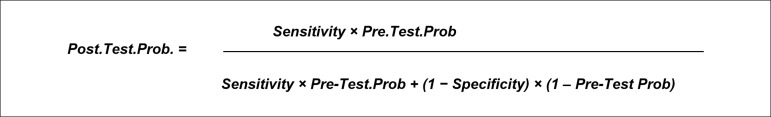
Formula for calculating post-test probability of a disease according to Bayes’ theorem.

Even when isolated analysis of images is used to describe perfusion findings, interpret data, and write reports, medical comments and conclusions should be the result of the integration of all available pretest clinical data and data obtained during the performance of the stress or associated stimulus test within the denominated incremental prognostic value. In this sense, Bayes’ theory of conditional probability or the application of Bayesian principles assists in decision making by establishing that the risk of an event occurring after a test is influenced by the sensitivity and specificity of an applied method, as well as the pretest prevalence of the disease, all of which are incorporated into the estimation of post-test probability for characterization of myocardial ischemia and, consequently, CAD (Figure 12).^129,130^

In this way, the diagnostic ability of a test is related to the population type selected, and it is may create tendencies or biases. For example, the selective referral of patients with “positive,” “altered,” or “ischemic” results for coronary cineangiography studies, in conjunction with few referrals of individuals with negative results, increases the chance of false-positive results with respect to true-negative results. This would be an equivocal methodology for evaluating the accuracy of a test, artificially decreasing the method’s specificity or its ability to select healthy individuals within a population.^131,132^ On the other hand, sensitivity will expressively increase in patients referred with a high prevalence of symptoms.

**Figure 13:**
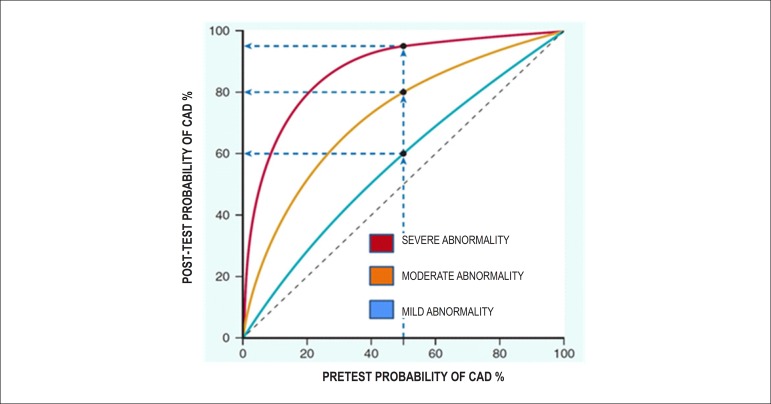
Importance of amount of myocardium at risk (extension and intensity of ischemia) on myocardial perfusion imaging with radiopharmaceuticals (^99m^Tc-sestamibi or thallium-201) to post-test probability of coronary artery disease (CAD). For a given pretest probability (50% indicated in the graph), the post-test probabilities will be significantly higher according to imaging findings. With the condition of high-risk ischemia or > 20% extent of ischemic myocardium, the clinical implications for decision making become practically independent of pre-test probability of CAD.

Source: Adapted from Udelson JE et al.^130^

Many possibilities may be present for medical management within different prevalences of clinically estimated CAD, emphasizing that the diagnostic power of conventional exercise testing or tests associated with MPS is at a maximum when the pretest probability of CAD is intermediate. However, for a given pretest probability, the post-test probability increases progressively with the severity of the alterations found, such as the amount of myocardium at risk or the sum of extent and intensity (ischemic burden) of perfusion modifications in the perfusion images with radiopharmaceuticals. In the extreme case of a study with severe abnormalities, post-test probability will be elevated regardless of pretest probability (Figure 13).^130^

**Figure 14:**
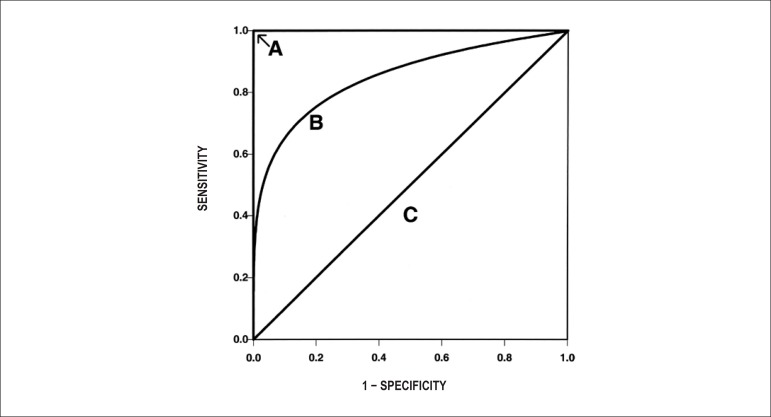
Hypothetical examples of ROC curves, with: the area under the curve representing maximum or perfect diagnostic accuracy of the standard utilized (curve A; AUC = 1); “real” area under the curve representing good efficiency of the method used, often found in clinical practice (curve B; AUC = 0.85); the 45-degree diagonal line corresponding to random chance (curve C; AUC = 0.50), with the area under the ROC demonstrating the averages of diagnostic accuracy across a spectrum of cutoff values. On rare occasions, the estimated AUC is less than 0.5, indicating that the test being evaluated performs worse than random chance. Adapted from Zou KH et al.^133^

Furthermore, not only Bayesian analysis, but also statistical techniques that use multivariate analysis to estimate post-test risk may also provide important diagnostic information, with the following advantages: they do not require the tests to be independent of each another or the diagnostic indexes (sensitivity and specificity) to remain constant in populations with different disease prevalences. Thus, in the condition of continuous-scale diagnostic tests, changes in percentages of sensitivity and specificity should be taken into consideration when cutoff values for classifying individuals with and without a disease vary. Some results may even be expressed as the sum of sensitivity and specificity for an “optimal” cutoff value. However, owing to the fact that an optimal cutoff value is not relevant to a specific application, it is recommendable to plot these indexes under a range or scale of values of interest, generally distributed under a receiver operating characteristics (ROC) curve, expressed in a 2-axis graph, where the y axis represents sensitivity and the x axis = 1 − specificity, for variable cutoff values (Figure 14).^133^

### 6.2. Value of the Diagnosis-Prognosis Binomial to Integrated Assessment of Perfusion Images

The presence of transient or reversible defects in radiopharmaceutical uptake reflect ischemia, which is in itself associated with greater incidence of future events, when comparing normal images or images with persistent perfusion defects. Thus, in patients with suspected or proven chronic coronary disease, estimation of the quantity of myocardium at risk as assessed by semi-quantitative and quantitative analyses, extent, intensity, and degree of reversibility of existing defects, as well as measures of LVEF following physical or pharmacological stress, have prognostic value, indicating risk of events during clinical follow up.^134-138^ Other scintigraphy markers of severity may stand out, such as apparent transient dilation of the LV, induced or accentuated by exercise or pharmacological tests,^139,140^ which may translate to extensive subendocardial ischemia, in addition to high pulmonary uptake, translating to LV dysfunction. Furthermore, increased uptake in the walls of the right ventricle (RV) in multi-arterial patients whose lesions are predominantly in the left coronary territory, may suggest an imbalance in perfusion between ventricles.^141,142^

Considering the scope and accumulated experience of MPS with radioisotopes in diverse clinical scenarios relating to CAD, guidelines and consensuses have suggested the main applications based on levels of evidence in the literature, and created scores that numerically classify indications as inappropriate; possible, but questionable; and appropriate^143,144^ (additional details described in the *Indications* section).

### 6.3. Radiopharmaceuticals for Performance of Myocardial Perfusion Scintigraphy and Image Generation and Perfusion Defects

Nuclear cardiology is connected to the assessment of cardiovascular physiology, currently encompassing metabolism, innervation, myocardial perfusion, ventricular function, and synchronism. It has a capability for early detection of cardiovascular physiopathological alterations, allowing for interventions which may interrupt or revert the disease condition before structural alterations are established in a definitive, evolutive, and irreversible manner. To represent cardiac physiology, images are formed using the principle of radiotracers or tracers,^29^ in which the exchange of stable atoms with their isotopes does not alter the biological properties of the organism where the images are being obtained.

Radioactive labeling is performed with minimal quantities of chemical substances, resulting in a radiopharmaceutical that may be used to truly represent physiological or biochemical state of unlabeled molecules . In this manner, alterations to the physiology being evaluated and toxicity effects do not occur. These characteristics are different from other imaging methods which use elevated concentrations of chemical substances to create sufficient contrast and, consequently, obtain images of the functional situation and anatomical aspects of the organ under study.^145^ The images in this specialty are digital; they either use “pixels” as units of measurement for resolution or are transformed into a digital matrix, emphasizing that “pixel” values of images of the ventricular myocardium are directly proportional to physiological cardiovascular properties. Physical phenomena such as the “Compton scattering effect,” the “photoelectric effect,” and geometric distortions should, however, be considered,^146^ given that they tend to interfere with direct proportionality, in a manner that is decreasing as equipment and image reconstruction techniques technologically evolve. Furthermore, another factor related to acceptance and preference of nuclear cardiology for detecting myocardial perfusion defects is the elevated, superior resolution contrast (allowing for differentiation between normal and decreased perfusion) in comparison with other imaging methods,^147,148^ even considering lower spatial resolution. There is also a peculiar aspect, namely, that the myocardium (organ of interest) appears emphasized due to the greater brightness in comparison with underlying structures (background) and, consequently, provides excellent signaling, which facilitates the development of integrated, computerized algorithms for SPECT and PET techniques. These programs, which automatically process and objectively quantify images, have good comprehension, and they are well validated and internationally utilized.^149-151^ From the conceptual point of view, it is necessary to grasp that scintigraphy image generation is based on relative uptake of the radiopharmaceutical in the myocardium of the LV, when it is injected intravenously during physical exercise or pharmacological tests. The comparison of radiopharmaceutical uptake between ventricular walls is expressed in images based on a color scale, created by specific computer programs which, in addition to allowing for subjective analysis of perfusion, make it possible to conduct semi-quantitative and quantitative evaluation of affected myocardial area. During visual evaluation of scintigraphy images, the following are taken into consideration: homogenous distribution patterns or normal radiopharmaceutical uptake in the myocardium, transient low uptake suggestive of ischemia, fixed low uptake suggestive of fibrosis, and partially reversible low uptake suggestive of ischemia associated with fibrosis^24,152^ (Examples are provided in the *Methodology* and *Tutorial Cases* sections).

## 7. Evaluation of Patients with Potential Acute Coronary Syndrome - Algorithms in the Chest Pain Unit

### 7.1. Introduction

**Figure 15:**
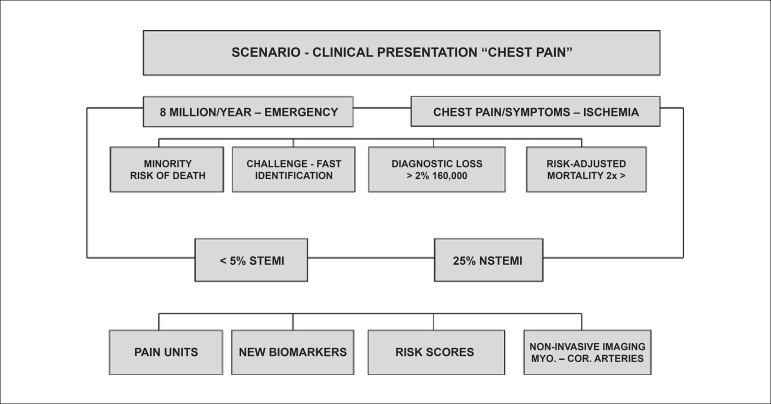
Chest pain spectrum in emergency units, with clinical implications, forms of presentation of acute coronary syndrome and available methods for investigation and risk stratification. Cor: coronary; Myo: myocardium. NSTEMI: non-ST-segment-elevation myocardial infarction; STEMI: ST-segment-elevation myocardial infarction.

Source: Adapted from Amsterdam EA.^154^

Continuous chest pain is one of the most common symptoms in emergency units, accounting for approximately 8 million annual visits in the USA.^153^ Although approximately 50% of patients are admitted for diagnostic definition, only 30% of visits will correspond to the condition of acute coronary syndrome (ACS), 2% to 4% of whom will be inappropriately discharged from the hospital (Figure 15), leading to serious risks of severe events, in addition to legal-medical problems. Considering these implications, as well as hesitation to discharge patients with acute myocardial infarction (AMI), assessment of patients with atypical chest pain in emergency unit has emphasized admission for posterior clarification and risk stratification. With the development of more sensitive cardiac biomarkers in conjunction with more precise non-invasive exams and validated clinical parameters, early identification of low-risk patients has been carried out more rapidly. In this process of diagnostic and prognostic assessment, the following play an important role: resting ECG, cardiac enzymes, and non-invasive exams such as ET, MPS, Doppler echocardiogram, and coronary angio-CT, in addition to cardiac resonance in specific cases. The choice of recommended imaging method should be based on the procedures available, local institutional experience, and present clinical situation. The exam with the highest diagnostic accuracy and negative predictive value (NPV) will offer more precise risk stratification, which is fundamental to decision making regarding need for hospital admission or safe discharge from the emergency unit.

In addition to the 2 physiopathological conditions described (Non-ST segment Elevation Myocardial Infarction - NSTEMI and ST segment elevation myocardial infarction - STEMI), unstable angina also stands out, which does not feature myocardial necrosis as an initial consequence.^155,156^ Nevertheless, unstable plaque and evolutive phenomena of erosion and rupture may progress to infarction and related complications, such as severe arrhythmias, ventricular dysfunction, and death. Conditions of vasospasm, in epicardial coronary arteries or with microvascular disease, have additionally been implicated in ACS without thrombosis and myocardial infarction, in the absence of obstructive lesions.^157-159^ It is, finally, important to emphasize that, in patients with documented ACS and intermediate- to high-risk patients, invasive coronary cineangiography and percutaneous revascularization represent the most frequent forms of initial assessment, and non-invasive imaging methods are reserved for clinically stable situations and low- to intermediate-risk patients, with the aim of reclassifying risk, diagnosis, and stratification in the post-event phase.^160,161^

### 7.2. Goals for Evaluating Acute Chest Pain and Participation of Non-invasive Methods in Assessing ACS^154,162,163^

- Precise diagnosis for appropriate conduct in UA or AMI, whether with clinical treatment or invasive strategy via catheterization and angioplasty.
- Early, safe discharge from the hospital if clinical data and exams show no abnormalities. Probability of severe cardiac events < 1% over 30 days of evolution following discharge from the emergency unit or hospital.

Following serial evaluation of ECG, without modifications, in addition to normal cardiac enzymes and clinical situation characterized as low- to intermediate-risk, non-invasive functional exams may play an important role in risk stratification of patients with acute chest pain. The choice of MPS, cardiac resonance, or angio-CT will depend on the objective and the clinical question to be answered.

**Exercise testing:** constitutes an important strategy for assessing patients with suspected ACS following stabilization, and it aids prognosis and medical management . Patients with chest pain in the emergency room, once they have been identified as low-risk, may undergo ET, a normal result of which confers low annual risk of cardiovascular events, allowing for earlier and safer discharge from the hospital.^164^ Brazilian and international guidelines recommend ET as a first-choice exam for risk stratification in patients who are able to exercise, as the procedure is low-cost and widely available, and it has a low rate of complications, similar to that of tests conducted in normal conditions.^165^ A treadmill or a cycle ergometer may be used, following appropriate protocols for the patient’s clinical conditions, such as the ramp protocol or the modified Naughton or Bruce protocol. Logistics related to performing ET in the emergency unit may, however, be compromised as a result of unavailable operational personnel or infrastructure during certain periods (e.g. weekends or night shifts).

#### Summary of indications for ET in ACS (characterize low-risk after initial clinical stratification)

- Baseline ECG and biomarkers (necrosis) without alterations.
- Absence of symptoms (precordial pain or dyspnea).
- Hemodynamic stability and adequate conditions for physical effort.

If ET results are normal and the patient has shown good functional capacity, other procedures may be unnecessary, in virtue of the test’s high NPV.^165^

#### Summary of Recommendations and Evidence

#### Class of recommendation I. Level of evidence: B

- Low-risk (clinical and ECG) patients with normal biomarkers should be referred for exercise test after 9 to 12 hours. Within the routines of chest pain units, these exams may be used as discharge criteria.

If it is not possible to perform ET or if ECG is uninterpretable, the patient may undergo provocative tests for ischemia associated with non-invasive imaging.

**Doppler echocardiogram (ECHO):** This is fundamental for evaluating patients with acute chest pain^166-168^ and evolving ACS, initially considering LVEF, segmentary contractile alterations, and the presence of thrombi, in addition to mechanical complications (rupture of interventricular septum or papillary muscles) that result in severe events, such as cardiorespiratory arrest. Moreover, this method may also evaluate chest pain with non-coronary etiology, such as pericardial disease, hypertrophic cardiomyopathy, aortic dissection in the presence of renal insufficiency that makes it impossible to perform angio-CT, and others. In addition to assessing the presence and extent of ventricular dysfunction, it is able to quantify severity of valvular abnormalities that may be present and associated with ischemic etiology.

#### Summary of Recommendations and Evidence

##### Recommendation class I

- Transthoracic ECHO is indicated when there is clinical suspicion of aortic and pericardial diseases, pulmonary embolism, and valvulopathies (*level of evidence: C*).
- In cases with complications resulting from unstable ACS, such as interventricular communication and mitral insufficiency (*level of evidence: C*).
- Stress echocardiography is considered an alternative to exercise testing in patients who cannot exercise (*level of evidence: B*).

##### Recommendation class IIa

- Patients suffering from chest pain - resting ECG to determine whether or not pain is of ischemic origin (*level of evidence: B*).
- Patients with uncomplicated anterior wall AMI, with the objective of determining the exact size of the ischemic lesion (*level of evidence: B*).

In stable patients with evolving ACS, echocardiography associated with pharmacological stress before hospital discharge may identify induced ischemia and assist in risk stratification and medical management of immediate follow-up (6 to 12 weeks), especially if LVEF values are below 40%.

**Coronary angiotomography:** Many studies have shown that coronary angio-CT is an important tool for evaluating acute chest pain, especially in low- to intermediate-risk patients.^169-172^ It is a safe procedure for diagnosing ACS, and it is able to reduce intra-hospital follow-up time and contribute to cost reduction. In the Rule Out Myocardial Infarction by Cardiac Computed Tomography II (ROMICAT II) Study, duration of hospital stay was significantly lower in patients stratified via angio-CT in comparison with the group submitted to conventional evaluation (23.2 ± 37 hours *vs*. 30.8 ± 28 hours).^173^ There was also a significant increase in percentage of patients discharged from the emergency unit in the group stratified with this method (46.7% *vs*. 12.4% p < 0.001), in spite of higher costs associated with angio-CT and the greater tendency to refer patients for catheterization and revascularizations.

**Figure 16:**
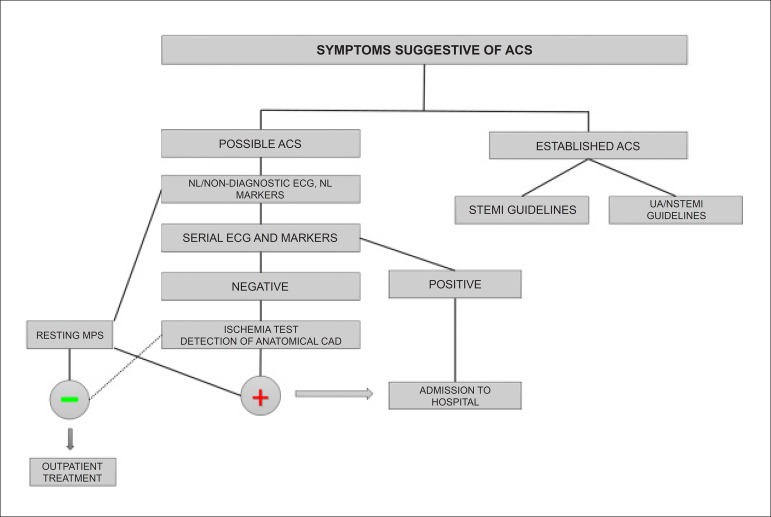
Clinical scenarios of patients who present with chest pain at emergency units. Situations of ACS diagnosed as STEMI and UA/NSTEMI correspond to orientations established by pertinent guidelines. In the condition of possible or suspected ACS, the previously described sequences of diagnostic investigation and stratification are recommended. ACS: acute coronary syndrome; CAD: coronary artery disease; MPS: myocardial perfusion scintigraphy; NL: normal; NSTEMI: non-STsegment elevation acute myocardial infarction; STEMI: ST-segment elevation acute myocardial infarction; UA: unstable angina.

Based on recent publications, low- to intermediate-risk patients with acute chest pain, non-diagnostic ECG, and negative markers of necrosis have Class-I recommendation and level of evidence A for undergoing angio-CT, especially considering the method’s NPV. There are, nevertheless, limitations in the presence of STEMI and NSTEMI (Figure 16) (with the exception of coronary dissection) and known CAD or prior revascularization where the existence of intracoronary prostheses (stents) and calcium may negatively influence the exam’s specificity for its proposed aim, leaving the possibilities of functional evaluation and global repercussion. Finally, it is necessary to consider exposure to elevated doses of radiation and lower image quality for the exclusion of pulmonary embolism, aortic dissection, or ACS (triple rule-out).^174^

**Myocardial perfusion scintigraphy (MPS):** Within the scope of its applications (*See the Indications chapter*), the following stand out: indirect evaluation of coronary reserve and consequent estimation of functional significance of coronary stenoses, evaluation of the efficacy of therapeutic interventions, and stratification of ACS risk. One of the principal indications for MPS during the first 12 hours of symptom onset is to decide whether or not to hospitalize a patient with chest pain and suspected CAD, when ECG was normal or had non-specific alterations. Resting MPS, when it is performed in an early phase of attendance and considered low-risk, determines a low index of future cardiac events. Recent studies have demonstrated that, when ACS is suspected, the use of resting perfusion images with radiopharmaceuticals^175,176^ is also associated with shorter hospital stays and lower costs, and it is additionally able to reduce unnecessary hospitalizations.^177,178^ Furthermore, numerous observational studies have demonstrated a high NPV for normal resting perfusion images, with the objective of ruling out AMI or short-term cardiac events.^179^ In a study by Schaeffer et al.,^180^ 479 patients underwent resting MPS and were followed for 16 months. Of these patients, 434 had “normal” resting MPS, and 45 had “abnormal” resting MPS. In the normal group, only 3 patients (0.7%) had severe cardiac events, showing a NPV of 99.3%.^180^ Equally, multiple evaluations have demonstrated the efficacy and safety of MPS with SPECT for assessing patients with chest pain in the emergency unit.^181-183^ Population samples involved, however, were more heterogeneous and had higher numbers of risk factors for CAD. MPS effectively foresaw which patients would require coronary angiography. Nabi et al. related that 38.3% of patients with abnormal MPS underwent revascularization, while 0.9% of patients with normal MPS subsequently underwent coronary intervention. Of patients with myocardial area at risk (ischemia) involving > 10% extent in perfusion images, 55% underwent revascularization.^182,183^

The study known as the PREMIER trial by N Better et al.^184^ evaluated the performance of resting MPS in investigating chest pain in the emergency room with 356 low- to intermediate-risk patients, from 8 developing countries, including 2 Brazilian centers. The primary outcome considered included the compound events of death, non-fatal AMI, recurring angina, and coronary revascularization over 30 days, and the results reaffirmed the association between normal images and a good NPV (99.3%) for severe events (death or AMI).^184^ Moreover, it is worth highlighting that the presence of resting perfusion alterations (abnormal scintigraphy) was the only variable independently associated with the primary outcome (adjusted OR = 8.19, 95% CI: 4.10-16.40, p = 0.0001), with even higher expression when only patients who received injections during episodes of pain were considered (adjusted OR = 17.35). On the other hand, results considered high-risk indicated worse prognoses for future cardiac events (death, AMI, myocardial revascularization surgery, or percutaneous intervention).^185,186^

The Emergency Room Assessment of Sestamibi for Evaluation of Chest Pain (ERASE) Study, which evaluated patients with ACS and normal or non-diagnostic ECG who were still in the emergency room, observed admission rates of 54% in patients who underwent MPS and 63% in other patients, suggesting that the initial strategy of resting scintigraphy is satisfactory, based on the fact that it demonstrates good risk stratification capability.^184,186^

International guidelines recommend the use of resting myocardial perfusion images for acute chest pain as a class I recommendation with level of evidence A for risk stratification of patients with suspected ACS and non-diagnostic ECG.^187,188^

**Time of radiopharmaceutical injection:** The main applications of MPS within the first hours of a patient’s arrival at the hospital are:

- *Radiopharmaceutical injection* (technetium-99m-labeled sestamibi / MIBI or tetrofosmin, also known as ^99m^Tc-sestamibi and ^99m^Tc-tetrofosmin), *while resting, during an episode of chest pain, with normal or non-specific ECG*, with the objective of rapid diagnostic definition.
- *Radiopharmaceutical injection while resting, in the absence of chest pain, with normal or non-specific ECG*, when the symptom has ceased less than 6 hours prior, but preferably within the 2 preceding hours. Wackers et al.^179^ have demonstrated that, in cases where injections are administered up to 6 hours after pain, in ACS, there is an 84% incidence of perfusion abnormalities; this decreases to 19% when intravenous radiopharmaceutical administration occurred between 12 and 18 hours after the last episode of pain. Kontos et al.^176^ found no reduction in sensitivity for identifying patients who evolved to AMI or revascularization when the injection was administered during the moment of pain or up to 6 hours after the cessation of the symptom.

Taken as a whole, perfusion imaging with radiopharmaceuticals for evaluation of acute chest pain does not present any formal contraindications; it is well tolerated by most patients, and the quantitative assessment of information about ischemia is of special importance to the clinical decision-making process.

**PET:** In a retrospective study conducted with more than 7,000 patients who presented at the emergency unit with chest pain,^189^ 92.5% of patients with positive stress or resting PET were diagnosed with ACS, by cardiac catheterization, electrocardiographic alterations, or positive cardiac biomarkers. In patients with reversible perfusion defects while resting or under stress (positive PET) who underwent cardiac catheterization, 87% were considered to have significant CAD. For patients without reversible perfusion abnormalities whose exams were classified as negative, no deaths were informed during the 30-day follow-up period. PET has a significantly better spatial resolution and higher sensitivity when compared to MPS. In Brazil, the elevated costs and low availability of this technology for patients with ACS are, however, limiting factors to its routine use.

**UA and NSTEMI:** Patients whose clinical conditions indicate a high risk of AMI or UA should undergo hemodynamic study. For those with low to intermediate risks whose unstable angina (UA) has been clinically stabilized, MPS has shown an important value for risk stratification.^190^ Additionally, it assists in diagnosis and medical management. It is recommended within the first hours of the patient’s arrival at the hospital. Cases with a history of chest pain, whose biochemical markers, nevertheless, show no alterations, with normal or non-diagnostic ECG are considered candidates. When patients are symptomatic or immediately following cessation of symptoms, intravenous administration of Sestamibi-^99m^Tc or Tetrofosmin-^99m^Tc occurs, preferably while resting, followed by image acquisition directly after or up to 6 hours following radiopharmaceutical injection. To perform MPS associated with physical stress or vasodilator drugs in appropriate patients (those with low to intermediate risks), symptoms should be under control or angina should be stabilized for at least 48/72 hours.^191^ Patients without ischemia or infarction and preserved LV function have good prognosis and they may be managed conservatively, while patients with significant ischemia induced during associated tests should be referred for invasive exams.

The simultaneous information provided by myocardial perfusion and ventricular function via scintigraphy synchronized with ECG (Gated-SPECT) is of fundamental importance, given that both the absolute LVEF value and the extent and intensity of perfusion defects have prognostic value for the occurrence of future cardiac events.

Finally, with the advent of chest pain units and emergency units for the evaluation of patients with suspected ACS and with new tools which have become available, such as clinical risk scores, biomarkers, or multimodalities (non-invasive exams), algorithms have been proposed to support investigation and treatment of different clinical presentation scenarios (Figure 16). Their implementation aims to improve cost-effectiveness and to lower morbidity and mortality in the management of this subpopulation, within the spectrum of ischemic heart disease.

#### Recommendations and Evidence

##### Class I

Stress and resting MPS as an alternative to cases with limitations to ET (level of evidence: C).

##### Class II

Patients suffering from chest pain may be evaluated via resting MPS to determine whether the pain is of ischemic origin or not (level of evidence: A).

**STEMI:** Coronary cineangiography is a priority indication for initially attending patients with ACS and ST-segment elevation, seeing that coronary reperfusion is the primary objective. However, in cases in which clinical condition, ECG, and biochemical markers are inconclusive, MPS may aggregate incremental diagnostic and prognostic value. These situations are generally characterized by atypical clinical conditions in patients with non-specific electrocardiographic alterations in the ST segment, left bundle branch block, and, mainly, in those who are attended before or after the onset of the condition, while they are already outside of the ideal period for dosage of biochemical markers.

#### Recommendations and evidence for stress and resting MPS following STEMI

##### Class I

- Before being discharged from the hospital, in stable patients who have not undergone coronary cineangiography for risk assessment and therapeutic decision making (level of evidence *B*).
- Complementary evaluation following coronary cineangiography, in cases where there are doubts, with the aim of defining and quantifying ischemia for eventual myocardial revascularization (level of evidence B).

## 8. Positron Emission Tomography in Cardiology

### 8.1. Introduction

Myocardial perfusion defects evaluated with the use of radiopharmaceuticals and induced by stress are well established as a technique with diagnostic and prognostic capability for the identification of flow-limiting coronary diseases. In MPS, interpretation has mainly been qualitative, semiquantitative, and quantitative, assessing regional perfusion in relative terms.^95,102,121,192-196^

### 8.2. Basic Principles of Positron Emission and Main Indications

PET consists of a specific method in nuclear medicine. It is different from the widely used gamma camera or Anger camera employed in MPS for the technique commonly known as SPECT. PET, differently from SPECT, uses emitters of positrons, particles similar to electrons (except for the fact that they have a positive electric charge), with very short half-lives. The principle of PET consists of the detection of 2 photons (gamma rays) that are emitted in diametrically opposite directions to occasion annihilation of the positron upon encountering an electron in the periphery of the atom. This detection occurs through a series of crystals arranged throughout the 360 degrees of a ring-shaped detector surrounding the patient. The detection of the 2 photons emitted in diametrically opposite directions, at exactly 180 degrees, with an existing coincidence circuit in the PET equipment, making it different from SPECT, which uses single photons.^197^

Since PET cameras have incorporated electronic collimation, mechanical collimators made of lead have not been made necessary, allowing for greater sensitivity than in SPECT systems. The sensitivity of current 3D PET systems is 5 times greater than of the older 2D PET ones. On the other hand, there is more attenuation in PET studies than in SPECT, making attenuation correction necessary to the reconstruction of PET images. The most current systems, which have a resource known as time of flight (TOF), are based on the speed of light to localize the annihilation event in a much smaller directional ray than in conventional PET cameras, resulting in increased spatial resolution.

This method has already been established as the standard for assessing myocardial viability (*See the Myocardial Viability section*), with the use of a glucose analogue labeled with fluorine-18 (^18^F-FDG), a technique which, although it is not widely used in Brazilian clinical practice, is widely viable in most nuclear medicine centers where PET is available in Brazil. Its use for the assessment of myocardial perfusion is not necessarily a new technique, as it dates back to more than 30 years ago and has since been evolving.^198-200^ Nonetheless, its clinical use has remained restricted for many years owing to its methodological complexity, high operational costs, and low availability of devices and tracers. Recent technological advances have reduced the costs, and its increasingly frequent use in oncology has resulted in increased equipment availability. Nowadays, non-invasive estimation of absolute coronary flow and flow reserve with this technique has become possible and has been validated.^197^ The scenario is contrary in Brazil, however. In spite of a small amount of experience using research protocols, this method is not available in clinical practice. Even though PET cameras are distributed throughout the country, there is a lack of other radiotracers for the modality as well as a lack of economic viability.

### 8.3. Radioactive Tracers for Use in Positron Emission Tomography

Different available radiotracers make it possible to identify vasoactive, metabolic, or neurological processes that are present in diverse cardiomyopathies and atherosclerosis, on the molecular level. The images acquired allow for evaluation of the cardiovascular system on different levels, including: perfusion,^201-203^ metabolism,^204-207^ sympathetic innervation,^208,209^ and inflammation;^210-212^ depending on the tracer utilized. Myocardial perfusion studies using PET may be performed using different tracers, each of which possesses specific characteristics, advantages, and disadvantages (Table 24).

**Table 24:** Main characteristics of perfusion myocardial radiotracers labeled with positron emitters

	Rubidium-82 (^82^Rb)	Ammonia labeled with Nitrogen-13 (^13^NH_3_)	Water labeled with Oxygen-15 (^15^O-H_2_O)	^18^F-Flurpiridaz
Physical half-life	1.27 min	9.97 min	2.04 min	110 min
Extraction fraction (flow)	40% to 70%	94% to 98%	95% to 100%	> 90%
Means of production	82-strontium/rubidium (^82^Sr/Rb) generator	Cyclotron	Cyclotron	Cyclotron
Advantages	• Commercially available in the form of a generator • Capable of evaluating flow quantitatively • Short half-life for quick tests • Low radiation	• High contrast resolution • Capable of evaluating flow quantitatively • Potential for use with exercise	• Ideal for quantification of flow • Short half-life for quick tests • Low radiation	• High contrast resolution • Capable of evaluating flow quantitatively • Potential for use with exercise • May be distributed by central production
Disadvantages	• Short half-life does not allow for exercise • Lower resolution	• Requires a local cyclotron • Heterogeneity of distribution	• Requires a local cyclotron • Short half-life does not allow for exercise	• Not commercially available

Rubidium-82 (^82^Rb) and ammonia labeled with nitrogen-13 (^13^NH_3_) have been approved for clinical use by the Food and Drug Administration (FDA), and they are the most commonly used in the USA. On the other hand, water labeled with oxygen-15 (^15^O-H_2_O) has been used mainly for research in the USA, as it diffuses freely between blood and the myocardium, which makes it ideal for quantitative flow measures. In South America and Brazil, associated costs, especially with tracers, have limited their use. Initial experience with myocardial perfusion using PET and ^82^Rb have been conducted at the Heart Institute of São Paulo (InCor, acronym in Portuguese).^213^ As ^82^Rb is the only one of these tracers produced in a generator system, from strontium-82, it has an advantage in relation to the others whose production depends on a cyclotron. These generators may be transported, and, specifically in case of Brazil, they may be imported especially for this purpose. Their short physical half-life of 76 seconds constitutes another favorable aspect, given that it implies very low dosimetry for the patient, with estimated exposure lower than 2 milliSieverts (mSv), in a stress and resting protocol, including tomography for attenuation correction. On the other hand, stress with exercise becomes unviable, considering the elevated costs, making it possible only in high-volume centers that perform around 40 exams weekly.^214^ Payment tables for medical procedures in Brazil, to date, restrict the payment of PET for oncological indications, which complicates its use for cardiology. The use of ^13^NH_3_ requires a cyclotron, the installation of which has extremely high costs, but it provides high contrast images, due to its high first-pass extraction fraction. It offers good accuracy for absolute measure of myocardial blood flow (MBF), and its relatively long half-life of approximately 10 minutes, makes physical exercise viable.^215^

^18^F-flurpiridaz is a new tracer. Although its production requires a cyclotron, its labeling with fluoride-18 makes it possible to utilize the production and distribution systems that are already widely available for oncological use of PET. Due to its relatively long half-life of 110 minutes, it is appropriate for associated use with an exercise testing, and its high first-pass extraction fraction also makes it ideal for flow quantification. Its use is currently being evaluated in a phase-III study.^216-220^

### 8.4. Use of PET for Assessment of Myocardial Ischemia

PET has advantages over conventional SPECT, including higher spatial resolution and contrast rate, higher sensitivity than the tracers classically used in MPS (thallium-201 and technetium-99m-labeled radiopharmaceuticals), and higher specificity, considering the attenuation correction system based on coupled CT, which results in better capability to differentiate true perfusion defects from attenuation artifacts.^201,221-223^ These advantages are notably applied to some special populations, such as obese patients and women with voluminous breasts, in whom gamma ray attenuation in soft tissue may be a factor of greater importance to the final quality of cardiac images.

The objective of evaluating myocardial perfusion via PET is to detect physiologically significant coronary stenoses, aiding clinical management of patients with known or suspected CAD and patients who, although they have no known diseases, possess risk factors, in order to evaluate atherosclerosis progression. Other objectives include determining the cause of ischemic symptoms to recommend clinical treatment or revascularization, estimating potential for future adverse events, and improving patient survival. One of its strengths is that it is the non-invasive modality of choice for accurately quantifying MBF. It allows for quantification in absolute terms of ml.min per gram of myocardium in stress and resting phases. The ratio between the 2 flows is known as the myocardial flow reserve (MFR), a valuable parameter that makes it possible to overcome one of the currently existing limitations to conventional perfusion imaging with SPECT when evaluating patients with multivessel CAD.

Results of invasive studies (FAME-1 and FAME-2) that analyzed FFR demonstrated its value in evaluating functional significance of single-vessel stenoses.^224,225^ Some studies have provided evidence of a correlation between regional MFR and FFR measured invasively without comparing them directly, however.^113,226^

**Figure 17:**
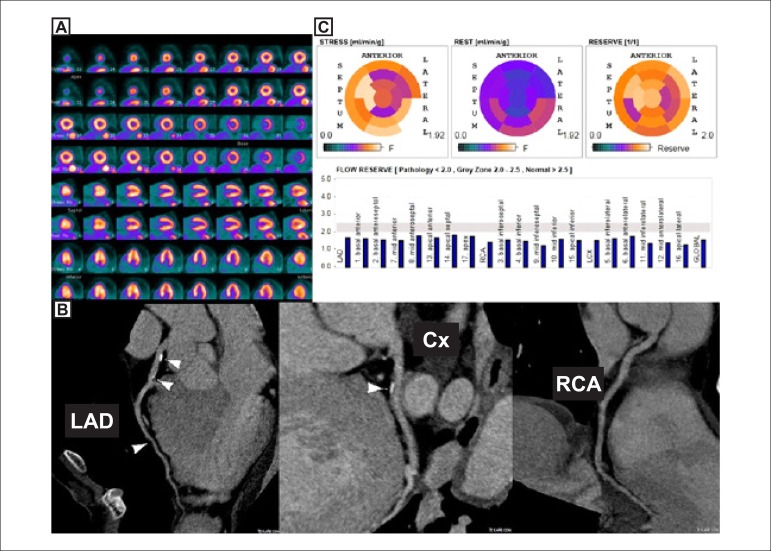
A 53-year old patient, of pardo race, with chronic renal insufficiency, undergoing hemodialysis, and left ventricular hypertrophy. Pre-renal transplant evaluation. Counterclockwise: A) PET perfusion with rubidium-82 with no segmentary defects in uptake between different walls of the left ventricle. B) Coronary tomography with evidence of parietal calcium in the anterior descending and circumflex arteries, but no obstructive lesions. C) Quantification of myocardial blood flow and flow reserve, widely reduced throughout all coronary territories (In the bar graph, coronary flow values < 2.0 ml.min-1.gram-1 of myocardium are considered abnormal; values between 2.0 and 2.5 ml.min-1.gram-1 are considered in the gray zone; and values > 2.5 ml.min-1.gram-1 are considered normal), notwithstanding the absence of obstructive epicardial disease, are indicative of microvascular disease. LAD: left anterior descending; Cx: circunflex; RCA: rigth coronary artery.

Quantitative PET measures of MBF in absolute terms represent a paradigm change in the evaluation and management of patients with CAD, with a disassociation from the anatomical gold standard of coronary cineangiography, which had previously been established for decades, and a return to functional assessment. These measures additionally make it possible to expand the use of perfusion imaging within the current scenario, with the aim of detecting flow-limiting epicardial lesions, for earlier stages of atherosclerosis, microvascular dysfunction (Figure 17), and evaluation of balanced flow reductions in triple-vessel disease. They also offer an opportunity to monitor responses to changes in lifestyle or risk factors and therapeutic interventions.^227,228^

Source - INCOR - FMUSP - SP.

Two recent meta-analyses evaluating methodology have indicated that PET has superior accuracy in comparison with SPECT. The first meta-analysis compared PET with SPECT synchronized with ECG and associated with attenuation correction. In analysis with a ROC curve, the area under the curve for PET and SPECT was, respectively, 0.95 and 0.90 (p < 0.0001), showing a small superiority for PET. The second meta-analysis indicated ^82^Rb as the most used tracer, resulting in higher sensitivity for PET. Specificity, on the other hand, although superior, was not statistically significant.^222^

Regarding prognosis, similarly to SPECT, for which data are abundant, robust, and well established, normal myocardial perfusion with PET is indicative of good prognosis, with cardiac events varying between 0.09% and 0.9% during 1 year of follow-up, depending on the population analyzed. On the other hand, adverse events increase with the extent of perfusion defects on PET. A recently published register including more than 7,000 patients demonstrated that the hazard ratio of cardiac death increased with every 10% increment of extent of perfusion defects, classified as mild, moderate, and severe, respectively, hazard ratio: 2.3 (95% CI: 1.4-3.8; p = 0.001); hazard ratio: 4.2 (95% CI: 2.3-7.5; p < 0.001), and hazard ratio: 4.9 (95% CI: 2.5-9.6; p < 0.0001), in relation to a normal exam.^221^

### 8.5. Patient Preparation, Types of Stress, and Dosimetry

Preparations include a 6-hour water-only fast. Patients should avoid caffeine and foods or medications containing xanthines (theophylline, theobromine) for at least 24 hours. Generally speaking, stress protocols are generic for all types of perfusion agents, bearing similarities to those of MPS with SPECT, with specific differences in accordance with acquisition protocols.

Current dosimetry for studies with rubidium-82 (^82^Rb) in adults, considering maximum administered activity per 60-mCi dose, may vary from 1.1 to 3.5 mSv of total effective dose. With the current advances in instrumentation of PET cameras, studies with good diagnostic quality may be acquired with injected activities that vary from 20 to 40 mCi per resting and stress dose, resulting in even lower exposure. In studies with ammonia labeled with nitrogen-13 (^13^NH_3_), the habitual activity is 10 to 20 mCi per dose (which corresponds to 1.48 mSv per dose). Doses of up to 25 to 30 mCi may be used in patients with high body mass index (BMI), with relatively lower dosimetry as a function of its shorter half-life and the low energy of its positron.

The evolution of imaging systems has allowed for the development of PET/CT capable of performing hybrid imaging, or be it, using CT not only for attenuation correction but also for quantification of CS and acquisition of coronary angio-CT, in addition to allowing for the fusion of these images, facilitating the integration of anatomical and functional information. Another great advance is that of PET systems incorporated to PET/MR. This new hybrid imaging modality has enormous potential for structural-functional evaluation, tissue characterization, and reduced exposure to radiation.^229-231^ This, thus, amplifies the possibility of developing new studies, with the aim of expanding data on clinical applications and diagnostic and prognostic benefits of PET in studies of more diverse cardiovascular conditions.

**Figure 18:**
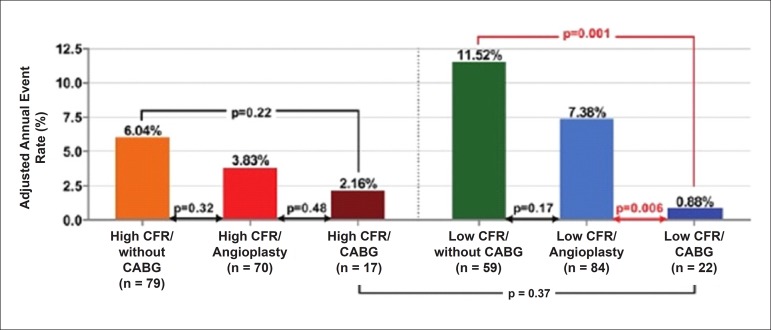
Coronary flow reserve (CFR). Left: In patients with high CRF, there were no statistically significant differences in adjusted rates of annual events, notwithstanding apparent reduction observed in patients who underwent angioplasty or coronary artery bypass graft (CABG). Right: In patients with low CRF, both procedures showed significant benefits in reducing events.

Source: Adapted from Taqueti VR et al.^232^

Objective measures of coronary flow reserve will certainly be able to be extended to the MPS-SPECT method, providing evidence of nuclear medicine’s ability to carry out quantifications of MBF and allowing for additional parameters for evaluating perfusion, as well as myocardial reserve, with a resulting impact on clinical management of patients, which will objectively orient decisions about revascularization (Figure 18).^232^

Provided that availability barriers are overcome and costs of both imaging systems and tracers are reduced, especially in developing countries such as Brazil, the growing application of this new methodology has a promising outlook in cardiology.

## 9. Integrating Diagnostic Modalities in Cardiology - Tutorial Cases

### 9.1. Introduction

Technological evolution has facilitated the development of excellent tools for both establishing diagnosis and estimating prognosis of CAD. These advances allow us to evaluate different aspects of anatomy and physiology of the heart non-invasively, with great accuracy. Most importantly, today we are able to rely on methods which help establish the best course of treatment in the most diverse clinical situations of patients with suspected or known diseases, whether they are symptomatic or asymptomatic. This wide range of alternatives presents an additional challenge to doctors, namely, that of defining the best strategy and the most rational complementary sequence of evaluation possible, regarding use of resources for diverse clinical situations, guaranteeing not only the highest accuracy in evaluation, but also the best benefits, considering healthcare costs. Doctors generally should seek to orient initial investigation with the aim of using the lowest number of diagnostic exams for an effective evaluation. However, in this era of multimodalities, it has become necessary to perform more than 1 exam in order to make the best therapeutic decision. Combined assessment of different phenomena of the heart is often necessary, for instance, to define physiological repercussions of an anatomical lesion.

Two questions linked to the bases of medical semiology and to the essence of medicine ask, **“Who is the patient?”** and **“What information is the doctor looking for?”** In this approach, the application of good techniques, such as anamnesis and complete physical examination, has become clear in clinical medicine, enabling doctors to formulate their initial patient profiles and to establish the most probable diagnostic hypotheses. Joint estimation of the pre-test probability of the disease^233^ and knowledge regarding the accuracy of a test to determine post-test probability of a true or false result (Bayes’ Theorem) are implicit and no less important. The application of this basic principle, associated with knowledge regarding what different diagnostic tools may offer, allows for the elaboration of better investigation strategies. Confirming or excluding the presence of CAD from the anatomical point of view or, alternatively, investigating the physiological repercussions of myocardial ischemia via stress tests have distinct implications for patient management. Whether to look for one response or another will depend on the patient at hand and the question the doctor wishes to answer.

### 9.2. Integrating Physiology (Exercise Testing and Nuclear Cardiology) and Anatomy (Calcium Score and Coronary Angiotomography)

Exercise testing (ET), also known as ergometric test, stress or exercise tests, showing evidence of good performance (high workload) with normal results and MPS showing absence of ischemia do not represent absence of CAD indeed . In the presence of CAD, however, these findings are associated with better prognosis in relation to patients with ischemia, given that their use is extremely useful for risk stratification of patients with or without this disease. On the other hand, it is important to know that methodologies based on the anatomy of coronary arteries, such as coronary angio-CT, may also stratify risk, but the presence of atherosclerosis detected by this modality does not necessarily imply poor prognosis or, much less, mean that the patient will necessarily benefit from myocardial revascularization procedures. It may merely represent that prognosis is worse that that of an individual without atherosclerosis. It is, thus, worth reaffirming that it is necessary for doctors to possess global knowledge of their patients and also to delineate clearly the investigation strategy for the question they are seeking to answer. Basic knowledge regarding advantages and disadvantages of available procedures are implicit, making the absolute most of the technological evolutions that have occurred in recent years. Initially speaking, all modalities which have been covered may be used for diagnosis and prognosis. It is, however, evident that they all have strengths and limitations, which are not necessarily uniform for all patients; or be it, there are determined patient characteristics which may make one test superior or inferior to another (Table 25).

**Table 25:** Main advantages and disadvantages of exercise testing (ET), myocardial perfusion scintigraphy (MPS), and coronary angiotomography (angio-CT) for assessment of coronary artery disease (CAD)

	Advantages	Disadvantages
ET	• Widely available • Relatively low complexity • Relatively low cost • Does not involve radiation	• Requires ability to exercise • ECG may be uninterpretable • Limited accuracy • Does not detect initial CAD
MPS	• Localizes and quantifies ischemia • Evaluates perfusion and LV function associated with exercise • Evaluates ischemia in patients unable to exercise • Makes it possible to monitor treatment	• Technological complexity • Uses radiation • Attenuation artifacts • Does not detect initial CAD
Angio-CT	• Excludes CAD with great accuracy • Detects CAD in its initial phase • Allows for anatomical evaluation (e.g., anomalous coronaries) • Quick exam	• May overestimate obstructions • Limited use for known CAD • Limited for physiological aspects • Uses radiation

ECG: electrocardiogram; LV: left ventricle. Source: Adapted from Vitola JV.^234^

**Figure 19:**
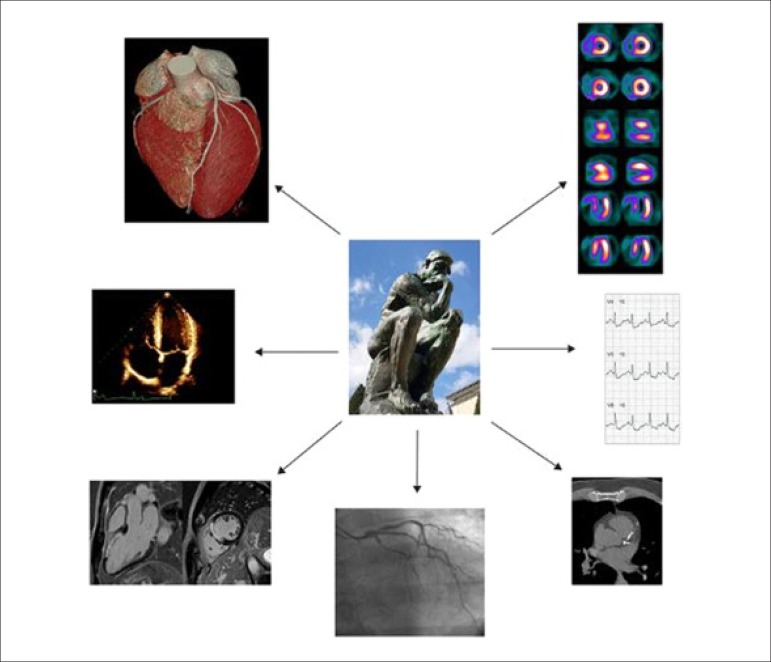
The importance of integrating information about different diagnostic modalities in an era of multimodalities. Generally speaking, the diagnostic modalities most used in Brazil are: exercise testing, echocardiogram, myocardial perfusion scintigraphy, angiotomography to evaluate coronary anatomy and calcium score, cardiac magnetic resonance, and, finally, invasive coronary cineangiography (cardiac catheterization). In this scenario, the doctor is central to establishing the best strategy and should ask the following questions: (1) What is my patient’s clinical profile? (2) What information am I looking for regarding the clinical hypotheses I have raised? (3) What test will provide this information? (4) Will I need further information to make a decision and manage this patient?

Source: Vitola JV.^234^

Once the doctor has answered the 2 initial questions, **“Who is the patient?”** and **“What is the main diagnostic hypothesis?”** he or she needs to answer the third question: “**What is the most appropriate test for this patient and for the question I want to answer?”** To answer this question, it is essential to know the main advantages and disadvantages of the exams, integrating the results of Bayes’ theorem and, thus, defining post-test probability. It is, moreover, necessary to identify when continuous or complementary analysis will be required in order to obtain additional information for better patient management (Figure 19).

Of the techniques that have been covered, angio-CT is the most recent, showing great technological evolution, notably over the past 10 years. Data from important clinical studies have consolidated and recognized the value of this modality, as well as how to integrate it with other available tools. Before the advent of angio-CT, studies of patient anatomy presented greater difficulties, as it was mainly obtained via cardiac catheterization, with all the limitations, complications, and costs associated with invasive interventions. The development of a non-invasive, relatively simple and quick imaging technique has, in recent years, made it possible to recover the role of anatomical evaluation of the heart as a diagnostic and prognostic tool, thus integrating angio-CT into the multimodality scenario. Tests that allow for evaluation of cardiac physiology, such as ET and nuclear cardiology with MPS, have been routinely utilized for many decades, and, as they are not invasive, they have been employed for a large number of patients with suspected or known CAD. A great deal has been learned about these tools’ capabilities for diagnosis and, especially, for establishing prognosis and defining better courses of treatment.

Since the beginning of ET, initially in the 1950’s, followed by MPS in the 1970’s, information obtained has emphasized the great value of physiology, especially for evaluated coronary reserve flow, with highly consistent data for stratifying risk of cardiac death in patients with known or suspected CAD. Different physiological variables assist in characterizing patients as low-, intermediate-, or high-risk. The results of these tests, however, are not always in agreement, when comparing physiological tests to each another (ET with MPS) or to anatomical tests (angio-CT and catheterization). Potential “disagreements” and situations that may generate doubts are more common when comparing anatomy and physiology, especially at this moment, with the expanded use of angio-CT.

The scope of this text is not to revise details in relation to the variables involved, but rather to integrate information obtained from different available non-invasive tools, especially the interrelations between MPS, angio-CT, and ET. Summarily, the main variables of **ET** that represent *high risk* are: low functional capacity, greater magnitude of ST depression, occurrence in multiple leads, descending ST segment, ST-segment elevation in leads without Q waves, depressed chronotropic response, drop in blood pressure during stress, the presence of complex ventricular arrhythmia, manifestations of angina during low workloads, among others. In MPS, the *principal markers of severity* are: extensive perfusion defects with severe intensity, especially transient defects (transient reduced uptake) in more than 1 territory and mixed fibrosis patterns associated with ischemia (persistent reduced uptake associated with transient reduced uptake), stress-induced LV dilation, tracer uptake in the RV and the lungs, low LVEF, and LV with or without transient dilation associated with stress.

Conversely, aspects associated with *low risk on the ET* are represented by high functional capacity, absence of important ST-segment abnormalities or stress angina, good hemodynamic response with appropriate increase in HR and blood pressure, and absence of complex ventricular arrhythmias. In relation to MPS, markers of *good prognosis* are associated with normal myocardial perfusion and preserved LV ventricular function. Most of the time, especially in the most severe cases, diagnostic modalities are in agreement, or be it, a patient with high-risk ET findings will, likely, show significant MPS defects, corresponding to coronary anatomy compatible with advanced CAD. There are, however, different scenarios in which disagreeing results present challenges to better patient management.

**Figure 20:**
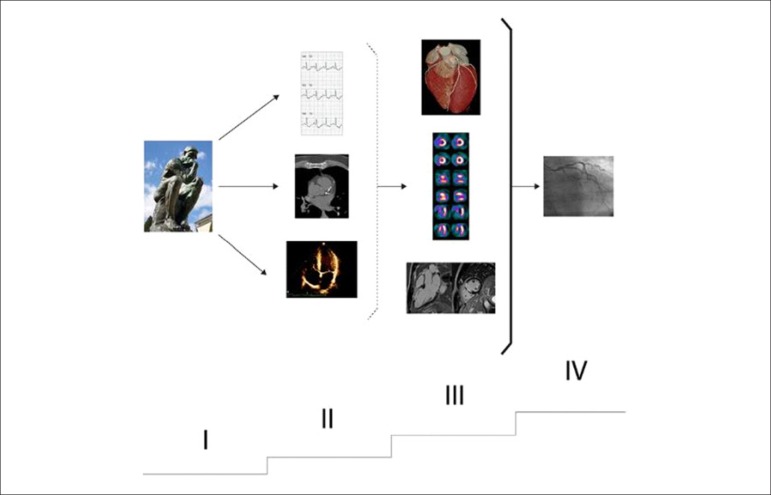
Concept of a rational strategy for evaluating and integrating modalities in a logical sequence of investigation of stable patients. It begins with the best rationalization for formulating diagnostic hypotheses (I), going on to the most basic tests (II), such as ECG/ET, ECHO, CS, continuing, as necessary, to more advanced non-invasive imaging methods (III), such as angio-CT, MPS, and CMR. The non-invasive tests, whether basic or advanced, should serve as “filters” for invasive testing, i.e. cardiac catheterization (IV), which should serve in planning advanced treatment only in patients under consideration for myocardial revascularization.^234^

The cases subsequently exposed are intended to integrate clinical data with the use of multimodalities, extending discussions within the medical decision-making process for understanding, interpreting, and suggesting conduct for dealing with agreements and, especially, disagreements. Figure 20 illustrates a concept in which the doctor begins evaluation using medical procedures of anamnesis and physical examination during the *initial phase (I)*, formulating the main diagnostic hypotheses, which are fundamental aspects for defining the best investigation strategy. Subsequently, depending on the questions formulated, relatively simple or more *basic* diagnostic tests are obtained *(II)*, such as: resting ECG, ET, resting ECHO and, eventually, CS. Subsequently, in accordance with the need for additional information and depending on the diagnostic hypotheses considered, the doctor may consider the application of more *advanced* non-invasive imaging techniques *(III)*, such as angio-CT, MPS, and, potentially, cardiac magnetic resonance. Applying scientific knowledge regarding diagnostic and prognostic value, to both basic tests and more advanced non-invasive imaging methods, it is possible to establish filters for selecting patients who will really require invasive testing, such as *cardiac catheterization (IV)*, notably with the aim of planning for myocardial revascularization.

### 9.3. Practical Examples of Integration of Modalities

#### 1. Patient with abnormal ET, Duke score characterizing intermediate risk, and normal MPS

**Figure 21:**
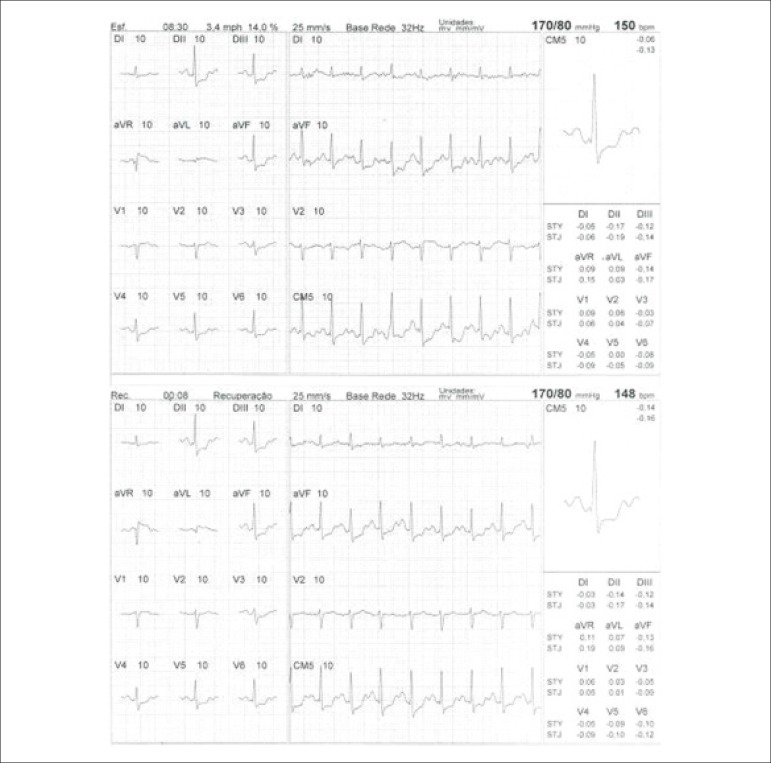
Case 1 - Electrocardiogram tracing during peak stress and initial recovery with alterations (explained in the text).

**Figure 22:**
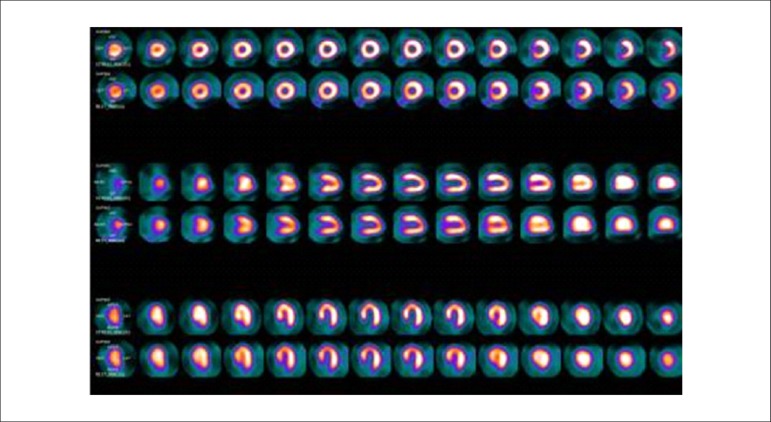
Case 1 - Myocardial perfusion scintigraphy within normality. Images acquired with dedicated cardiac equipment (gamma camera), equipped with solid cadmium-zinc-tellurium detectors.

**Clinical history:** female, age 50, with hypertension, dyslipidemia, atypical symptoms, and borderline ET. Referred for MPS (Figures 21 and 22).

**Findings:** The patient exercised for 8.5 minutes on the Bruce protocol, with satisfactory HR and blood pressure responses, reproducing the ET findings that motivated referral for MPS, with ST-segment depression varying from 1 to 1.5 mm after 80 mm on the J point, with aspect varying from slow ascending to horizontal, in multiple leads (Figure 21). She denied having precordial pain, but there was a manifestation of cervical and mandibular discomfort. Calculation of the *Duke Score*, $\mathrm{DS}=\mathrm{Time}\;\mathrm{in}\;\min.-\left(5\times \mathrm{ST}\right)-\left(4\times \mathrm{Angina}\;\mathrm{Index}\right)$, resulted in intermediate risk, both considering the symptom as angina $\left[+8.5-\left(5\times 1.5\right)-\left(4\times 1\right)\right]=\left.-3\right]$ and not considering it as angina $\left[+8.5-\left(5\times 1.5\right)-4\times 0\right]=\left.+1\right]$. MPS was within normality.

**Comments:** This is one of the most common situations in nuclear cardiology laboratories. The first questions to be formulated refer to the risk defined by the ET. Abnormal responses may characterize low, intermediate, or high risks. For MPS, the best indication is for intermediate risk, which was the case with this patient. It should ideally be associated with physical exercise instead of pharmacological alternatives; when this is normal, the patient is stratified as low risk and, in most cases, the exam will indicate a probability of death lower than 1% per year, implying conservative medical management. At this moment, investigation may cease, based on the conclusion that the patient’s symptoms are not related to significant myocardial ischemia and that he or she requires prevention with the objective of controlling hypertension and dyslipidemia. Other findings in clinical practice include patients with functional capacity similar to the case described but with higher magnitude of ST-segment depression, resulting in a high-risk Duke score. Due to the absence of angina during stress and good functional capacity, however, the doctor may suspect that the calculation is overestimating the risk via ET. Such findings may be observed more frequently in patients with hypertension, possibly related to myocardial hypertrophy. In Brazil, Vitola et al. studied patients with high-risk Duke scores and MPS results, finding perfusion abnormalities in 70% of these individuals.^235^ However, the other 30% showed normal MPS, and it was demonstrated that these patients had excellent prognosis. Thus, in specific cases, even in the presence of high risks characterized by the same score, the application of multimodalities, such as the association of physical stress with non-invasive MPS imaging, are appropriate before proceeding to investigation via catheterization. Furthermore, it may also be possible to utilize angio-CT in some cases, considering its high NPV, with the aim of clarifying diagnosis and excluding important CAD, especially in young patients, where the probability of a “false-positive” result is higher.

#### 2. Patient with normal ET and abnormal MPS

**Clinical history:** male, age 36, long-standing DM, insulin dependent, obese and hypertensive. Atypical symptoms, notably related to fatigue during stress. Referred for MPS to investigate ischemia, following ET which was normal but which had low sensitivity owing to electric axis deviation to the left, suggestive of left anterior fascicular block (LAFB) on resting ECG.

**Figure 23:**
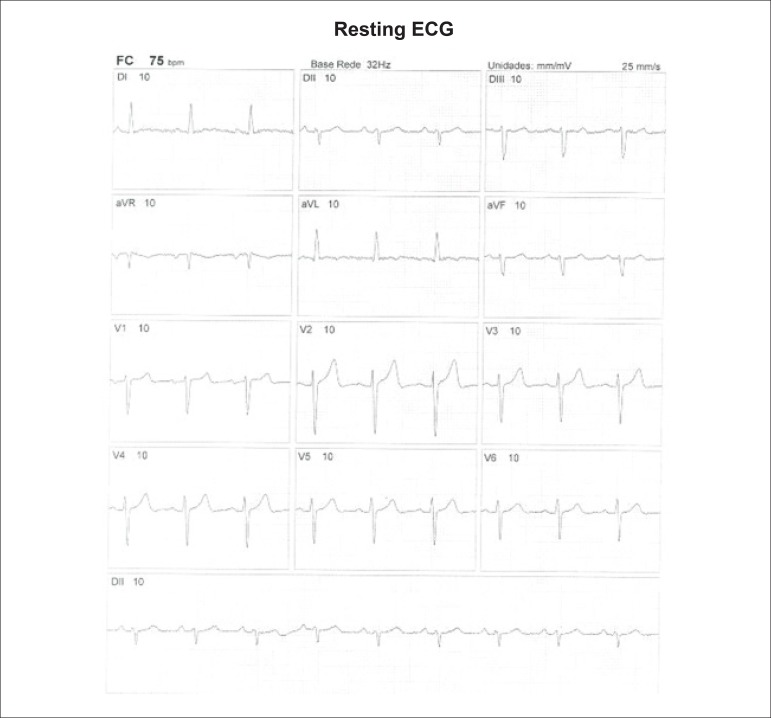
Case 2 - Resting electrocardiogram suggestive of left anterior fascicular block.

**Figure 24:**
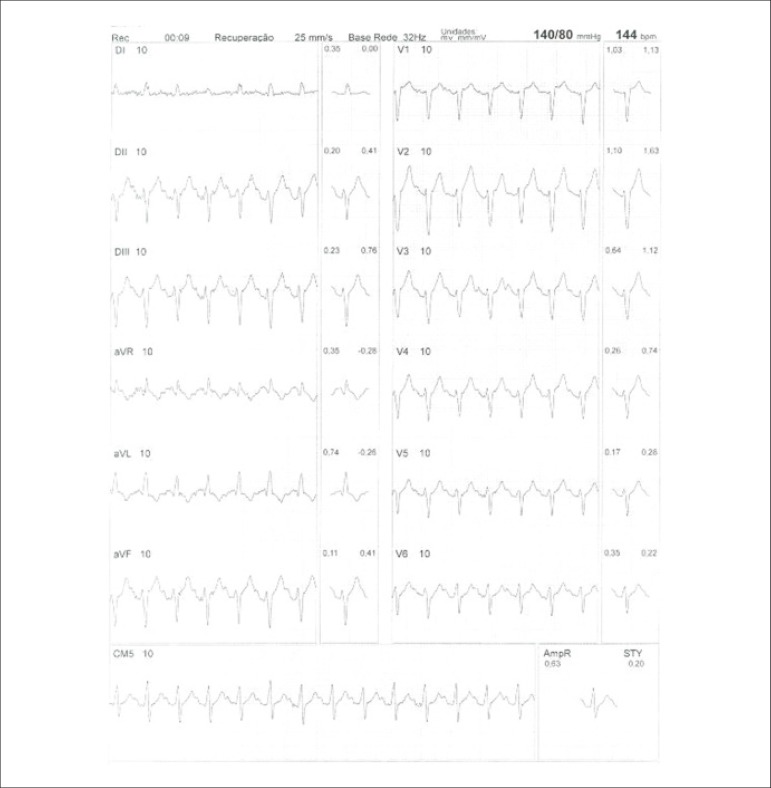
Case 2 - Electrocardiogram obtained during immediate recovery, representing peak effort, heart rate of 144 bpm, with total workload performed considered satisfactory, in addition to the absence of ischemic ST-segment alterations.

**Figure 25:**
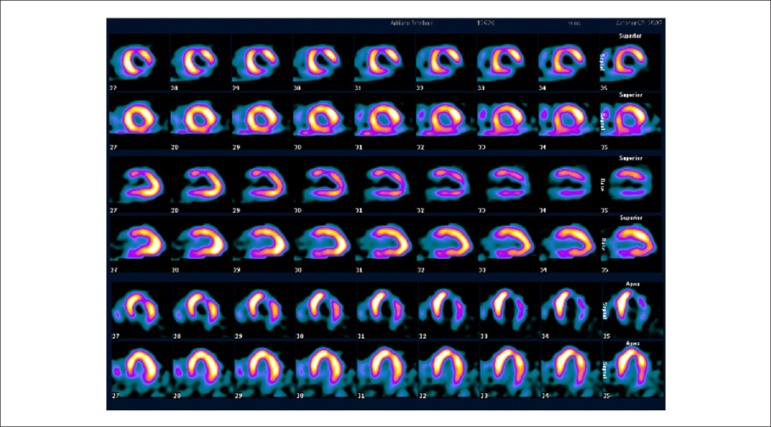
Case 2 - Myocardial perfusion scintigraphy showing important myocardial perfusion abnormalities, with multivessel ischemia and transient left ventricular cavity dilatation, representing high-risk indicators. Images acquired with dedicated cardiac equipment (gamma camera), equipped with conventional sodium iodide crystals.

**Findings:** Resting ECG characterized LAFB (Figure 23). Time in the Bruce protocol was 10 minutes, with neither angina nor ST-segment alterations (Figure 24). Perfusion images showed transient reduced uptake, with large extension and moderate to severe intensity, suggestive of ischemia, involving predominantly the inferolateral, lateral, anterior, and anteroseptal walls of the LV, extending to the apex (Figure 25). Observe how the LV cavity dilate after physical exercise, with the appearance of diffuse hypokinesis and a drop in LVEF from 55% to 45% when comparing both stages.

**Comments:** Given a normal ET, with a high workload or good performance, with neither angina nor ST-segment alterations, patients are generally considered to have low post-test risk, but this is not always the case, as can be seen here. Nor does a normal ET represent the absence of CAD, as potentially shown by the presence of calcium in the coronary arteries on angio-CT or by ischemia detected by a more sensitive technique, such as MPS. In accordance with the evolution of medical knowledge in this era of multimodalities, restratification, even of patients with low risk on exercise testing, has become possible, as an exception. These possibilities should be considered more frequently in patients with family history of early CAD, DM, or multiple combined risk factors, and especially in those with high clinical risk (Framingham score) or LAFB on resting ECG. The case presented exemplifies precisely this scenario of a clinically high-risk patient (multiple risk factors, including DM), with LAFB on resting ECG and low risks on ET, who was restratified to a higher level of risk via perfusion imaging, due to the presence of important ischemia and LV dysfunction during stress, which are high-risk indicators. In this condition, when these tests are in disagreement, in a young, symptomatic patient (probable ischemic equivalent) with high clinical risk confirmed on an imaging exam, referral for coronary cineangiography is supported as part of medical management.

#### 3. Patient in pre-operative evaluation for non-cardiac surgery, with mild abnormalities on MPS, high calcium score, and non-obstructive CAD

**Clinical history:** male, age 65, hypertensive, obese (BMI = 45), stroke 5 years prior, asymptomatic, in pre-operative evaluation for cholecystectomy and bariatric surgery. Interpretable resting ECG, unable to exercise. Referred for MPS with dipyridamole as initial investigation exam.

**Figure 26:**
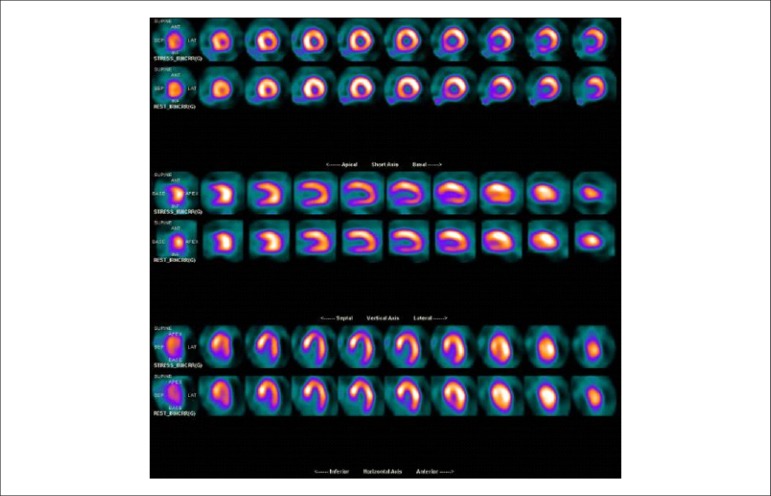
Case 3 - Myocardial perfusion scintigraphy showing mild alterations in myocardial perfusion, with analysis limited by significant obesity (grade III). Images acquired with dedicated cardiac equipment (gamma camera), equipped with conventional sodium iodide crystals.

**Figure 27:**
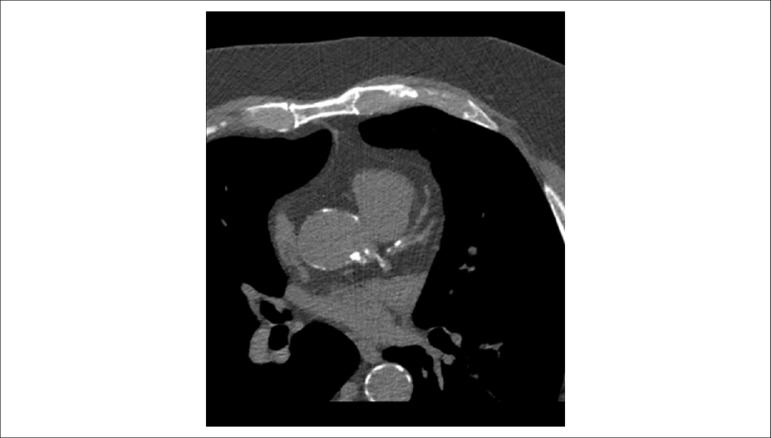
Case 3 - Angio-CT showing vascular calcifications involving coronary arteries and ascending and descending aorta.

**Findings:** ECG tracings show no modification during and after intravenous administration of dipyridamole. Perfusion images reveal mild defects (small extension) in radiopharmaceutical uptake in the inferior and inferolateral/lateral walls and in the LV apex (the latter being transient), with preserved LV function (Figure 26). Considering the 2 protocol series of image acquisition, resting and under pharmacological stimulation, interpretation is limited due to the significant obesity. The finding may even represent an attenuation artifact. With the patient in an asymptomatic conditions, with the reported alterations in perfusion and preserved LV function, additional information is required before making the important decision of approving the patient for surgery, and anatomical evaluation via angio-CT is thus recommended. The findings indicate a CS of 1,621 measured by the Agatston score, corresponding to the 96% percentile, when compared to individuals of the same sex, age, and race.^236^ Moreover, there is evidence of non-obstructive lesions (< 30%) in all coronaries and an absence of significant obstructions > 50% (Figure 27).

**Comments:** In the presence of multiple risk factors, considering the patient’s age and stroke history, the probability of CAD is intermediate to high. Pre-operative risk stratification is necessary, and, although the resting ECG was interpretable, the patient was unable to perform exercise. MPS with dipyridamole is well indicated, given that normal results could lead to the patient being approved for surgery. On the other hand, faced with abnormalities in perfusion and/or function, with indicators of high risk, there is a sufficient base of evidence for indicating catheterization. In this case, however, the result showed normal LV function and mild alterations in perfusion, with the possible presence of artifacts or results of small vessel CAD (microcirculation) and/or endothelial dysfunction. The angio-CT findings indicated high CS compatible with a high atherosclerotic burden and poor long-term prognosis,^237^ which was not surprising given the profile of this patient whose coronary calcium (CC) was in the 96% percentile, meaning that 96% of individuals of the same age, sex, and race had lower coronary calcification indexes than the case described. Nonetheless, the contrasting anatomical evaluation revealed non-obstructive coronary lesions (< 30%), which reinforces the possibility that the patient might have small vessel CAD, which already bears physiological repercussions, implying more aggressive clinical management. The absence of significant obstructive lesions or high-risk anatomy serves as an additional filters for avoiding invasive examination (catheterization) and confirming that surgical risk would not be prohibitive. The most appropriate form of management for this patient, likewise, appears to gear toward aggressive preventative measures, with risk-factor control and follow up, in addition to medical treatment of CAD, which does not, however, require myocardial revascularization. Bariatric surgery itself may perhaps assist in controlling these risk factors.

#### 4. Patient with elevated CS normal MPS and ET

**Clinical history:** male, age 52, asymptomatic, diagnosed with DM 5 years prior, hypertensive and dyslipidemic. Calculated CS.

**Figure 28:**
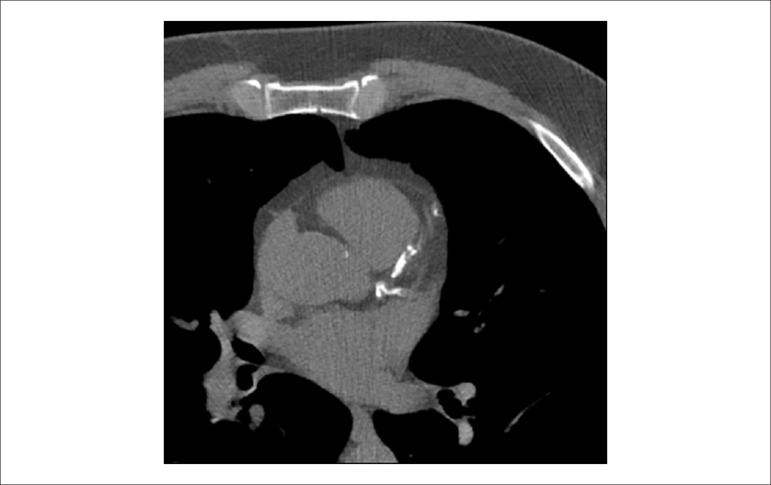
Case 4 - Angio-CT imaging shows elevated calcification index in coronary arteries.

**Figure 29:**
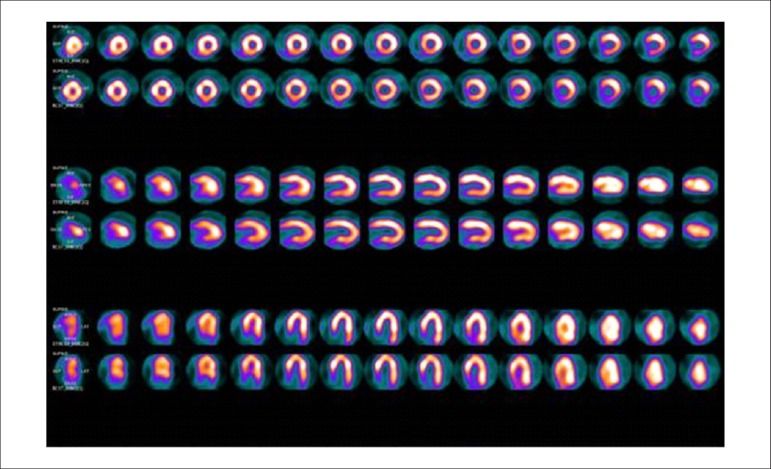
Case 4 - Myocardial perfusion scintigraphy within normality. Images acquired with dedicated cardiac equipment, equipped with solid cadmium-zinc-tellurium detectors.

**Findings:** CS resulted in a high Agatston score of 1,143, in the 99% percentile (Figure 28). MPS with physical exercise was indicated. Patient underwent stress in the Bruce protocol for 10 minutes, reaching HR of 158 bpm (94% of the recommended maximum HR), with no clinical, electrocardiographic, or hemodynamic alterations. MPS (with a CZT camera) showed homogenous radiopharmaceutical distribution in the LV walls (Figure 29), as well as normal LV systolic function.

**Comments:** This situation has occurred more frequently in clinical practice, to the extent that CS has gone on to be incorporated as a screening method for CAD and risk stratification in the subgroup of asymptomatic patients (DM and intermediate Framingham score). This disagreement between results is understandable given that the presence of atherosclerosis will not necessarily result in ischemia detected by functional methods. For instance, an ET may indicate low risk according to the Duke score in a patient who has performed only 5 minutes of exercise in the Bruce protocol but who showed neither ST alterations nor angina. It is intuitive to grasp that, in the presence of coronary disease (most cases with high CS), this does not represent exactly the same low risks as in a patient without CAD (absence of coronary calcification or zero CS). Regarding these facts, there is extensive literature on the prognostic value of CS, with long follow-up periods (> 15 years).^238^ In this manner, it is feasible to expect the group characterized as low-risk by the Duke score to be heterogeneous, and patients should thus be treated individually, considering the intensity of prevention. In the case demonstrated, as the patient has DM, there had already been an indication for statin use, with the very high CS (in the 99% percentile) reallocating the patient into an even higher risk within the group with DM. As part of data revision which has been occurring over the past 20 years, cases have been found which showed normal but which, nonetheless, presented coronary events during medium-term evolution, in a manner similar to the discrepancies recently observed between ET and CT. Likewise, a recent study by Chang et al.^239^ observed the same discrepancies in patients with low-risk Duke scores from ET and CS > 400. They evaluated 946 patients with the Framingham score, classifying the majority as intermediate-risk (estimated 11.1% average for events over 10 years) and, basically, asymptomatic, as evaluated by ET and CS. The average Duke score was 8.4, categorized as low-risk (≥ 5). Stress tests were positive or altered in 12.3% of patients, while CS > 100 were found in 54.2% of patients. MPS was abnormal in 10.9% of the same population. It was demonstrated that CS restratified risk for patients with low-risk Duke scores, identifying individuals with atherosclerosis and higher propensity for events. Furthermore, a current register known as CONFIRM has accumulated data that definitively suggest that, in the presence of non-obstructive CAD,^240^ evolution may be worse in patients without CAD. It has, thus, become evident that the anatomical technique with angio-CT is identifying coronary atherosclerosis earlier. This set of information definitively represents a change of paradigm in the medical decision-making process. In this situation where CAD is identified, medical management will be geared toward more aggressive prevention of modifiable risk factors and minute observation of possible symptoms that translate to disease instability. In the absence of ischemia, revascularization procedures should not be considered.

#### 5. Patient with high CS and abnormal MPS

**Clinical history**: female, age 68, asymptomatic, with intermediate-risk Framingham score. Performed CS for risk restratification.

**Images:** reproduced with permission of Vitola JV.^234^

**Figure 30:**
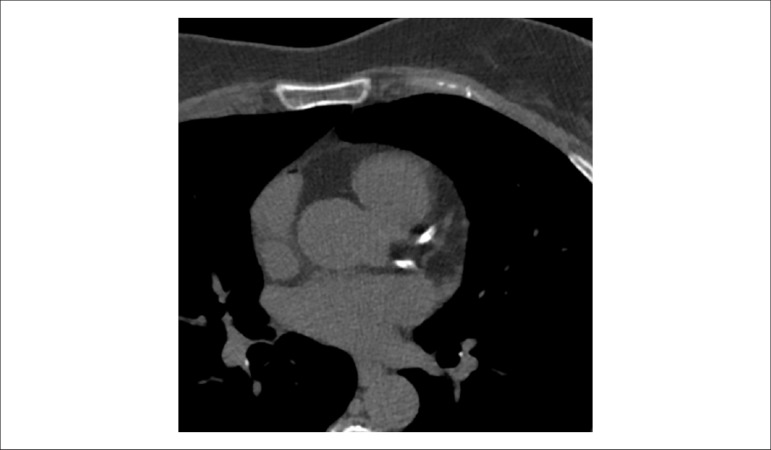
Case 5 - Angio-CT revealing severe calcification in coronary arteries.

**Figure 31:**
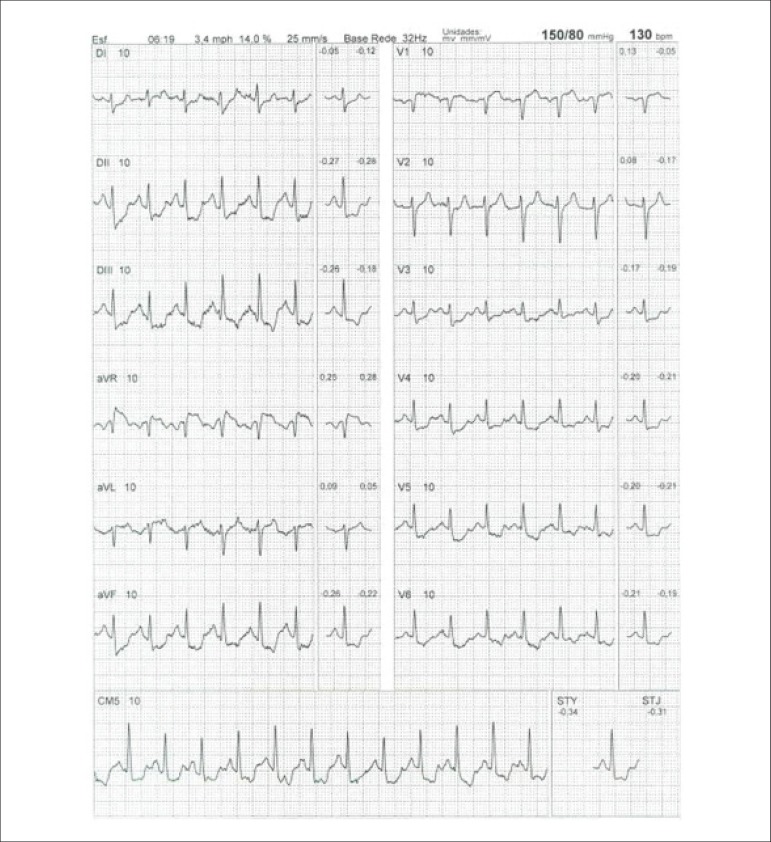
Case 5 - Electrocardiogram tracing demonstrating ischemic electrocardiographic alterations in multiple leads, with submaximal heart rate levels. Detailed explanation in the text.

**Figure 32:**
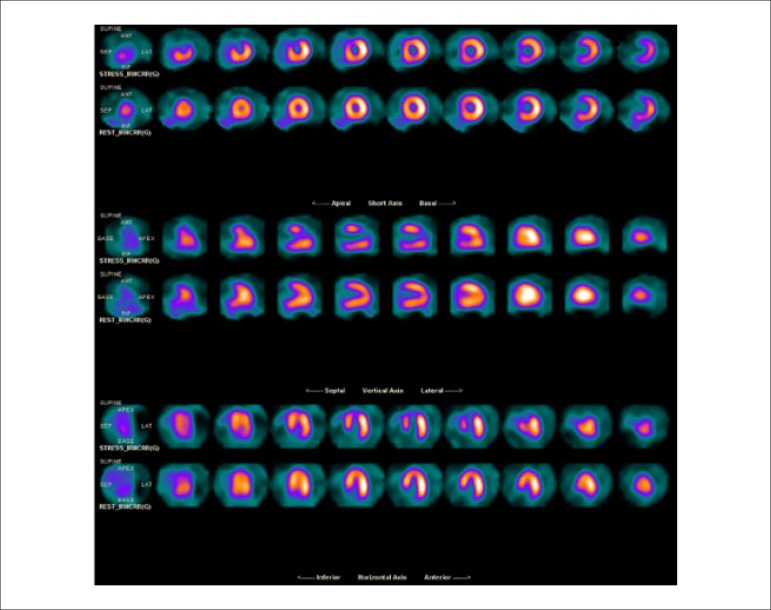
Case 5 - Myocardial perfusion scintigraphy demonstrating significant ischemia in the territory of the anterior descending artery. Images acquired with dedicated cardiac equipment (gamma camera), equipped with conventional sodium iodide crystals.

**Findings:** The resulting CS was high, at 1,282, according to the Agatston score, placing this patient in the 99% percentile (Figure 30). With this finding, functional evaluation was indicated, using MPS with MIBI-^99m^Tc associated with exercise. The patient exercised for 7.5 minutes in the Bruce protocol, showing ST-segment depression of up to 3 mm during peak stress, with a varying aspect which tended toward descending in multiple leads, without symptoms (Figure 31). With these findings, the *Duke score* $\begin{array}{c} \left(\mathrm{DS}\right)=\mathrm{exercise}\;\mathrm{time}\;\mathrm{in}\;\min\mathrm{utes}-\left(5\times \mathrm{ST}\;\mathrm{deviation}\right)-\left(4\times \right.\\ \left.\mathrm{angina}\;\mathrm{index}\right) \end{array}$, or $\mathrm{DS}=+7.5-\left(5\times 3\right)-\left(4\times 0\right)=-7.5$, resulting in classification as intermediate risk. In the perfusion images, the presence of transient reduced uptake was evidently observed, involving the middle and distal portions of the anteroseptal and anterior walls and apex of the LV, with accentuated intensity and medium extent, compatible with significant ischemia in the territory of the anterior descending artery (Figure 32). Furthermore, mild transient dilation of the LV cavity was observed during stress, in addition to radiopharmaceutical uptake in the RV wall, which are high-risk markers.

**Comments:** Evidence in the literature has supported the use of CS for risk restratification in patients who have intermediate clinical risks (using, for instance the Framingham or the global risk score), but who are in asymptomatic phases. The higher the CS, the higher the risk will be; not coincidentally, the probability of silent ischemia will also be higher, and this, in turn, increases the patient risks even further. Data have demonstrated that, when CS values are between 400 and 999, the probability of perfusion defects reaches up to 29%, and when values are > 1,000, the probability increases to 39%.^241^ Brazilian data from Cerci et al. have reported similar information, with an ischemia prevalence of 34% in patients with CC over 400.^242^ Within medical orientation, rigorous preventative measures have shown evident benefits in individuals with high CS. Caution, however, is recommended when indicating revascularization procedures, emphasizing the absence of formal indication, consensus, or evidence regarding benefits based solely on CS results. On the other hand, recommendations exist for individuals with high-risk anatomy, at least moderate ischemic burden (in terms of extent and intensity), and the presence of symptoms refractory to clinical treatment.^14^ Notwithstanding indications documented in guidelines, levels of evidence demonstrating the benefits of myocardial revascularization with the aim of reducing mortality in patients with stable CAD, based both on information about anatomy^243,244^ and ischemia quantification (retrospective data),^245^ have been questioned, considering the absence of randomized studies published to date. With this in mind, what is known as the ISCHEMIA study^246^ was designed (report to the addendum of thi guideline), randomizing patients who have at least moderate ischemia (more than 10% of the myocardium affected by ischemia of significant intensity or severity) from 400 centers worldwide into 2 treatment scenarios: “optimized clinical treatment” *versus* “optimized clinical treatment associated with revascularization of ischemic territory,” excluding patients with left main trunk lesion > 50% on angio-CT. The study objective was to attempt to identify subgroups where revascularization benefits stable patients, filling in this important gap in current scientific evidence. Considering the available information (current evidence and guidelines), it seems appropriate to investigate ischemia in patients with CS over 400 in the attempt to identify individuals with high ischemic burdens (extent and intensity of perfusion defects), who may benefit from invasive strategies.

#### 6. Patient with abnormal ET, normal MPS, and normal coronary angio-CT results

**Clinical history:** male, age 33, atypical chest pain, recent onset of DM, high blood pressure (HBP) and family history of early CAD, ET resulting in intermediate-risk Duke score (+3).

**Figure 33:**
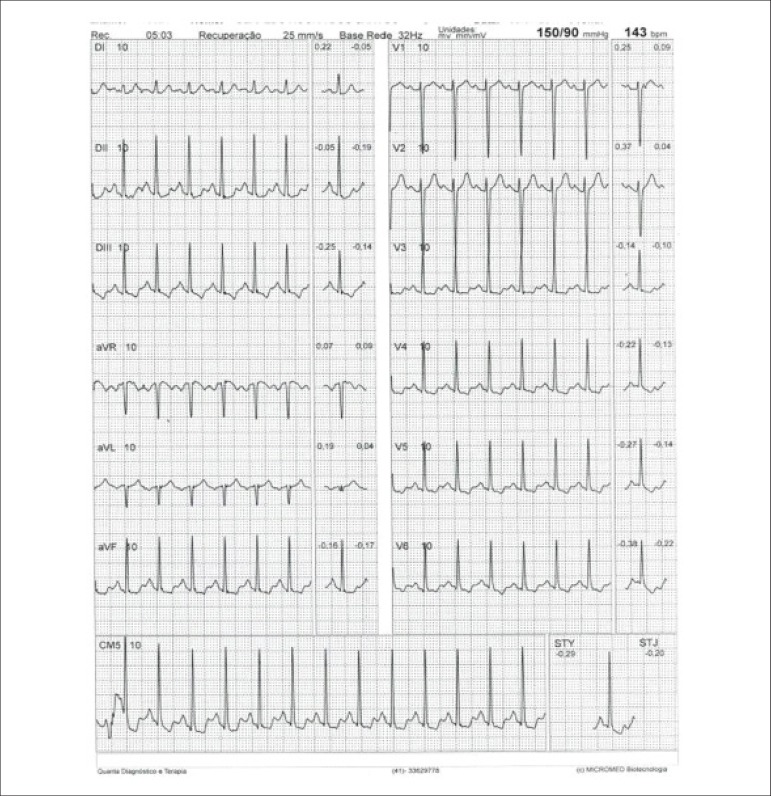
Case 6 - Electrocardiogram tracing showing electrocardiographic alterations (ST depression), especially during the recovery phase of the exercise c test.

**Figure 34:**
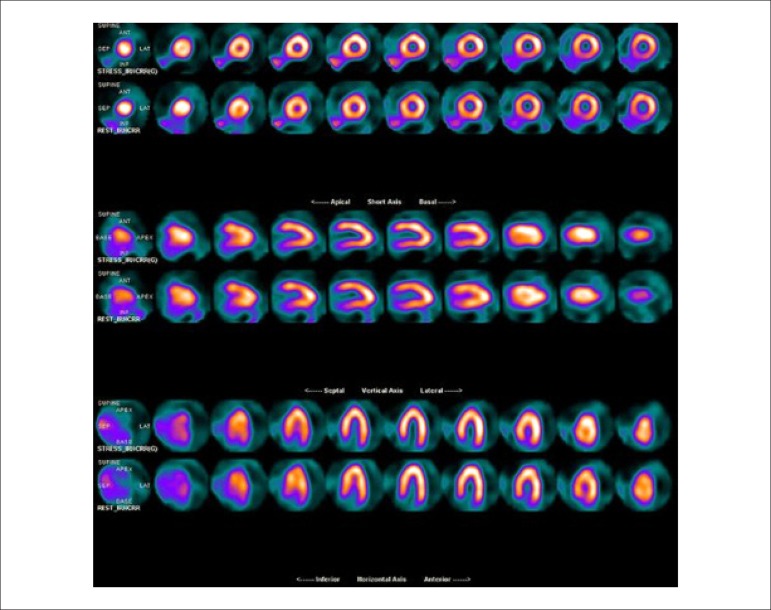
Case 6 - Myocardial perfusion scintigraphy within normality, with two image acquisition series, resting and stress, with dedicated cardiac equipment (gamma camera), equipped with conventional sodium iodide crystals.

**Figure 35:**
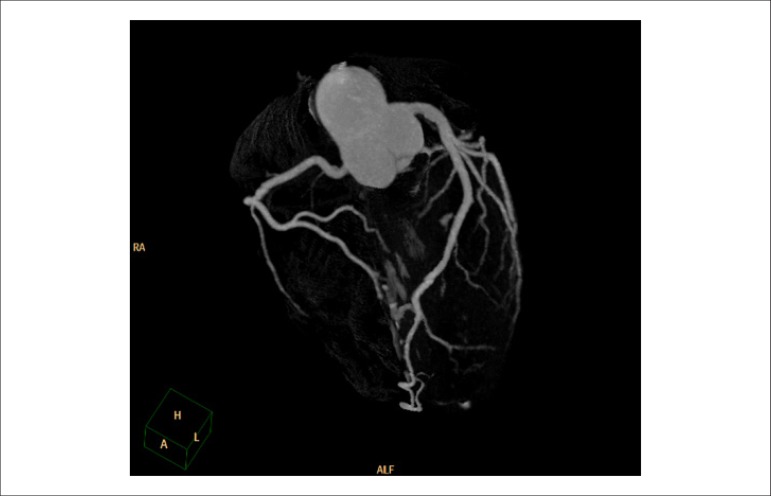
Case 6 - Coronary angio-CT within normality.

**Findings:** The stress phase of the ET revealed high estimated metabolic expenditure (11 METs), with 9 minutes in the Ellestad protocol. ST-segment depression of up to 1.5 mm (measured in the J point), with a descending aspect (Figure 33), were observed in multiple leads, during the recovery phase only, and they were sustained until the end of this phase. The Duke score was +3 (intermediate-risk), and MPS showed homogenous radiopharmaceutical distribution throughout the walls of the LV, considered within normal limits (Figure 34). Due to persistence of symptoms during evolution, coronary angio-CT was solicited 2 months later, showing an absence of obstructive lesions and a CS of zero (Figure 35).

**Comments:** In patients with intermediate probability of CAD, the indicated methods (MPS or angio-CT) are additional possibilities for investigation. At least 2 randomized studies have evaluated these strategies:

- The PROMISE study,^247^ which included 10,003 individuals with suspected CAD, with 25 months of follow up regarding the primary outcome (composed of death, AMI, and hospitalization for UA), showed similar evolution in both randomization groups (A) functional tests, including MPS, 5,007 patients with 3% events *versus* B) angio-CT, 4,996 patients with 3.3% events (p = 0.75). There was, however, a higher number of revascularizations in the group that began with anatomical evaluation.
- The International Atomic Energy Agency study^248^ demonstrated that the initial strategy of angio-CT entails the solicitation of additional diagnostic methods, including MPS itself or direct catheterization. These findings could have been foreseen due to the differences in information between both techniques, as coronary angio-CT is more sensitive for detection of anatomical diseases without important physiological impact. Like *Case 1*, the situation presented in *Case 6* is common in routine coronary angio-CT. In cases where ET shows intermediate or low risk and, especially, in those where the patient has intermediate or low pre-test probability, angio-CT has one of its most precise indications. The main diagnostic virtue of angio-CT is its high NPV which essentially excludes CAD. Thus, if the probability of disease is intermediate or low, the chance of excluding it is greater, and the test shows better benefits. In the case described, as symptoms persisted, complementary evaluation with angio-CT was highly useful to the medical decision-making process. Furthermore, prognosis in a patient without CAD on angio-CT is excellent, with nearly zero risk of AMI and coronary events for up to 5 years,^249^ owing to its high NPV and to the fact that characteristic evolution of CAD habitually progresses slowly, with individual variations, and this lowers the chances of an individual developing CAD culminating in a coronary event over a period of 5 years. Integrated analysis of these exams, in the case therefore, infers an excellent prognosis, notwithstanding the altered ET, and its rules out CAD quite safely, in the same manner that MPS had already excluded the presence of myocardial ischemia. On the other hand, if the present case had been associated with a high probability of CAD or inadequate technical conditions (e.g. high ventricular response atrial fibrillation), the scenario could have been different, given that, for a patient with high atherosclerotic burden, the positive predictive value of angio-CT is limited. Furthermore, artifacts caused by an unfavorable situation (AF) may severely impair diagnostic accuracy. In these cases, MPS would be a better form of evaluation.

#### 7. Patient with artificial electric pacemaker, abnormal MPS and normal angio-CT

**Clinical history:** female, age 49, diagnosed with Chagas heart disease, using an artificial pacemaker, pain during effort, type II DM, non-insulin-dependent, diagnosed 4 years prior.

**Figure 36:**
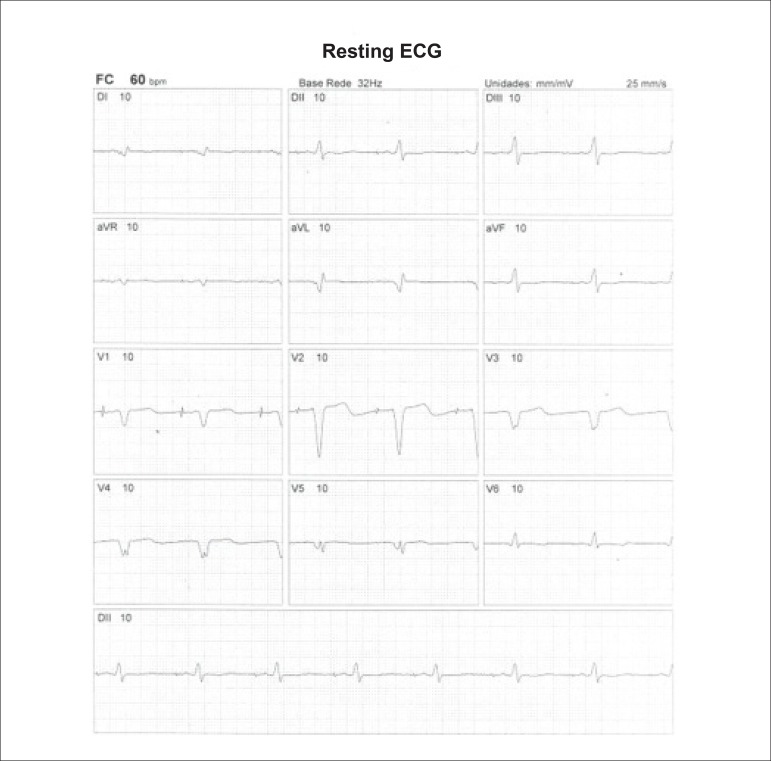
Case 7 - Electrocardiogram tracing of patient with artificial pacemaker.

**Figure 37:**
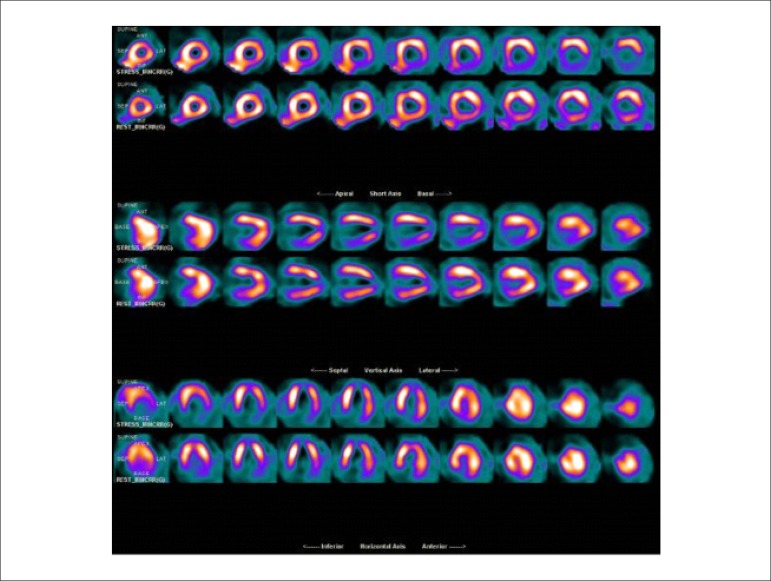
Case 7 - Myocardial perfusion scintigraphy showing significant perfusion abnormalities (notably following administration of dipyridamole, with partial improvement while resting) and dilation of the left ventricle. Image acquired with dedicated cardiac equipment (gamma camera), equipped with conventional sodium iodide crystals.

**Findings:** MPS performed with dipyridamole, considering the presence of artificial pacemaker stimulation (Figure 36) suggestive of DDD mode (resting ECG with atrial spikes, without clear visualization of ventricular command). Perfusion defects associated with pharmacological stress were characterized as moderate intensity and medium extent, involving the inferior (mediobasal portion) and inferolateral walls and the apex of the LV (Figure 37), and they were partially transient (predominance of ischemia). Angio-CT showed left dominant coronary circulation, with no signs of atherosclerosis. The presence of atrioventricular pacemaker electrodes limited assessment of the image via angio-CT.

**Comments:** Returning to the basic and appropriate principles of questions about pre-test probability and characterization of severity based on MPS findings, it is necessary to give special emphasis to the synergy between methods in this case. Symptomatic female patients with DM generally have an intermediate probability of obstructive CAD, mainly depending on the duration and aggressiveness of DM. On the other hand, they also have a high prevalence of endothelial dysfunction and microvascular disease, which may cause alterations in myocardial perfusion.^250^ There also exists the condition of Chagas heart disease, which features angina as a manifestation in the absence of obstructive epicardial coronary disease. The doubt which the doctor likely faces upon receiving the MPS results is the following: “What is the chance of obstructive CAD? And of endothelial dysfunction?” This is due not only to the diagnostic question, but also to the therapeutic implications, such as aggressiveness in reducing low density lipoprotein (LDL) cholesterol and the use of acetylsalicylic acid (ASA), for example. Another question is, “Does the perfusion modification in the apex represent an alteration related to Chagas heart disease?” Other questions similarly arise regarding the possibility of silent infarction related to CAD or a defect associated with artificial electrical stimulation resulting in customary atypical movement in the interventricular septum (component of an artifact). In this scenario, a safe and non-invasive way to exclude CAD is to perform angio-CT, which showed normal results in this case. It is important to underline the additional incremental prognostic value of angio-CT in this scenario, given that prognosis for this patient who does not have atherosclerosis, with mild ischemia (likely due to endothelial dysfunction), is considerably better than it would be were there conditions of mild ischemia in a patient suffering from uni- or bi-arterial obstructive CAD, or even in a patient with multivessel non-obstructive CAD.^251^ Other considerations refer to the possibility of MPS artifacts, not only related to the pacemaker in this case, but mainly to attenuation artifacts, when attenuation correction is not available, or when the prone position is not routinely used, in addition to the previously described Chagas heart disease. If an artifact is highly suspected, corroborated by the presence of systolic thickness of LV walls without alterations, angio-CT may avoid unnecessary catheterization, even in patients with higher probabilities of CAD. Within Brazilian experience, in a laboratory with a high nuclear cardiology volume, only 24% of patients with mild myocardial ischemia on MPS referred for angio-CT have obstructive CAD. The majority are women (58.8%), 33% of whom have non-obstructive CAD and 43% of whom do not have CAD (International Conference of Nuclear Cardiology - ICNC 2017).^252^

#### 8. Patient with abnormal angio-CT and normal MPS

**Clinical history**: male, age 51, atypical symptoms, active, with positive family history for early CAD.

**Figure 38:**
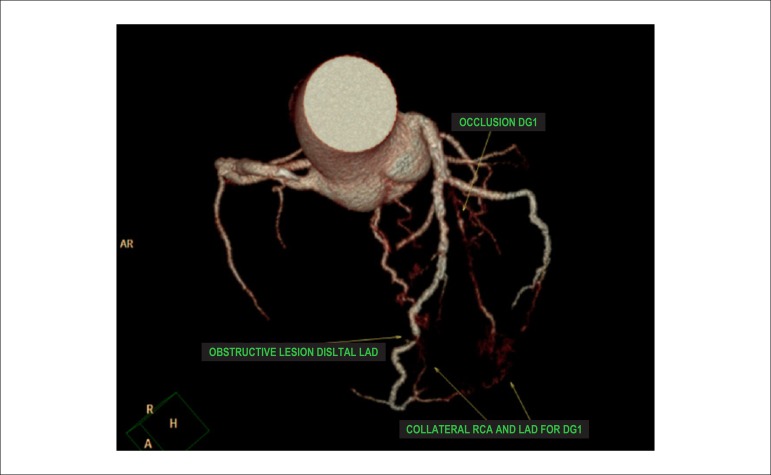
Case 8 - Angio-CT showing significant obstructive alterations and evidence of advanced coronary artery disease. DG1: diagonal 1 coronary artery branch; LAD: left anterior descending artery; RCA: right coronary artery.

**Figure 39:**
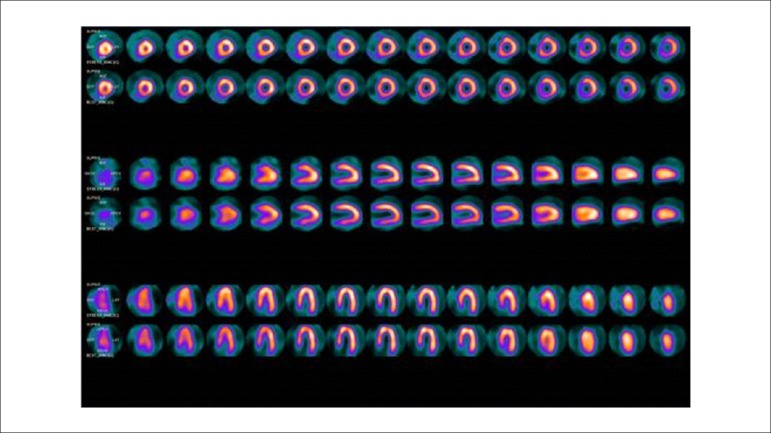
Case 8 - Myocardial perfusion scintigraphy within normality. Images acquired with dedicated cardiac equipment, equipped with solid cadmium-zinc-tellurium detectors.

**Findings:** Angio-CT showed a CS of 1,445 on the Agatston score (99% percentile); significant obstructive CAD involving the left anterior descending artery in its distal portion (> 70%), with occlusion of the first diagonal branch, which receives collateral circulation; and non-obstructive CAD in the circumflex and right coronary arteries (Figure 38). MPS with perfusion and LV function were considered within normal limits (Figure 39), and ET revealed optimal physical performance (estimated metabolic expenditure of 18 METs) and normal electrocardiographic, clinical, and hemodynamic responses.

**Comments:** This is a challenging clinical situation, which expresses a fundamental example of integration of non-invasive anatomical and physiological modalities with the goal of avoiding unnecessary revascularization procedures. Performing angio-CT as an initial exam had the objective of excluding obstructive CAD in a young patient with intermediate pre-test probability. Meta-analysis of recent studies has demonstrated a probable benefit of this initial anatomic strategy in this scenario, with reduced AMI, when compared to initial functional test,^253^ recently incorporated into guidelines in the United Kingdom.^254^ This investigation, however, leads to an increase in the number of invasive procedures and revascularizations, with the risk of these procedures not being appropriate.^245,246^ Thus, in the described scenario, with evident anatomy of obstructive CAD, which nevertheless does not meet the criteria for high risk (left main coronary lesion or triple-vessel lesions involving affected areas proximal to the left anterior descending artery), the management considered most appropriate is certainly ischemia quantification, considering that revascularization would be indicated in the presence of at least moderate ischemic burden.^241^ In this specific case, the patient should be clinically treated, with an aggressive secondary prevention approach, with close monitoring of modifiable risk factors and special attention to the manifestation of symptoms, postponing revascularization, at least in this moment where there is a lack of evidence regarding its benefits.

#### 9. Patient with abnormal angio-CT and abnormal MPS

**Clinical history:** male, age 51, with atypical chest pain (not always related to effort). Dyslipidemia and family history of early CAD (Both his father and brother had AMI resulting in death at the age of 53). Referred for coronary angio-CT to rule out obstructive CAD as the cause of the symptoms.

**Figure 40:**
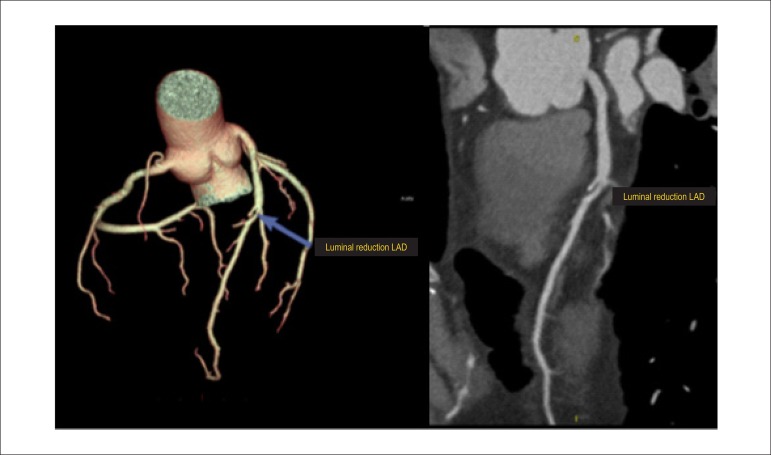
Case 9 - Angio-CT demonstrating significant obstructive luminal lesion, with the absence of calcification in coronary arteries. LAD left anterior descending artery.

**Figure 41:**
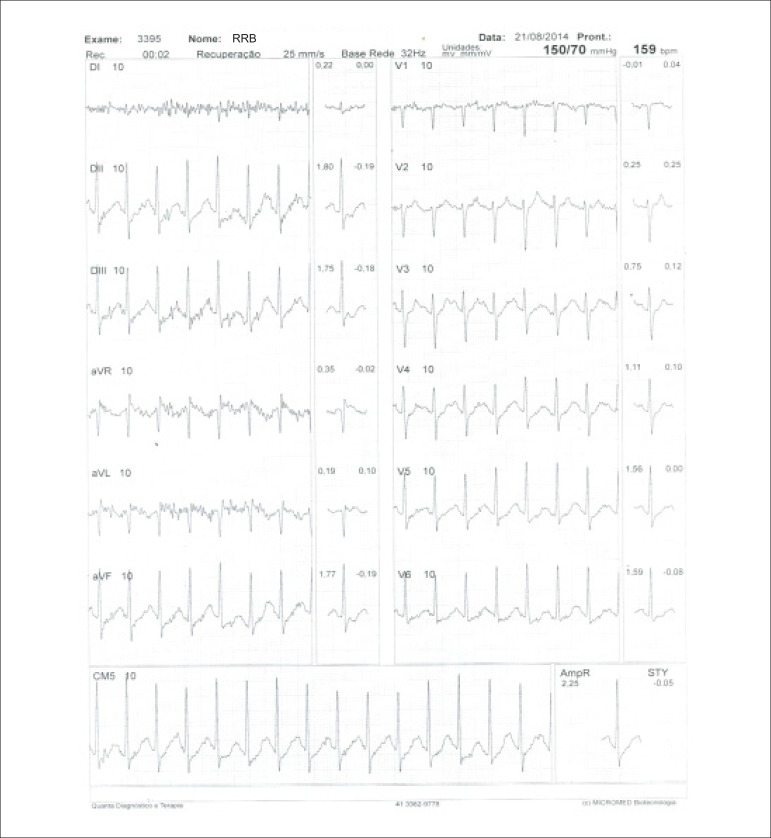
Case 9 - Electrocardiogram tracing obtained during immediate recovery period, with no significant alterations.

**Figure 42:**
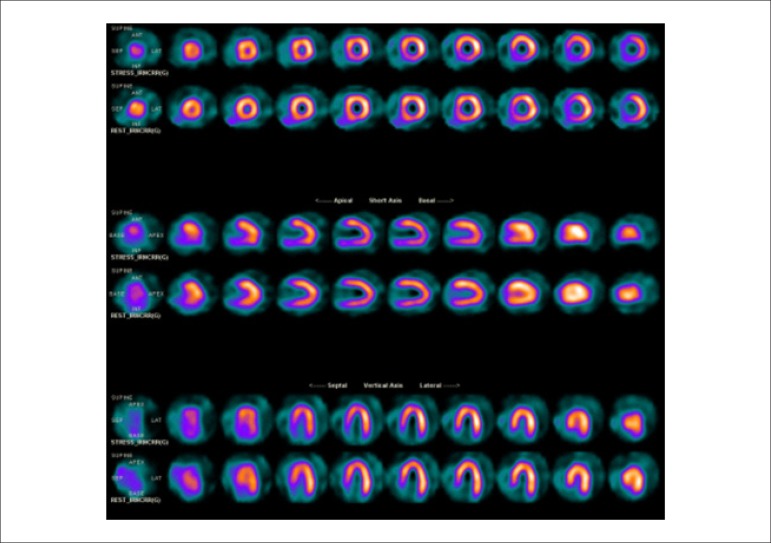
Case 9 - Myocardial perfusion scintigraphy demonstrating transient reduced uptake, characterized by mild intensity and small extent, suggestive of ischemia in the anteroseptal and septal walls and the apex. Images acquired with appropriate cardiac equipment (gamma camera), equipped with conventional sodium iodide crystals.

**Findings:** Angio-CT showed a CS of 12.2 (Agatston score, 67% percentile), mild, non-calcified atherosclerosis in the left main coronary and partially calcified atheromatous plaque in the middle third of the left anterior descending branch (Figure 40), resulting in moderate to significant luminal reduction (60% to 70%). The patient was referred for MPS associated with physical stress, exercising for 11 minutes in the Bruce protocol, with no significant ST-segment alterations (Figure 41) and without reproducing the symptoms. MPS images showed mild transient reduced uptake (ischemia) in the anteroseptal and septal walls and the apex of the LV (Figure 42).

**Comments:** Considering that a male patient with stable chest pain is characterized as having an intermediate pre-test probability of CAD, the routine non-invasive methods for diagnostic and prognostic evaluation are indicated. If the resting ECG is normal and the patient has informed ability to exercise (performing daily activities with estimated metabolic expenditure of > 5 METs), the ET is then the consensual indication, provided that its limitations are taken into account. In the case in question, the early family history of CAD stands out. This fundamental clinical information is not always incorporated into traditional methods of estimating pre-test probability. In this context, coronary angio-CT was chosen, in part to rule out obstructive CAD (high NPV), which is present in only 23% of symptomatic patients within the same probability range, according to the CONFIRM register.^240^ This register demonstrates lower observed prevalence of 50% to 70% obstructive lesions on angio-CT, in comparison with the expected prevalence calculated by conventional algorithms, establishing the concept that the routine algorithms for characterizing pre-test or expected probability of events during long follow-up periods, such as the Framingham, PROCAM, Diamond Forrester, SCORE, and Global Risk; overestimate CAD. This is also the case with detection of early, non-obstructive CAD (present in 34% of patients in this register), especially in patients with family history. This investigation strategy has already been shown to be effective and likely to reduce AMI,^252^ as previously discussed in this section; it is, however, necessary to be careful with excessive interventions. This was precisely the role of functional evaluation via MPS in this case. Detection and quantification of ischemia are fundamental for determining patient management, given that the presence of moderate to severe ischemia alone would justify a more invasive strategy, such as revascularization, in the absence of refractory angina. This case was thus started on optimal clinical treatment, similar to that of patients included in the COURAGE study.

#### 10. Patient with abnormal ET, normal MPS, and abnormal angio-CT

**Clinical history:** female, age 67, with fatigue related to effort. Hypertensive ex-smoker, diagnosed with diabetes 1 year prior. Referred for MPS following abnormal ET with intermediate risk.

**Figure 43:**
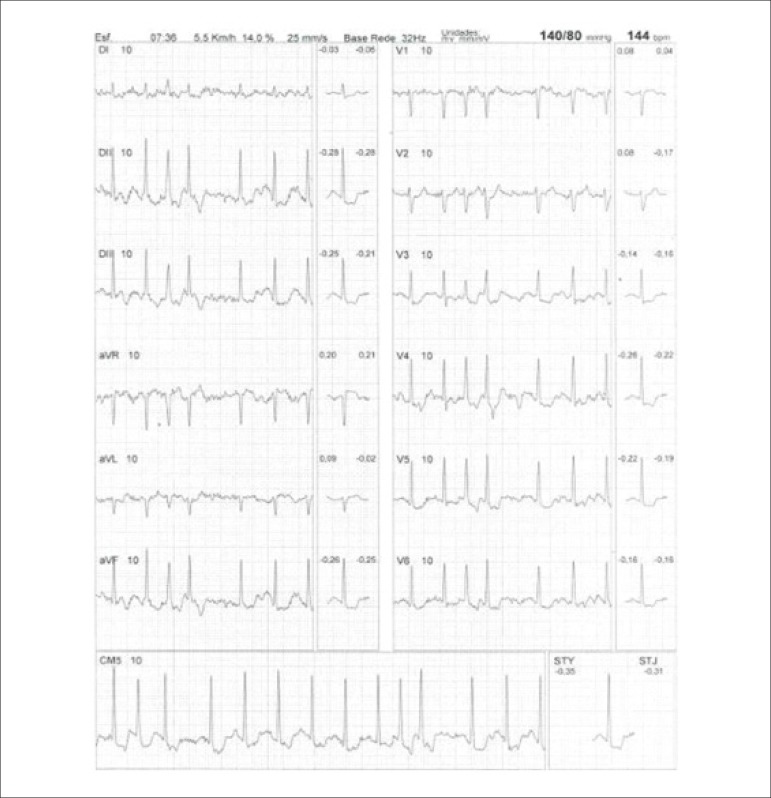
Case 10 - Electrocardiogram tracing demonstrates ischemic ST-segment alterations and episodes of supraventricular arrhythmia, in addition to nonsustained ventricular tachycardia.

**Figure 44:**
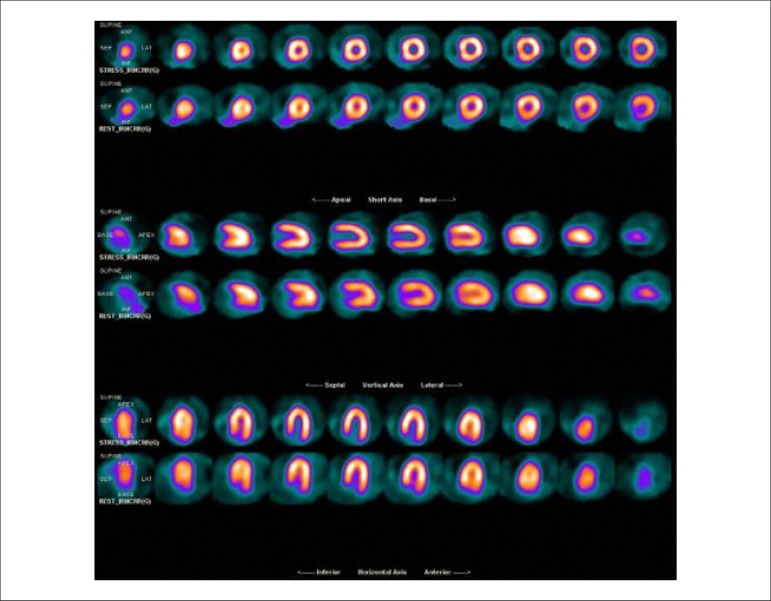
Case 10 - Myocardial perfusion scintigraphy with homogenous radiopharmaceutical distribution throughout the walls of the left ventricle, considered within the limits of normal. Images acquired with dedicated cardiac equipment (gamma camera), equipped with conventional sodium iodide crystals. Reproduced with the permission of Vitola JV.^234^

**Figure 45:**
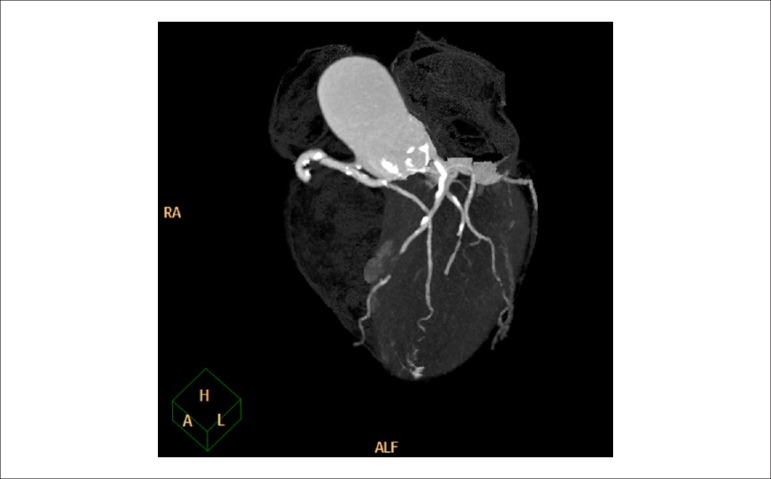
Case 10 - Coronary angio-CT showing significant coronary atherosclerosis, with multivascular calcification. Reproduced with the permission of Vitola JV.^234^

**Findings:** The patient exercised for 9 minutes in the Bruce protocol, reaching a HR of 136 bpm (89% maximum HR predicted based on age), triggering stress arrhythmias (ventricular and supraventricular extrasystole, in addition to periods of nonsustained ventricular tachycardia [NSVT]). ST-segment depression reached 3 mm in multiple leads (Figure 43), but the patient was asymptomatic. *Duke Score* = **-** **6**, characterized as intermediate risk. On MPS, there was an absence of signs of ischemia (Figure 44). Considering the clinical profile and the finding of complex ventricular arrhythmia (NSVT), concomitant with descending ST-segment depression, in spite of normal perfusion on MPS, the clinical option was to perform an angio-CT, which showed advanced atherosclerosis (Figure 45) with a CS of 829 on the Agatston score (97% distribution percentile) and non-obstructive lesions (< 30%) in multiple vessels.

**Comments:** In this patient, analysis of ET plays an important role in case management. MPS study with the radiopharmaceutical MIBI-^99m^Tc showed no abnormalities, which, in itself, determines excellent short-term prognosis. The presence of ventricular tachyarrhythmia during stress, however, adds a risk that is not, in practice, incorporated into risk prognosis by the Duke score. Furthermore, it limits analysis of the ST segment and may give rise to diagnostic doubts. One study which stands out in the literature verified that the inclusion of ventricular arrhythmia as a variable in the Duke score during ET increased its restratification potential in 30% of patients.^23^ Once again, questions may arise related to the lower sensitivity of MPS, which was apparently normal in this case (a false-negative result?). Another question is, “Should differential diagnoses such as cardiomyopathy, specific conduction tissue disease, among others, be additionally considered?” Although functionally severe obstructive CAD is improbable when MPS is normal, anatomical data from angio-CT complement and clarify many of these doubts which arose due to the conflicting results of the two functional tests. One alternative would be to perform CS, but, particularly in symptomatic patients, more detailed evaluated of anatomy via angio-CT, which quantifies degree of obstruction and determines the presence and extent of non-calcified atherosclerosis, adds incremental prognostic value.^256^ In this specific case, the presence of non-obstructive atherosclerosis, even with normal perfusion, denotes worse prognosis than in patients with normal perfusion and the absence of atherosclerosis.^257^ Moreover, the presence of atherosclerosis in multiple segments, as in the present case, confers a prognosis similar to that of uniarterial obstructive CAD.^250^ In conclusion, clinical translation of such findings could be resumed in the following manner: there are no indications that the ET alterations are secondary to ischemia, and there are thus no accrued benefits to coronary intervention, whether percutaneous or surgical revascularization. Orientation should be toward aggressive treatment of atherosclerosis to reach lipid goals recommended in current guidelines, in addition to strict control of other modifiable risk factors. The patient’s current profile confers medium- and long-term prognosis similar to that of a patient with obstructive CAD in a single coronary artery.

#### 11. Patient unable to exercise, abnormal MPS associated with pharmacological stimulus with dipyridamole and angio-CT showing non-obstructive CAD, ischemia suggestive of microcirculatory abnormalities

**Clinical history:** female, age 68, with stress fatigue. Hypertensive and obese, diagnosed with diabetes 8 years prior. Referred for MPS due to difficulty performing physical exercise test.

**Figure 46:**
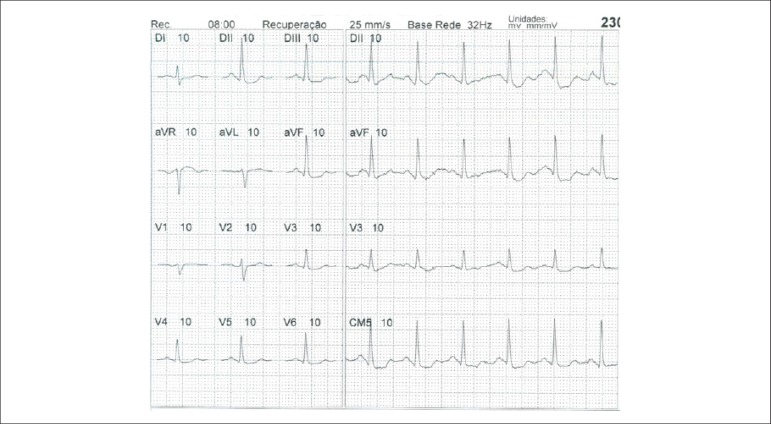
Case 11 - Electrocardiogram tracing with ischemic ST-segment alterations following administration of dipyridamole.

**Figure 47:**
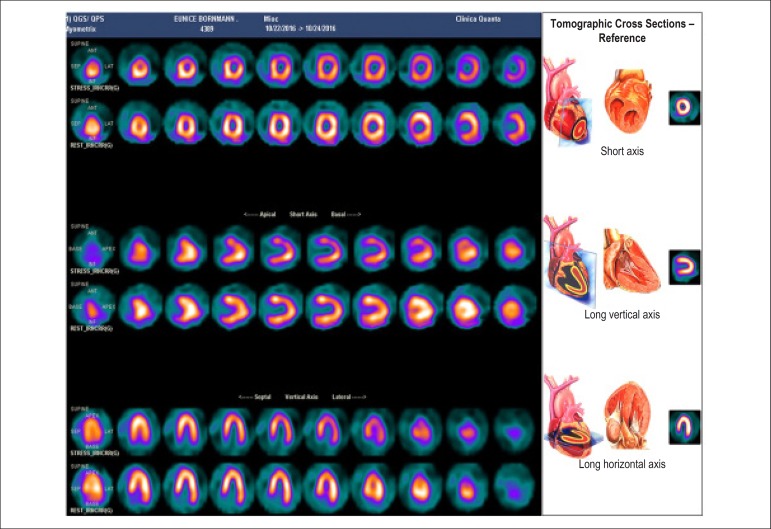
Case 11 - Myocardial perfusion scintigraphy (MPS) demonstrating moderate ischemic burden in the anterior wall, extending to the anterolateral wall of the left ventricle. Images acquired with dedicated cardiac equipment (gamma camera), equipped with conventional sodium iodide crystals.

**Findings:** During the attempted test with physical exercise, the patient exercised for only 6 minutes in the Bruce protocol, with a peak HR of 115 bpm (75.6% the expected upper limit, based on age). The stress phase was discontinued due to fatigue and calf-muscle pain, also establishing suspected chronotropic incompetence. As an alternative, the protocol was initiated with dipyridamole, and it was considered altered due to ST-segment depression of 1.0 mm, in 2 leads, following completion of intravenous administration (Figure 46). MPS was considered abnormal due to transient reduced uptake suggestive of ischemia, involving the anterior and anterolateral (predominantly in the middle distal portion) walls of the LV, with moderate intensity and medium extent, characterized as mild to moderate ischemic burden, in addition to preserved LV function (Figure 47). Considering the high clinical risk profile, the findings of probable chronotropic incompetence, ST-segment alterations with dipyridamole, and ischemia in the anterior descending territory; the option was to complement with angio-CT, which showed non-calcified and non-obstructive atherosclerosis, with a CS of zero and a mild lesion (< 30%) in the anterior descending branch.

**Comments:** This is a classic example of a symptomatic patient with a combination of factors which, in association, may result in phenomena of endothelial and microcirculatory dysfunction, with the consequent condition of myocardial ischemia. This physiopathological condition, little over a decade ago, would have led to coronary cineangiography study in order to rule out obstructive CAD. As a consequence, “normal” coronary arteries were often observed, in what were known as “white catheterizations.” With the findings described in specific populations, especially in female patients, the recent use of the term “ischemic heart disease” has gone on to express the conditions of obstructive atherosclerosis, endothelial dysfunction, and microvascular dysfunction more adequately. A recent review published by Pepine et al. in 2015 ^258^ described important differences in the CAD spectrum in both sexes, pointing out that symptomatic women have a lower prevalence of obstructive CAD than men with the same symptoms. On the other hand, they tend to have more microvascular dysfunction, plaque erosion, and thrombus formation. In this specific case, the following factors stand out: female sex, obesity, DM, altered functional tests (both the post-dipyridamole ECG and perfusion imaging via MPS), and non-obstructive CAD on angio-CT.

Based on this combination of individual characteristics, especially with endothelial dysfunction and reduced coronary flow reserve (CFR), associated with non-obstructive CAD, the risk of cardiovascular events is significantly higher, and it is similar to that of individuals who have obstructive CAD, but who are not indicated for revascularization.^259^ Thus, as the prevalence of obstructive CAD is lower in women, angio-CT has been growing as a preferential diagnostic method for ruling out obstructive CAD in patients with intermediate probability, especially when there are limits to the physical exercise test.^260^ In the case in question, the combination of functional (ischemia) and anatomical (non-obstructive CAD) data was essential to the diagnosis of endothelial dysfunction and to guiding therapeutic management.

#### 12. Abnormal ET characterized as intermediate-risk and abnormal MPS with high-risk indicators

**Clinical history:** male, age 69, with precordial pain during greater efforts for 4 months. Hypertensive, ex-smoker, referred for MPS due to altered ET, with intermediate Duke score.

**Figure 48:**
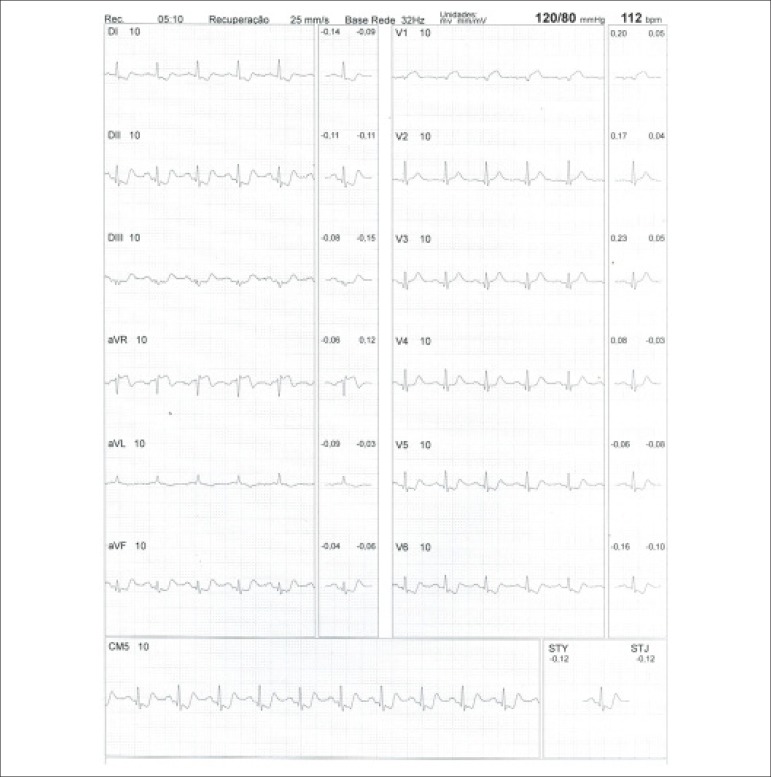
Case 12 - Electrocardiogram showing prolonged ischemic alterations during the late recovery phase.

**Figure 49:**
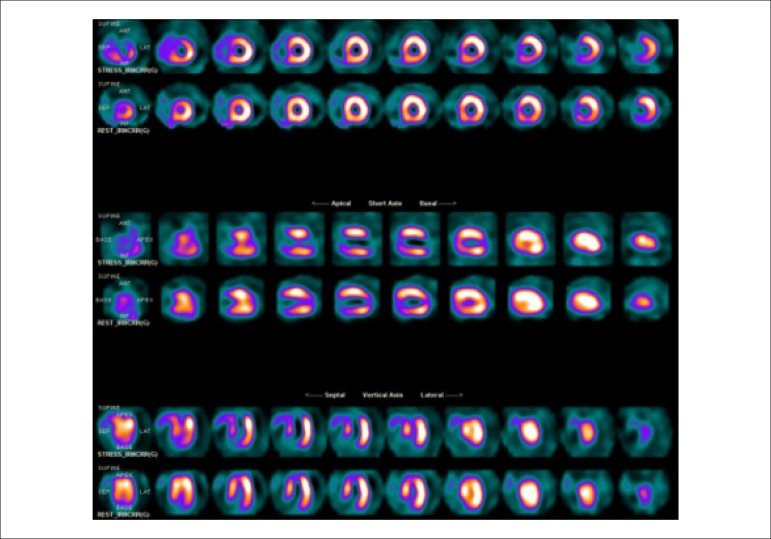
Case 12 - Myocardial perfusion scintigraphy (MPS) showing important ischemic findings (transient, stress-induced reduced uptake), with high-risk indicators, involving the territory of the left anterior descending artery (LAD). Images acquired with dedicated cardiac equipment (gamma camera), equipped with conventional sodium iodide crystals.

**Findings:** patient exercised for 9.5 minutes in the Bruce protocol, reporting non-limiting anginal pain during peak exercise, with descending ST-segment depression of 1.0 mm, measured at the J point, in multiple leads, with prolonged duration, during the recovery phase (Duke score = +0.5) (Figure 48). Perfusion imaging showed transient reduced uptake in the septum and apex of the LV, which was exercise-induced, characterized by severe intensity and medium extent (moderate ischemic burden), associated with the component of persistent reduced uptake in the described territory. Apical and septal akinesis (predominantly distal) were also observed following exercise, as well as apparent transient dilation of the LV cavity and uptake of MIBI-^99m^Tc in RV walls, which are additional markers of severity (Figure 49).

**Comments:** This is an example of a case where functional methods are in agreement regarding detection of ischemia. Cardiac imaging, here, provides additional information to the ET (intermediate-risk Duke score), which is known as “incremental prognostic value.” Quantification of ischemia via MPS restratifies this patient as high-risk. In this situation, revascularization procedures may be considered based on current evidence, although the results of the ISCHEMIA study (ISCHEMIA study Addendum - report to item 2 of this guideline) have not been yet published. This case is complementary to the discussion elaborated in Case 1, where non-invasive images via MPS added prognostic value and guided patient management.

## 10. Evaluation of Myocardial viability via Myocardial Perfusion Scintigraphy

### 10.1. Introduction

In some patients with chronic CAD and ventricular dysfunction, revascularization may significantly improve symptoms, ventricular function, and mortality. Physiopathological conditions and acute and chronic mechanisms of adaptation to temporary reduction of coronary flow include stunning, hibernation, and preconditioning, either separately or coexisting in the same patient.^261^ Evaluation of myocardial viability is, consequently, important to the therapeutic decision-making process for patients with ventricular dysfunction (class of recommendation I, level of evidence B) without angina,^262-266^ given that functional improvement will not occur in the presence of fibrosis. Several non-invasive imaging techniques are available for detection of viable myocardium, including stress echocardiography with dobutamine, cardiac resonance, and nuclear imaging with SPECT or PET. These methods evaluate different myocardial characteristics and, thus, present variations in sensitivity and specificity.^261^ In the SPECT technique, the use of thallium-201 (^201^Tl) is the established method for evaluating myocardial perfusion and the integrity of the cellular membrane. In comparison with other radiopharmaceuticals and available techniques, this has become the choice for determining viability.^267,268^

Additionally and in conjunction with these non-invasive techniques, whose aim is to obtain functional and/or anatomical information, constituting the basis of the physiopathological approach to underlying HF, multiple detector CT is also appropriate.

However, the selection of imaging modalities, whose purpose is to assess CHF and, specifically, viability of a dysfunctional myocardium, depends on the clinical information that is required for adequate patient management (Table 26), with inherent questions and affirmations, such as:

- Is the etiological cause of the cardiomyopathy in question ischemic or non-ischemic?
- In patients considered “ischemic,” the need for revascularization should be evaluated with respect to characterization of the quantity of myocardium at risk/viability.
- Evolving assessment of LV function and the possibility of remodeling are mandatory elements for analysis within the clinical decision-making process;^269^ other elements include secondary mitral regurgitation,^270^ implantable devices, such as defibrillators and/or resynchronization therapy.^271^
- Moreover, when the etiology of LV dysfunction, considered of the utmost magnitude for therapeutic decision making, is mandatorily under discussion, it has been verified that the majority of patients will have ischemic cardiomyopathy. Data from 24 multicenter studies on CHF, published in high-impact periodicals between 1986 and 2005 and summarized in 2006, including 43,568 individuals, showed a 62% prevalence of CAD. This frequency is probably underestimated to the extent that coronary cineangiography was not performed in all patients.^272^

**Table 26:** Clinical situations where nuclear cardiology should be considered a preference for assessing myocardial viability, in the following order of choice: PET with 18F-FDG, resting scintigraphy with thallium-201 and reinjection protocol, and resting scintigraphy with Sestamibi - ^99m^Tc sensitized with oral nitrite

• Evaluation of extent/localization of dysfunctional myocardium at risk, through significant hypoperfusion (hibernating myocardium)
• Clinical situations in which sensitivity is sought for assessment of viability
• Contraindications to the use of MR: patients with pacemakers or cardiac defibrillators incompatible with resonance, cerebral clips, cochlear implants, metallic fragments in their eyes, or renal insufficiency
• Conditions which limit image acquisition via MR: claustrophobia, irregular cardiac rhythm, dyspnea with inability to remain in dorsal decubitus for prolonged periods
• Availability of the method and local expertise

^18^F-FDG: fluorodeoxyglucose labeled with fluorine-18; MR: magnetic resonance; PET: positron emission tomography.

### 10.2. Morphology

It had initially been established that the recovery of ventricular function, when a hibernating myocardium was revascularized, should indicate that structural changes were absent or minimal, as observed in experimental models of stunned myocardium. However, since the beginning of the 1980’s, it has been known that chronically dysfunctional myocardial segments demonstrate distinct morphological changes under microscopy.^273^

There is a combination of normal, atrophic, and hypertrophic myocytes, with or without evidence of necrosis. Electron microscopy shows loss and/or disorganization of myofilaments and alterations in sarcoplasmic reticulum and mitochondria. These structural changes may contribute to slow functional recovery following revascularization.^274^

### 10.3. Evaluation of Viable Myocardium

The differentiation between the presence and absence of viability is highly relevant in patients under consideration for revascularization. Many patients who demonstrate viability associated with severe LV dysfunction may still be candidates for revascularization, but not for cardiac transplant.^275^

### 10.4. Physiopathology and Definitions

- The term viable myocardium, regardless of the contractile state of the myocardium, should be understood differently in the context of CHF and viability study, given that the main objective is to predict improvement in LV function following revascularization.
- Persistent contractile dysfunction of the LV may be related to chronic hibernation and/or stunning, and not merely associated with fibrotic tissue. Revascularization may improve function and survival if the dysfunctional myocardium is still viable. Functional improvement will not occur in the presence of fibrosis.
- The initial point for discussion of viability implies regional dysfunction, detected by various non-invasive methods, including echocardiography as an initial line of investigation.
- Differentiation between hibernation and stunning may be established based on blood flow to the myocardium. While resting flow is chronically diminished in hibernation, it may still be preserved in the stunned myocardium, with a compromised flow reserve however.^19^
- Clinically speaking, it may not always be feasible to separate the 2 physiopathological conditions; nor is it always necessary, considering that both entities require revascularization in order for there to be improvements in ventricular function. Affected areas are referred to as viable myocardium or myocardium at risk.^276^
- The coexistence of normal myocardium, however, and the formation of subendocardial scarring will not result in improved function, which is now considered “non-jeopardized” area of viable myocardium.
- Most experience in assessing viability has been obtained with nuclear imaging, utilizing SPECT and PET, with assessment of perfusion and metabolism. Moreover, using SPECT, cellular and mitochondrial membrane integrity may be characterized.
- It has been demonstrated that 40% to 50% of dysfunctional segments without contractile reserve may still have preserved perfusion and metabolism, some of which will recover function following revascularization procedures. The loss of contractile reserve is associated with structural damage characterized by greater severity and fibrosis formation.
- Several viability standards have been recognized in areas of contractile dysfunction, for instance: I) any region with > 50% of radiopharmaceutical uptake in resting images; II) any perfusion defect with > 10% increase in uptake of late images.^277^ It is, however, necessary to emphasize that areas with > 50% uptake often do not improve in function, given that these regions contain mixtures of normal myocardium and non-transmural scarring.
- Uptake and retention of tracers (sestamibi and tetrofosmin, labeled with technetium-99m) depends on perfusion, cellular membrane integrity, and mitochondrial function, where radiopharmaceutical uptake of > 50% to 60% in dysfunctional areas is frequently used as a sign of viability, when observed in resting images.

### 10.5. The Most Frequently Used Protocols^24,278,279^

1. **Stress and redistribution - two steps or image acquisition series/one injection of ^201^Tl:** This is a conventional technique with image acquisition between *2 and 10 minutes* (a maximum of 15 minutes) following injection of ^201^Tl during peak stress (*first step*). These images reflect initial *distribution* of the radioisotope dependent on blood flow and, thus, regional myocardial flow. Two to four hours after initial intravenous administration of the radioisotope, in the resting condition (*second step*), a new series of images is obtained, representing the “*redistribution phase*,” related to the continuous exchange of ^201^Tl throughout the myocardium and extracellular behavior. This protocol has been designed to study ischemia, and it is not sufficient for characterization of viability, given that viable tissues may not exhibit improvement in radiopharmaceutical uptake (reversibility) within conventional time periods for redistribution images, giving the apparent impression of persistent reduced radioisotope uptake or fibrosis.
2. **Stress/redistribution and reinjection - three steps or image acquisition series/two injections of ^201^Tl (Figure 50B):**In addition to the conventional protocol, which contains only *1 injection of ^201^Tl* during *stress* (at a dose of up to 3.0-3.5 mCi), this includes the *reinjection of ^201^Tl* (generally at a dose of 1 mCi) *immediately after the redistribution phase*, with the aim of elevating blood concentration of the radioisotope, with a new image acquisition series that may vary in terms of time (between 6 to 24 hours). There is evidence that up to 50% of regions with perfusion defects that are apparently “fixed or persistent” improve in terms of relative radioisotope uptake. This information is predictive of improvement in regional function following revascularization. Areas with sustained or severe reduced uptake following reinjection show a low probability of recovering ventricular function. Further variations are demonstrated in **Figures 50 C** and **D**.
  **Figure 50:**
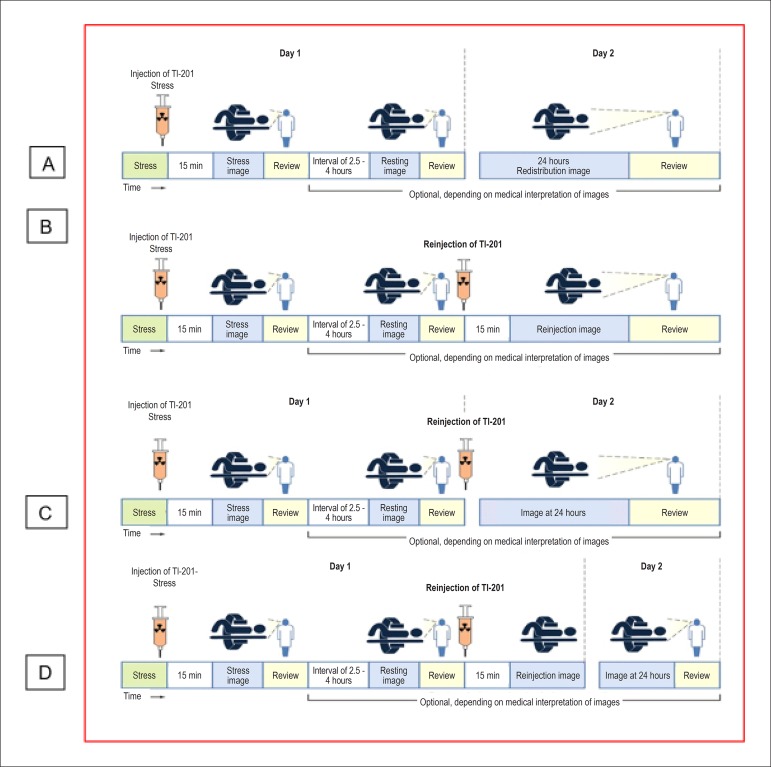
Imaging protocols with the use of thallium-201 (^201^Tl) for characterizing viability of the myocardium. A represents the classic sequence for defining ischemia (Day 1), with the injection of a dose of approximately 3 to 4 mCi during stress, without subsequent reinjection, adding new image acquisition 24 hours later (Day 2); B, C, and D emphasize the need for reinjection of ^201^Tl with an approximate dose of 1 mCi and sequencing as demonstrated in the proposed protocol.
3. **Stress/redistribution and late imaging - three steps or image acquisition series/one injection of ^201^Tl (Figure 50A):** The addition of *late imaging 24 hours* after injection of thallium-201 during the *stress or distribution phase (first step)* allows more time for the phenomenon of redistribution to occur and, consequently, for increase in myocardial uptake of the radiopharmaceutical. This technique shows good predictive value for improvement of ventricular function following revascularization, but suboptimal NPV owing to the technical quality of images obtained after this period, as well as to the fact that some patients do not demonstrate redistribution during very prolonged periods.
4. **Resting/redistribution - two steps or image acquisition series/one injection of ^201^Tl while resting:** This protocol eliminates the stress phase, based on knowledge of the physiopathology of temporal variations in coronary flow in hibernating myocardium or in unstable patients, leading to perfusion defects that may occur in resting studies, with redistribution in late images. Qualitative studies using the *resting-redistribution* protocol to evaluate efficacy of revascularization have shown that the majority of myocardial regions with reversible defects during pre-operative periods presented post-operative normalization in perfusion and/or improved ventricular function. Some viable regions, however, may not present redistribution, even in images at 24 hours, unless ^201^Tl blood levels are elevated following reinjection. Practically speaking, most of the clinical information necessary to the medical decision-making process, related to viability study, will have been obtained during the *stress-redistribution-reinjection* sequence, and late images will generally not be necessary. There is no established consensus regarding the accuracy of Sestamibi - ^99m^Tc while resting for detection of viability, with some studies showing similar uptake for ^201^Tl and Sestamibi - ^99m^Tc (quantitative assessment) in patients with UA and LV dysfunction. It has also been observed to have satisfactory predictive value for improvement in contractility in the ventricular walls following revascularization, similar to that of ^201^Tl.^280^ The administration of nitrates before injection of the radiopharmaceutical with the objective of improving resting myocardial flow seems to improve accuracy for detecting myocardial viability.^281^

### 10.6. Positron Emission Tomography^282,283^

This non-invasive exam is the reference for detecting myocardial viability, as it simultaneously offers information on myocardial perfusion and metabolism. In normal fasting and aerobic metabolism conditions, while resting, long-chain free fatty acids (FFA) represent around 65% to 70% of energy supply to the cardiac muscle, with a lower participation of glucose (15% to 20%),^21^ whereas, in post-prandial conditions, glucose becomes the preferred substrate. In the presence of ischemia, the oxidative metabolism of FFA is diminished and glucose also becomes the preferred myocardial substrate. In this manner, even if the energy produced by anaerobic glycolysis is not enough to maintain myocardial contractility, it is vital for the preservation of cellular membrane integrity, which is characteristic of dysfunctional, yet viable myocardium. Some positron emitters, such as fluorine-18, labeling a glucose analogue or fluorodeoxyglucose (FDG), may be used to evaluate viable (or hibernating) myocardium, which is defined as metabolically active tissue, in the presence of a coinciding perfusion defect. FDG-^18^F penetrates the cells of the myocardium by the same transport mechanism as glucose and, following phosphorylation to FDG-6-P, remains in the intracellular medium in proportion to the rate of glycolysis, reflecting the quantity of viable tissue. Patients are typically prepared for imaging with administration of glucose overload and subsequent doses of insulin, prior to intravenous injection of 5 to 15 mCi of FDG-^18^F. Images are acquired 45 to 90 minutes after the administration of the tracer, lasting, approximately, 15 to 30 minutes. Low-dose CT is required for attenuation correction. The combination of perfusion and metabolism imaging via PET has a sensitivity of 88% and a specificity of 74% for myocardial viability.^284^ Some differences exist for patients with diabetes, for whom the preferred form of viability evaluation is with ^201^Tl or agents bound to ^99m^Tc. It is also important to remember that the radioisotope fluorine-18 or ^18^F is produced in a cyclotron, consisting of the bombardment of enriched water labeled with oxygen-18 or ^18^O and decaying via positron emission, with an energy range of 511 keV. Its half-life is 110 minutes, allowing for the best spatial resolution among radiotracers used for PET.

When using PET for joint analysis of metabolism with FDG-^18^F and perfusion (positron emitters are not available for this in clinical practice in Brazil), an excellent predictive value for functional recovery has been observed. Measurements of MBF are typically performed with rubidium-82 (^82^Rb) or ammonia labeled with nitrogen-13 (^13^NH_3_). Based on the concept of hibernating myocardium, segments with reduced perfusion, but with preserved FDG uptake (known as mismatches), are classified as viable, with functional improvements following adequate revascularization. However, when perfusion and FDG uptake are diminished or apparently absent (matches), this reflects an absence of viability (areas of fibrosis), which do not show improvements after revascularization. Finally, the combined approach to blood flow-FDG mismatches has been widely document as a predictor of post-revascularization regional improvements in motility, as well as improvements in symptoms of HF, exercise capacity, and prognosis. The PET and Recovery after Revascularization (PARR-2) study,^285^ which is still the only randomized prospective study to evaluate the benefits of results of a management strategy assisted by PET in patients with severe LV dysfunction, evaluated 430 randomized individuals for viability study with PET or conventional treatment without the use of PET. Results of primary analysis after 12 months of follow-up have showed a tendency toward improved results in the FDG-assisted PET, which was not statistically significant. On the other hand, post-hoc analysis, including only patients who adhered to the management strategy recommended based on the findings of FDG PET, showed significant improvements in mortality with the PET-assisted approach, when compared to standard care. Di Carli et al.^286^ demonstrated that small areas of viable myocardium (more than 5%), identified by PET, stratified patients into subgroups of high-risk of cardiac events in a year. Equally, comparative studies on viability between PET and SPECT with ^201^Tl demonstrated agreement of results in 80% of cases.

### 10.7. Additional Information Based on Evidence within the Medical Decision-making process for Patients with Congestive Heart Failure, Decreased Left Ventricular Ejection Fraction, and Viable Myocardium

**Figure 51:**
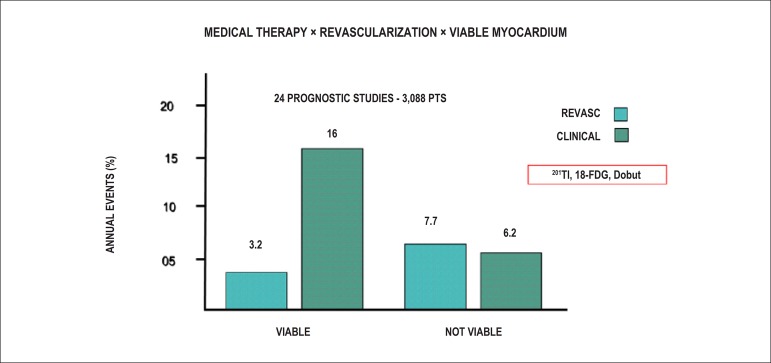
Prognostic studies in patients with severe chronic coronary artery disease and ventricular dysfunction evaluating late survival with revascularization versus medical therapy following viability study via non-invasive testing. ^201^Tl: thallium-201; FDG-^18^F: metabolic imaging with fluorodeoxyglucose labeled with fluorine-18; Dobut: doppler echocardiography associated with intravenous injection of dobutamine solution; Revasc: myocardial revascularization surgery; Pts: patients; Viable: viable myocardium. Adapted from Allman KC, et al.^287^ Note that in the presence of viable myocardium and clinical treatment, the rate of events was > 4 times (16%) higher than in the same situation with surgical treatment via myocardial revascularization (3.2%); however, in the situation of absence of viable myocardium, the comparison between clinical and surgical treatment showed no significant differences in mortality (6.2% vs. 7.7%, respectively).

A classic meta-analysis involving 24 studies and 3,088 patients (Left Ventricular Ejection Fraction - LVEF 32% ± 8%), with the use of different imaging methods, demonstrated that patients with viability had significant reductions in mortality when comparing surgical revascularization treatment and clinical treatment, with no benefits for either group in the absence of viability (Figure 51).^287^

In contrast, a recent multicenter prospective randomized study, known as the Surgical Treatment for Ischemic Heart Failure (STICH) study,^288^ was unable to show benefits regarding mortality between patients randomized into revascularization surgery (CABG), in comparison with optimized medical therapy (OMT), in primary intention-to-treat analysis, for patients with dilated cardiomyopathy (LVEF ≤ 35%) of ischemic etiology (the first of 2 tested hypotheses, involving 99 centers in 22 countries).

SPECT and/or Doppler echocardiography, associated with a low dose of dobutamine, were used to characterize viability, based on established methodological criteria. Of the 1,212 patients included for evaluation, 601 underwent the aforementioned viability studies, comparing 298 receiving medical therapy and surgical revascularization (CABG Group) to 303 patients with OMT. Of the total of 478 patients with viable myocardium, the outcome of death occurred in 178 (37%), in contrast with 58 (51%) of deaths in 114 patients without viability (hazard ratio: 0.64; 95% CI: 0.48-0.86; p = 0.003). However, after adjusting for other baseline variables, the association with mortality was not significant (p = 0.21). There was significant crossover between the 2 groups and the analysis in question was in favor of better results with CABG.

In spite of these negative findings, recognizing biases in critical analysis of the results in this occasion, it is understood that, in the real world, faced with current evidence,^289-291^ viability study may be of assistance when choosing the best treatment in selected populations, leading to better prognosis in evolution.

## 11. New Technologies and Future Perspectives for Nuclear Cardiology in Studying Ischemic Heart Disease

Established experience and extensive documentation, which has been accumulated over the past decades, have demonstrated that MPS has satisfactory sensitivity and specificity, emphasizing good NPV for ruling out obstructive CAD. Analysis of 32 studies has shown that SPECT has a sensitivity of 87% and a specificity of 73% for detecting significant angiographic lesions (stenosis > 50%).^292^ Some limitations to the conventional technique have been observed, such as restricted spatial resolution, reduced counting rates, and attenuation of artifacts. Anger gamma cameras with sodium iodide crystals and photomultipliers, which transform emitted gamma photons into light or scintillation, also have limited temporal resolution in comparison with other imaging methods, such as PET, and they require higher doses of radiotracers, besides to carry out exams with longer image acquisition times. On the other hand, the innumerous advantages of the nuclear method include: **a)** the utilization of radioisotopes that do not alter the biological properties of the organism being studied; **b)** high radioactive labeling with a minimal amount of chemical substances, faithfully representing physiology and cellular biochemistry; **c)** minimal toxicity; **d)** pixel (smallest component of a digital image) values of myocardial images are directly proportional to parameters inherent in cardiovascular physiology, such as perfusion, function, metabolism, and innervation, attributes which are not shared by other modalities, such as angio-CT, cardiac resonance, and echocardiography; **e)** another aspect which stands out is the superior contrast resolution for detecting perfusion abnormalities, differentiating normal and hypoperfusion myocardium with great accuracy, facilitating visual and quantitative image analysis.^29,293^

**Evolution of hardware:** As exposure to radiation and its long-term deleterious effects have become important concerns on the part of regulatory authorities and scientific societies, new technologies have been introduced to reduce doses of radiotracers in nuclear exams while maintaining image quality and diagnostic accuracy. In this context, new equipment with CZT detectors arose in the first decade of 2000. Differently from traditional Anger gamma cameras, gamma radiation is directly converted to electric pulses upon contact with the CZT detectors, increasing energy resolution and dispensing with photomultipliers, which makes the detectors much finer and lighter. They are also distinct from older conventional cameras in terms of better spatial and energy resolution and the ability to distinguish dispersed radiation, in addition to being more sensitive for detection of emitted photons.^294-296^

Duvall et al.^297^ have shown the viability of a reduced-dose protocol and of reduced doses in a study carried out with a dedicated CZT gamma camera (Discovery NM 530c, GE Healthcare), using 5 mCi of ^99m^Tc as a resting dose and 15 mCi of ^99m^Tc as stress dose to label sestamibi or tetrofosmin. There was a significant reduction in exposure to radiation in relation to anterior SPECT protocols, and image quality and accuracy for diagnosing CAD were maintained. Gimelli et al.^298^ have also evaluated the viability of a stress-resting protocol with a reduced dose, used a CZT camera in a cohort of 137 patients referred for evaluation of CAD, with subsequent coronary cineangiography. Accuracy for identifying coronary lesions was not affected by reduced radioisotope dosage, and high sensitivity and specificity values were obtained. Hindorf et al.^299^ showed that the ideal patient position should be established when performing myocardial exams with CZT gamma cameras.

In addition to evaluating diagnostic accuracy, it has become important to evaluate prognostic value of new imaging protocols using CZT cameras. Oldan et al.^300^ compared prognostic value between CZT and conventional gamma cameras, observing that prognostic information provided by CZT technology, regarding the outcome composed of all-case mortality and non-fatal AMI, was similar to that provided by traditional equipment (Anger camera). The same dose, however, was administered to both gamma-camera groups. This fact limited the study’s ability to evaluate prognostic value with reduced-dose protocols, as previously described.

Brazilian researchers^301^ compared the prognostic values of an ultrafast low-dose protocol with a CZT camera and a traditional protocol with a conventional gamma camera with sodium iodide crystals. By means of a propensity score, 2 groups with equal numbers of patients and similar baseline characteristics were compared. The average dose of radiation in the first group (which underwent the exam with a conventional camera) was estimated at 9.5 mSv, whereas the CZT group had an average exposure dose of around 6 mSv. This dose was lower than the effective average SPECT doses mentioned in previous studies. It was confirmed that the CZT camera protocol showed similar prognostic results when compared to those obtained with traditional SPECT cameras, emphasizing that patients with MPS on CZT had a lower events rate than those who underwent MPS with traditional gamma cameras.

**Evolution of software:** Filtered back-projection (FBP) is a traditional reconstruction algorithm in LV imaging, which has was used in many SPECT gamma cameras over time. It considers that the radiation emitting object, in this case the heart, is at the same distance from all detectors and that the photons are also uniformly detected at all angles. This supposition, however, leads to a large number of image artifacts, which may be caused by breast attenuation and loss of counting density at higher distance from the heart, for example. FBP, thus, presents limitations that lead to the development of new iterative reconstruction images, which allow for the correction of these inherent artifacts.^302^

New algorithms have been developed, the most widely used of which is known as ordered subset expectation maximization (OSEM). This iterative reconstruction technique is based on estimated projection of the object studied, with additional comparison between the acquired and estimated projections of the object. The projection of proportion is generated, containing the differences between real and estimated projections of the object. These differences are used to modify initial estimation, and every cycle in this chain is called an iteration. Iterations are carried out until a projection more similar to the real object has been achieved. Iterative reconstruction allows for correction of image artifacts, such as dispersion, attenuation, and noise suppression during the reconstruction process, in order to improve image quality and resolution.^303^

DePuey et al.^304^ described the use of OSEM for processing exams with different acquisition periods (7 to 15 minutes) performed with a dual-head gamma camera with high resolution collimators. It was demonstrated that, notwithstanding lower time, image quality was maintained or even improved by the use of these reconstruction methods. Additionally, in a Brazilian study, Lima et al.^305^ analyzed prognostic accuracy of a new reconstruction algorithm, “Evolution for Cardiac TM,” with reduced dose and acquisition time in a dedicated gamma camera (Ventri, GE Healthcare), with an average dosimetry of 6.2 ± 0.3 mSv. The 2,958 patients who underwent exams were followed for approximately 3 years, and their results demonstrated a very low rate of major events (death or infarction), when imaging was normal in comparison with the group with abnormal imaging exams.

**New perspectives:** MPS may have some imaging limitations in patients with multivessel CAD, due to loss of comparative perfusion parameters between different areas of the myocardium, considering that image generation is based on relative uptake of coronary flow labeled with radiopharmaceuticals between the walls of the LV, as described in the introduction to these Guidelines. Given this situation, quantification of absolute coronary flow and coronary flow reserve (CFR) has arisen as an important alternative for diagnostic and prognostic evaluation of these patients. Falcão et al.^306^ demonstrated that evaluation of flow reserve via PET assists in the detection of CAD in patients who have left bundle branch blocks. Patients with apparently normal perfusion and abnormal CFR have higher annual rates of cardiac death, non-fatal myocardial infarction, late revascularization, and hospitalization due to cardiac causes than patients with normal perfusion and normal CFR (6.3% *versus* 1.4%; p < 0.05).^307^ Ziadi et al.^308^ have expanded these results in a prospective study involving 704 patients who underwent injection with rubidium-82 (^82^Rb) for PET, with the objective of comparing results in patients with reduced or normal CFR and patients with normal or abnormal perfusion exams. Reduced CFR was an independent predictor of major events, including cardiac death and myocardial infarction, adding prognostic value to the perfusion results. Murthy et al.^309^ studied the predictive power of CFR in 2,783 consecutive patients, observing a 5.6-fold increase in the risk of cardiac death in patients with lower CFR values, compared to patients with higher values. This variable showed incremental prognostic value in comparison to relative analysis of perfusion and LVEF. The prognostic value of myocardial flow reserve (MFR) as a variable goes beyond CAD. This has been demonstrated in patients with ischemic and non-ischemic cardiomyopathy,^310^ hypertrophic cardiomyopathy,^311^ and post-cardiac transplant.^312^

The measure of coronary flow reserve using PET is quite accurate, but as it is expensive, it is not widely available in clinical practice, especially in Brazil. However, as SPECT technology is an easily accessible tool, studies show the possibility of acquiring dynamic images and quantifying MFR with this method, with some limitations, however, when using traditional gamma cameras, including limited temporal resolution.^313,314^ With the advent of CZT cameras, it has become viable to quantify CFR using this technology.

Wells et al.^315^ developed a pig model for measuring absolute myocardial blood flow and flow reserve, using three different radioisotopes, namely, ^201^Tl, Tetrofosmin-^99m^Tc and Sestamibi-^99m^Tc. Following in this research area, Bouallègue et al.^316^ obtained CFR in 23 patients with known three-vessel disease using a CZT gamma camera, successfully obtaining good correlation with angiographic findings. New discoveries related to dynamic SPECT image acquisition with measurements of CFR are opening new and exciting research fields that will allow for different applications of SPECT to the extent that their results are better validated.

## 12. Strategies for Reducing Exposure to Radiation

Ionizing radiation refers to radiation with enough energy to “ionize” atoms and molecules during its interaction with matter, in this case, with elements in the human body. Depending on the type and level of energy used, on the duration of exposure, and on the dose absorbed, damage to the organism may occur. Professionals involved in medical exams or therapies that make use of ionizing radiation should be familiar with the basics of what is known as the “**a**s **l**ow **a**s **r**easonably **a**chievable” (ALARA) principle. This stipulates that an individual’s exposure to radiation must be minimized, regardless of reason for exposure. In relation to nuclear imaging, this means obtaining the principal exam information, most commonly MPS, using the minimum amount of radiation necessary to maintain diagnostic quality. This involves not only the choice of radiotracer, but also the best technique and the best protocol that may be adjusted to minimize radiation exposure.

Furthermore, 2 other important principles guide the application of ionizing radiation for medical imaging: “**justification”** and “**optimization.”** *Justification* signifies that a study should be well indicated and justified as adequate, in following with appropriate criteria, as described in these guidelines. The best way to minimize a patient’s exposure to radiation is not to recommend an exam that is not appropriately indicated. This situation, however, is often beyond the control of the doctor responsible for performing the exam and should be understood by the referring doctor, who should have a basic grasp on the scientific literature. It is important to emphasize that the risk of a patient dying due to cardiovascular disease (the leading cause of death in Brazil), without undergoing a well indicated exam, is much higher than any eventual risk owing to radiation exposure. The risks of a patient with suspected coronary disease who has an appropriate indication for an exam are not theoretical, but rather real, and they are higher than the risks resulting from radiation use. The other principle which demands our attention is *optimization*, which represents adjustments to protocols, including the use of the best technological resources available (modern software and hardware) to perform an exam. This task is the responsibility of a multiprofessional team, made up of physicians, supervising nurses, nursing technicians, radiology technologists, biomedical specialists, biologists trained to manipulate radioactive material, professionals with training in radiopharmacy, and a team of medical physicists. The risks of deleterious effects of radiation involving patients, provided that the multiprofessional team uses the best technique and consistently observes the principles of justification and optimization, are minimal, and they are, at times, the fruit of theoretical elaboration with no consolidated practical base.

The INCAPS Nuclear Cardiology Protocols Cross-Sectional Study (INCAPS), an important, recently conducted international study coordinated by the International Atomic Energy Agency (IAEA), involving 65 countries, including Brazil, verified that radiation exposure may be different when comparing patients living in different countries, given the diversity of implemented protocols in nuclear cardiology practice worldwide.^317^ Innumerous opportunities have been found to improve the application of nuclear cardiology comprehensively worldwide, basically using the principle of *optimization*. It has generally been identified that with simple, low-cost orientations, such as adjusting dose to BMI, it is possible to reduce the administered dose of radiation and patient exposure significantly. Latin America is an example,^318^ where there are opportunities to improve protocols. It has been recommended that the majority of patients undergoing MPS have a maximum estimated radiation exposure of 9 mSv. This goal is feasible to reach when applying the recommendations listed in Table 27, which are strategies supported by the IAEA.^24^ In the Brazilian centers that participated in the INCAPS study, average doses were observed to vary between 8.4 and 17.8 mSv, thus demonstrating the possibility of optimizing protocols in the country, which has been undertaken since the publication of INCAPS.^318^

**Table 27:** Strategies for reducing radiation exposure

1	Divulgate and apply appropriate exam indication criteria
2	Give preference to tracers that result in lower exposure to radiation. It is currently recommended to use technetium-99m (^99m^Tc), which has a physical half-life of 6 hours, in comparison with thallium-201 (^201^Tl), whose half-life of 73 hours results in unfavorable dosimetry
3	Avoid protocols that inject both radioisotopes, ^201^Tl and ^99m^Tc, in the same study (dual-isotope protocol), also considered an unfavorable dosimetry in combination
4	Avoid injecting any dose of ^99m^Tc which is > 36 mCi or which results in an exposure dose of > 15 mSv for the complete exam
5	In the event that it is necessary to use ^201^Tl, avoid doses over 3.5 mCi
6	Attempt to decrease the number of patients who perform both stress and resting phases. In some cases, it is possible to answer the clinical question with the stress injection, provided that images are perfectly normal during this phase
7	Utilize more sensitive equipment which is able to detect lower injected doses of radiopharmaceutical, such as new gamma cameras with solid cadmium-zinc-telluride (CZT) detectors, as well as software which improves imaging quality, even at lower administered doses
8	Apply the dose adjustment table based on patient's size or body mass index (BMI) (Tables 28 and 29)
9	Avoid shine through. This phenomenon occurs in perfusion studies with ^99m^Tc (one-day protocol, resting and stress phases) when the dose of the second injection is less than 3 times the first dose, and the residual activity in this step may interfere with interpretation of images corresponding to the second injection. This situation may result in a non-diagnostic study, making new investigations necessary and thus increasing the patient's total received dosage.

**Table 28:** Suggestions for dose limitation, adjusted to body mass index (BMI), using conventional gamma camera technology^24^

**One-day protocol**		
**BMI**	**Dose 1 (mCi)**	**Dose 2 (mCi)**
< 25	8	24
25 to 30	9	27
30 to 35	10	30*
> 35	12	36*
**Two-day protocol**		
**BMI**	**Dose 1 (mCi)**	**Dose 2 (mCi)**
< 25	8	8
25 to 30	9	9
30 to 35	10	10
> 35	12	12

* Give preference to the two-day protocol.

**Table 29:** Suggestions for dose limitation, adjusted to body mass index (BMI), using gamma cameras with cadmium-zinc-telluride technology^24^

**One-day protocol**		
**BMI**	**Dose 1 (mCi)**	**Dose 2 (mCi)**
< 25	4	12
25 to 30	4.5	13.5
30 to 35	5	15*
> 35	6	18*
**Two-day protocol**		
**BMI**	**Dose 1 (mCi)**	**Dose 2 (mCi)**
< 25	4	4
25 to 30	4.5	4.5
30 to 35	5	5
> 35	6	6

* Give preference to the two-day protocol.

### 12.1. Reducing Radiation Using New Technologies, Image Quality, and Reliability of Findings

Given that exposure to high doses is a source of concern, considering the possibility of biological effects of ionizing radiation, which are known as *deterministic* (those which occur above certain limits of absorbed dose in a determined tissue, including skin erythema, loss of hair, and, possibly, direct cardiac toxicity) and *stochastic* (those whose radiation causes damage that may lead to malignancy which is generally long-term),^319,320^ there has been a demand for new technology to reduce doses of radiotracers, maintaining image quality and diagnostic accuracy. In this context, new cameras with CZT detectors have been launched in recent years. Differently from traditional Anger cameras, gamma radiation is directly converted into an electric pulse when it comes into contact with CZT detectors, increasing energy resolution and dispensing with photomultipliers, which makes the detectors much finer, lighter, and more sensible to photon detection. Protocols have suggested that it is possible to combine faster exams with lower dosimetry. Owing to high costs, they are still not widely used in Brazil, where there are few more than a dozen gamma cameras with this new technology , which has already begun to contribute relevant publications to the international scenario.^321^

One of the limitations, known as the “Achilles heel” of myocardial perfusion image interpretation is the attenuation which gamma rays undergo when they pass through tissues before reaching the detector. The problem lies in the fact that attenuation may simulate myocardial perfusion defects, and their recognition as cases of “false-positive” results greatly depends on the observer’s experience. These defects occur more frequently in the inferior wall in men (especially in patients with abdominal obesity), and they are described as diaphragmatic attenuation. In women, they are most commonly found in the anterior wall, due to attenuation caused by breast tissue. These imaging defects are more common in obese patients, for which reason they require higher injected doses. When the defects found are tenuous, when they occur in a similar way during resting and stress, and when they are accompanied by thickness in the walls of LV without abnormalities, the myocardial perfusion study may frequently be interpreted as normal, thus sparing the patient other investigations, both those that involve radiation and those that do not, and minimizing costs. Another way to lower the attenuation artifacts is to apply specific machines with other sources of radiation, such as an x-ray emitting tomograph, which calculates tissue attenuation factors and applies attenuation correction to the photons emitted by the radioactive tracer, reducing the effects of attenuation by means of software. This equipment, however, adds costs to the exam, and it is difficult in terms of financial viability. Even in the USA, which is the country with the highest volume of myocardial scintigraphy studies, it is estimated that only 20% of medical services routinely apply this method. Finally, another method, which is widely applied in practice to resolve apparent defects in uptake generated by attenuation of gamma photons when they penetrate tissue, is to acquire images in the prone and supine positions , especially during the stress phase, which significantly reduces the artifacts described. Some services have routinely implemented this practice, even considering the increased gamma camera utilization time with a new image acquisition series.

With respect to performing stress exam alone in order to reduce radiation by avoiding the resting dose, the following should be considered:

1. It is possible for the observing doctor to succeed in interpreting a study as “absence of ischemia,” based only on stress imaging, thus avoiding the resting injection. In order to do this, the observer must be confident that the image is perfectly normal and free of any perfusion defects, including “attenuation artifacts,” which are generally recognized by comparing stress and resting images. The alternative is described above, routinely applying attenuation correction, but this would involve increased costs.
2. Another situation, which is not frequent, but which may occur, is when the patient has homogenous tracer distribution in the LV and apparently normal perfusion, but with an observed transient increase or dilation in the same cavity during the stress phase, when compared to resting images (transient ischemic dilation). The following may, additionally, be observed: **a)** drop in LVEF following stress, compared to resting; and/or **b)** increased uptake in the RV (generally not visualized) during the phase corresponding to stress, in comparison with resting; these are markers of poor prognosis, even in the presence of perfusion which is apparently “normal” owing to homogenous distribution. In these situations, the patient is exceptionally identified as high-risk based on other findings which are not solely related to tracer distribution in the LV during stress. This analysis and these findings are only possible when comparing resting and stress images. In summary, the doctor interpreting the images should be sure that the stress exam is perfectly normal, in order to dispense with resting image acquisition. Whenever possible, nuclear cardiology should always be performed using exercise as a preferential form of stress instead of pharmacological tests, which serve as alternatives only in patients who are unable to exercise efficiently. In the presence of severe multivessel coronary disease (involving three arteries) and apparent relative homogeneous radiopharmaceutical distribution during stress, it is extremely rare not to observe other high-risk findings during stress testing, in addition to deterioration of LV function induced by stress itself (ischemic myocardial stunning).

In summary, in this section, various ways to reduce radiation exposure in patients undergoing nuclear cardiology exams have been covered. By means of relatively simple adjustments to protocols, injected doses may be optimized, guaranteeing image quality and, most of all, reliability of findings in an exam which is of great clinical importance to orient patient management.

## 13. Evaluation of Cardiac Sympathetic Activity by Scintigraphy with ^123^I-MIBG

### 13.1. Introduction

Autonomic cardiac innervation plays a fundamental role on cardiac performance by regulating myocardial blood flow, HR, and myocardial contractility. In several cardiac diseases, cardiac neuronal function is altered and frequently associated with worse evolution. Dysregulation of the autonomic cardiac nervous system increases the risk of potentially lethal arrhythmias and may be a marker of poor prognosis. Scintigraphy imaging of distribution and cardiac neuronal function facilitates the comprehension of the physiopathology of various diseases that affect the heart and may guide treatment, with consequent improvements to clinical results.^322^

The autonomic cardiac nervous system encompasses sympathetic and parasympathetic innervation, with its norepinephrine (NE) and acetylcholine neurotransmitters, respectively. These work in equilibrium, and they exert *stimulating effects*, via adrenergic receptors, and *inhibitory effects*, via muscarinic receptors, both of which are responsible for electrophysiological and hemodynamic adaptations in the cardiovascular system, in response to bodily demands.^322^

In this way, sympathetic stimuli are controlled by cerebral regulatory centers that integrate signals coming from other parts of the brain and receptors in the body. Efferent signals follow descending pathways in the spinal cord and make synapses with preganglionic fibers that emerge at levels T1 to L3. In this sequence, they establish synapses with the paravertebral stellate ganglion and innervate the RV, in addition to the anterior and lateral regions of the LV. In the heart, sympathetic nerves follow the coronary arteries in the subepicardium and then penetrate the myocardium.^322,323^

Parasympathetic fibers are scarce in number in comparison with sympathetic fibers, and they originate in the marrow, following the vagus nerve. They begin in the epicardium, crossing the atrioventricular groove and penetrating the myocardium. They are located in the subendocardium, predominantly innervating the atria, but they are less dense in the ventricles, with the exception of the inferior wall. They greatly control the function of sinoatrial and atrioventricular nodes.^322^

### 13.2. Cardiac Scintigraphy with ^123^I-MIBG

Metaiodobenzylguanidine (MIBG) is a molecule with a structure similar to that of NE, obtained by means of modifications to the molecular structure of guanethidine (a false neurotransmitter, also an NE analogue), which acts selectively on sympathetic nerves, without, however, being metabolized by monoamine oxidase or catechol-O-methyltransferase or exercising a stimulating effect, as NE does. For the objective of diagnosis, MIBG is labeled with iodine-123, forming the radiotracer MIBG-^123^I,^322-325^ demonstrating a good correlation between myocardial uptake of MIBG-^123^I and NE content in cardiac tissue.^325^ After the radiopharmaceutical is injected via intravenous administration, it spreads throughout synaptic space, and is taken up, concentrated, and stored in presynaptic nerve endings in a manner similar to that of NE. MIBG-^123^I is retained and localized in cardiac sympathetic nerve endings with scintigraphy images obtained by a conventional gamma camera.^322-325^

The radioisotope iodine-123 predominantly emits gamma photons with an energy of 159 keV and a physical half-life of 13.2 hours, making image acquisition easy and well tolerated. MIBG-^123^I is widely used in Europe and Japan, and it has recently been approved for cardiac use in the USA.^326^ In Brazil, this technique is available in some centers. Cardiac scintigraphy with MIBG-^123^I directly evaluates global and regional sympathetic function of the heart, including uptake, reuptake, storage, and NE release processes in presynaptic nerve endings.^327^

**Figure 52:**
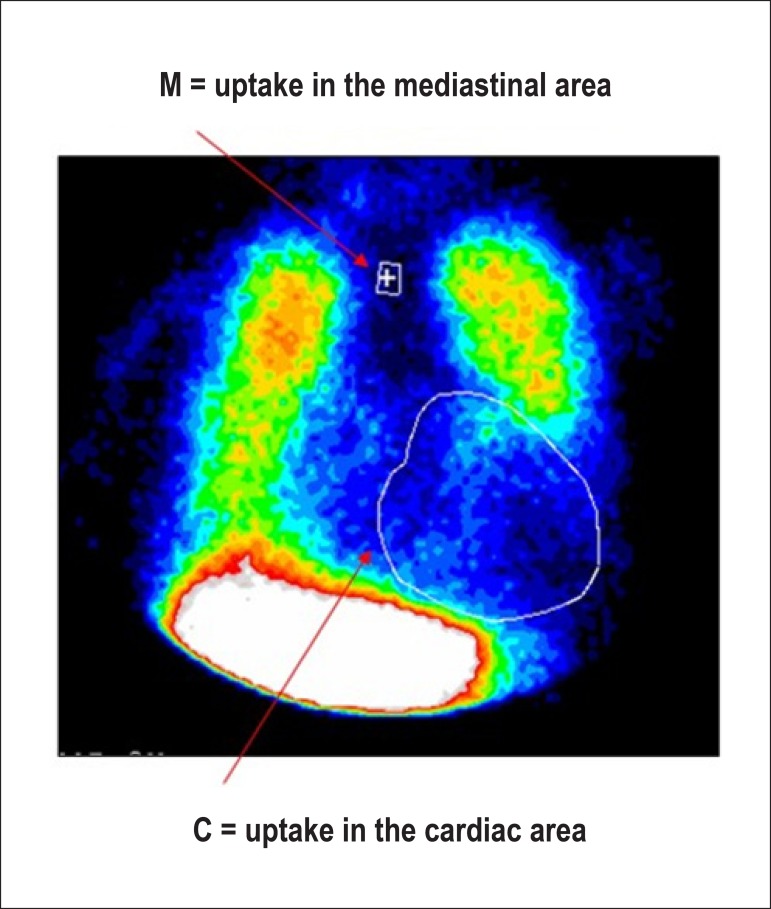
Cardiac scintigraphy with MIBG-^123^I. Colored late imaging of the anterior thorax with the regions of interest (ROI) drawn in the mediastinal (M) and cardiac (C) areas, to calculate the heart to mediastinum ratio (HMR) of MIBG-^123^I uptake. This index is the measure of the ratio between the statistical count of the rate of average radiation per pixel in the ROI, drawn in the heart and mediastinum.

Intravenous injection of MIBG-^123^I is administered while resting, at least 30 minutes after oral administration of potassium iodide syrup or an iodine-containing solution, in order to block and protect the thyroid. Medications that may potentially interfere with catecholamine uptake, such as antidepressants, and some calcium channel blockers should be suspended for at least 24 hours before administration of the radiopharmaceutical. On the other hand, betablockers, angiotensin converting enzyme inhibitors (ACEI), and/or angiotensin receptor blockers (ARB) do not need to be discontinued.^328^ Approximately 15 minutes and 4 hours after administration of MIBG-^123^I, static images and tomography images (SPECT) of the thorax are obtained in anterior projection, with the patient in dorsal decubitus, with the left arm raised above the thorax. While tomography images are optional, they help evaluate myocardial sympathetic activity and compare with the perfusion study (*see the subsequent topic “Evaluation of arrhythmias”*). Global cardiac uptake of MIBG-^123^I is evaluated by static imaging of the thorax (Figure 52). The following 2 fundamental parameters are visually and semiquantitatively analyzed: *the relation between cardiac and mediastinal uptake* (heart to mediastinum ratio [HMR]) in *early images* (15 minutes after injection) and *late imaging* (4 hours after injection) and the *myocardial washout rate* (WR) of MIBG-^123^I.^322-324,327-330^

**Figure 53:**
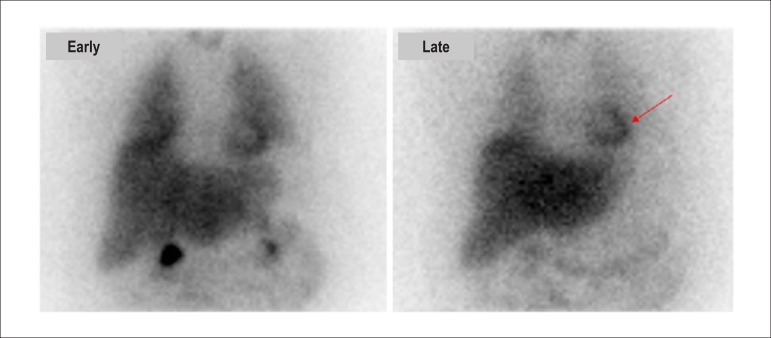
Cardiac scintigraphy with MIBG-^123^I showing normal radiopharmaceutical uptake in the cardiac area (arrow), which denotes preserved cardiac sympathetic activity. Black and white images of the anterior thorax, acquired approximately 15 minutes (early) and 4 hours (late) following intravenous injection of the radiopharmaceutical MIBG-^123^I.

**Figure 54:**
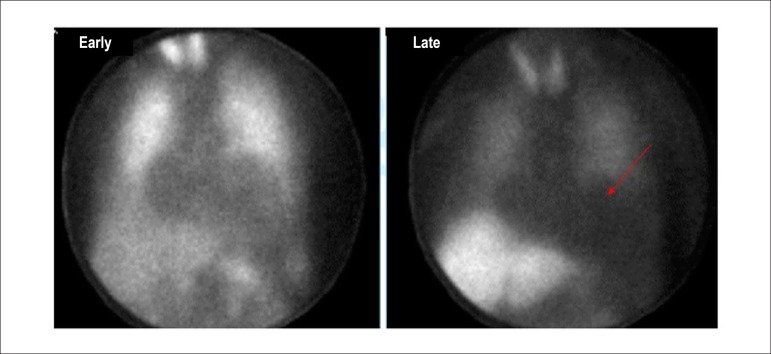
Abnormal cardiac scintigraphy with MIBG-^123^I of a patient with advanced heart failure and highly reduced radiopharmaceutical uptake in the cardiac area (arrow), which denotes cardiac sympathetic hyperactivity. Black and white images of the anterior thorax, acquired approximately 15 minutes (early) and 4 hours (late) following intravenous injection of the radiopharmaceutical MIBG-^123^I.

Normal HMR values vary from 1.9 to 2.8 with an approximate average of 2.2 ± 0.3 in late images, with results ≥ 1.6 considered predictive of lower risks.^331^ HMR reflects the density of receptors and, probably, depicts the integrity of presynaptic nerve endings and of the uptake 1 receptor. This elevated index indicates a predominant localization of the radiotracer in the myocardium, which is expected in normal hearts, to the extent that the finding of reduced HMR indicates lower myocardial uptake of MIBG-^123^I, translating as reduced density of cardiac adrenergic receptors. Late HMR combines information on neuronal function of uptake and the release of storage vesicles in cardiac nerve endings. Figures 53 and 54 show images of patients with normal and altered HMR, respectively.

Myocardial washout rate (WR) of MIBG-^123^I is also an important measure of cardiac sympathetic innervation. WR is calculated as the difference in myocardial uptake between the early and late phases and is determined by the percentage of reduction in uptake between these steps, reflecting the amount of catecholamines released in the cardiac synaptic cleft. Cardiac sympathetic hyperactivity is associated with reduced retention of MIBG-^123^I in late images (reduced late HMR) and increased WR. Normal WR control values are 10% ± 9%.^332^ Higher values are predictive of worse prognosis,^333^ although there are several methods for determining this ratio in the literature. Intra- and inter-observer variability of these measurements is less than 5%.^328^

### 13.3. Clinical Applications of Cardiac Scintigraphy with MIBG-^123^I

Imaging studies of cardiac adrenergic activity may be useful in several clinical scenarios (Table 30), including: HF, ventricular arrhythmias (associated with HR and primary arrhythmias), ischemic heart disease, DM, patients undergoing cardiotoxic chemotherapy, pre- and post-cardiac transplant, Takotsubo syndrome, and cardiac involvement due to systemic conditions, as in Parkinson’s disease. Generally speaking, this exam has been established to have an effective capacity for risk stratification of patients with the previously described conditions, but the utility of this information in improving clinical results of patients has not yet been demonstrated.^331^

**Table 30:** Established and potential indications for cardiac scintigraphy with MIBG-^123^I

Clinical Scenarios	Established	Potential
HFrEF	• Risk stratification, regardless of other parameters; evaluation of progression of HF, arrhythmic events, and total cardiac mortality up to 2 years • Identification of a low-risk subgroup for cardiac events and mortality • Clinical follow-up of medical therapies indicated in the guidelines	• Identification of patients most likely to benefit from CRT or LVAD • Guiding treatment of patients with LVAD: bridge to transplant, possible explant • Substitute marker for evaluating benefits of new medical therapies and devices
HFpEF	• Subanalyses of larger studies have shown a risk stratification similar to that seen in patients with HFrEF	• Identification of patients whose risks may be higher than clinically apparent
Arrhythmias associated with HF	• Risk stratification for lethal or potentially lethal ventricular arrhythmias for up to 2 years • Identification of patients with very low risks of lethal arrhythmic events for up to 2 years	• Refining indication criteria for patients who will benefit from ICD • Helping identify patients who will no longer need ICD, at the end of battery life or device infection
Primary arrhythmic conditions	• Identification of patients with risks of worse outcomes, including arrhythmic events and total mortality	• Improving understanding of physiopathology of primary arrhythmic conditions • Guiding conduct for patients with primary arrhythmic conditions
Heart transplant	• Following post-transplant cardiac reinnervation after	• Identification of patients who are more likely to have complications following transplant, including transplant rejection and transplant by CAD
Ischemic heart disease	• Evaluation of area at risk in patients with acute coronary syndromes • Risk stratification in patients with hibernating myocardium	• Guiding conduct for patients with acute coronary syndromes • Guiding conduct for patients following ischemic events • Ischemic memory
Diabetes mellitus	• Identification of cardiac autonomic abnormalities, including patients without extracardiac manifestations	• Identification of patients whose risks may be higher than clinically apparent, assisting in diagnosis and orienting appropriate treatment
Cardiotoxicity due to	• Identification and quantification of cardiac lesions in patients undergoing these treatments	• Guiding conduct of chemotherapy • Improving understanding of the physiopathology of toxicity due to drugs

MIBG-^123^I: metaiodobenzylguanidine labeled with iodine-123; CAD: coronary artery disease; CRT: cardiac resynchronization therapy; HF: heart failure; HFpEF: heart failure with preserved ejection fraction; HFrEF: heart failure with reduced ejection fraction; ICD: implantable cardioverter defibrillator; LVAD: left ventricular assist device. Adapted from JCS Joint Working Group.^339^

#### 13.3.1. Heart Failure

In this condition, altered cardiac adrenergic innervation is strongly correlated with mortality, and reduced cardiac uptake of MIBG-^123^I (late HMR) confers independent and additional long-term prognostic value to other established markers, such as LVEF and B-natriuretic peptide (BNP).^334-336^ Some studies have demonstrated that abnormalities in cardiac uptake of MIBG-^123^I may be predictive of increased risk of ventricular arrhythmia and sudden cardiac death,^322,337^ with attempts to standardize the procedure for the sake of routine clinical application.^328-330^ Cardiac scintigraphy with MIBG-^123^I has been approved for use in clinical practice in cardiology since 1992 in Japan,^338^ and it is considered a class I indication for evaluation of prognosis and severity of HF, with level of evidence B (Table 31).^338^ Studies with higher numbers of patients have recently been published in Europe and the USA. The AdreView Myocardial Imaging for Risk Evaluation in Heart Failure (ADMIRE-HF)^336^ multicenter, prospective, international study involving the use of MIBG-^123^I in HF independently validated the prognostic value of cardiac scintigraphy with MIBG-^123^I for the evaluation of patients with chronic HF.^337^ They included 961 patients with HF, NYHA HF functional class II-III, and LVEF ≤ 35%, undergoing cardiac scintigraphy with MIBG-^123^I, followed for an average of 17 months. Approximately 25% of patients (n = 237) had cardiac events, with approximately 70% of the first events being progression of chronic HF; 20% potentially lethal arrhythmic events (sustained VT > 30 seconds, heart arrest with cardiac arrest with resuscitation and appropriate ICD firing; and approximately 10% cardiac death; with 22% of patients presenting multiple events. Lower risks of the compound outcome were observed in patients with HMR ≥ 1.60 *versus* HMR < 1.60 (38%; hazard ratio: 0.40; p < 0.001) with a highly significant risk ratio for each individual component of the primary compound outcome evaluated. It is worth highlighting that total mortality over 2 years was around 5 times higher (16.1% *versus* 3.0%) in patients with late HMR < 1.60 compared to those with HMR ≥ 1.60, respectively.^337^

**Table 31:** Recommendations for cardiac scintigraphy with MIBG-^123^I in accordance with the Japanese Circulation Society Guidelines^339^

Indication	Class of recommendation	Level of evidence
Evaluation of severity and prognosis of patients with heart failure (HF)	I	B
Evaluation of the effects of HF treatment	IIa	C
Arrhythmogenic disease	IIb	C

Considering this information, the FDA has approved MIBG-^123^I (*AdreView^R^*), in 2013, for evaluation of cardiac sympathetic innervation in patients with New York Heart Association (NYHA) *HF class II-III* and LVEF < 35%.^326^

In addition to prognostic evaluation of HF,^333-339^ other applications which stand out include the following: evaluation of therapeutic response to medication;^340,341^ indication and evaluation of response to cardiac resynchronization therapy (CRT);^342^ indication for implant and explant of mechanical left ventricular assist device,^343,344^ and implantable cardioverter defibrillator (ICD); and evaluation of reinnervation following cardiac transplant.^345^

Late HMR of MIBG-^123^I in patients with severe chronic HF, in accordance with traditional classification criteria (LVEF, BNP, functional class), may help reclassify patients into a category of lower risk for events. Patients with late HMR ≥ 1.6 (even with very low LVEF and elevated BNP) have a low probability of severe cardiac events during a period of up to 2 years. This information may lead to changes in treatment.^331^ This marker may, also, help refine indication criteria for high-cost invasive therapies for HF, such as CRT^342,346^ and ICD implant.^347,348^ A Brazilian study carried out by Nishioka et al.^342^ has shown that HMR was the only independent predictor of therapy response. Patients with HMR < 1.36 had a lower chance of benefitting from CRT, suggesting that these patients with rather elevated cardiac sympathetic activity have terminal HF.^342,346^

The autonomic nervous system plays an important role in *cardiac* *arrhythmias*. Scintigraphy with MIBG-^123^I has the potential to select patients for ICD implant more accurately, in addition to identifying those at higher risks of sudden cardiac death, who would not be selected in accordance with current guidelines. Arora et al.,^347^ in a pilot study of 17 patients with advanced chronic HF and ICD, divided patients into groups according to the presence or absence of previous ICD firing. In cases with late HMR of MIBG-^123^I < 1.54, they observed a higher frequency of ICD firing and a positive predictive value of 71%, at the same time that increased HMR was observed to have a NPV of 83%.

The etiology of HF is frequently classified as ischemic (I) and non-ischemic (NI). Although the physiopathology and the initial lesion are different, investigation studies have suggested that, as the disease progresses, autonomic cardiac abnormalities are characteristic and common, regardless of etiology. Cardiac scintigraphy with MIBG-^123^I thus continues to be a strong prognostic marker. Wakabayashi et al.^349^ showed that, for both groups, late HMR was the strongest independent predictive factor for sudden cardiac death, although the cutoff points for HMR index values were different, namely 1.50 for ischemic cardiomyopathy and 2.02 for non-ischemic cardiomyopathy. For patients with LVEF < 40% and late HMR lower than the identified cutoff values, the rate of cardiac death was higher in the ischemic group (18.2% annually) than in the non-ischemic group (11.9% annually).

#### 13.3.2. Ventricular Arrhythmia

Sudden cardiac death continues to be one of the leading causes of death worldwide. Scarring and/or non-revascularized/ischemic myocardium provide important substrates for the occurrence of potentially lethal ventricular arrhythmias.^350^ Furthermore, the presence of clinical HF increases the risk of ventricular arrhythmia. The sympathetic nervous system is an important trigger of major arrhythmic events by means of global cardiac adrenergic hyperactivity and heterogeneity of regional myocardial sympathetic activity.^322^ Evaluation of the autonomic nervous system via myocardial scintigraphy (MS) with MIBG-^123^I may be useful in diverse clinical situations. The presence of denervated yet viable myocardium and the magnitude of denervation are potential markers of an individual’s susceptibility to triggering of severe arrhythmias. Several studies have demonstrated the ability of scintigraphy with MIBG-^123^I to identify patients at higher risks of developing spontaneous ventricular tachyarrhythmia, appropriate ICD shock,^351,352^ and sudden cardiac death.^353-356^

When analyzing the possibilities of scintigraphy imaging results, the finding of a *mismatch* between perfusion and myocardial innervation characterizes a scenario of higher risk for ventricular arrhythmia.^350,351^ The denervated regions respond to sympathetic stimuli differently than normal myocardium. This electrophysiological heterogeneity may serve as a substrate for VT and ventricular fibrillation (VF). In the same manner, tomography images (SPECT) of cardiac scintigraphy with MIBG-^123^I are useful for recognizing increased arrhythmogenicity. A prospective study of 50 patients with antecedents of myocardial infarction who underwent SPECT imaging with MIBG-^123^I and perfusion SPECT perfusion with Tetrofosmin-^99m^Tc showed, via multivariate analysis, that a late MIBG-^123^I SPECT defect score of ≥ 37 was the only parameter capable of differentiating the group of patients who presented VT induced by electrophysiological testing, with a sensitivity of 77% and a specificity of 75%.^357^ There were no significant differences in late HMR and the mismatch scores obtained by subtraction of perfusion and sympathetic innervation between the groups with positive and negative induced VT.^356^

Moreover, the Prediction of Arrhythmic Events with Positron Emission Tomography (PAREPET) study evaluated quantification of denervated myocardium in 204 patients with ischemic heart disease (LVEF ≤ 35%), by means of PET imaging with ^11^C-meta-hydroxyephedrine (HED-^11^C), labeled with carbon-11. Perfusion and myocardial viability have also been characterized with ^13^N-ammonia and ^18^F-fluordeoxyglucose, respectively. The primary study objective was to observe the occurrence of sudden cardiac death, defined as arrhythmic death or ICD firing due to VF or VT > 240 beats/minute. After 4.1 years of follow-up, sudden cardiac death of 16.2% was registered. The quantification of infarction volume and LVEF were not factors predictive of sudden cardiac death. However, patients with higher volumes of *denervated* myocardium (33 ± 10% *versus* 26 ± 11% of the LV; p = 0.001) showed sudden arrhythmic death more frequently. The authors of this study concluded that, in ischemic cardiomyopathy, sympathetic denervation evaluated by HED-^11^C PET predicts sudden arrhythmic death regardless of LVEF and infarction volume. This information may improve identification of patients who will most likely benefit from ICD implant.^358^ One of the most peculiar aspects of the natural history of chronic Chagas cardiomyopathy is the occurrence of severe ventricular arrhythmia in individuals with preserved LV global systolic function which may evolve to sudden death during early phases of the disease.^359,360^ In 43 patients with chronic Chagas cardiopathy and LVEF ≥ 35%, the correlation between extent of sympathetic denervation, myocardial fibrosis, and severity of ventricular arrhythmias was investigated. Patients were divided into 3 groups, according to the presence of sustained VT, non-sustained VT, and the absence of VT on 24-hour Holter. Sympathetic denervation was evaluated via SPECT imaging with MIBG-^123^I and myocardial fibrosis via SPECT with ^99m^Tc-sestamibi. The sums of perfusion scores (quantity of fibrosis) were similar in the 3 groups. The summed difference score between MIBG-^123^I and Sestamibi-^99m^Tc, which evaluated the extension of denervated yet viable myocardium, was significantly larger in the group with sustained VT on Holter (score of 20.0 ± 8.0), when compared to the group without VT (2.0 ± 5.0; p < 0.0001) and NSVT (11.0 ± 8.0; p < 0.05). In conclusion, the occurrence of ventricular arrhythmias with different degrees of severity is quantitatively correlated with the extension of cardiac sympathetic denervation, but not with the extension of fibrosis, suggesting that myocardial sympathetic denervation plays a role in the generation of ventricular arrhythmia related to chronic Chagas cardiopathy.^361^

Sympathetic denervation may also occur in patients with stable angina in the absence of infarction. Cardiac scintigraphy with MIBG-^123^I may show inervation defects in these cases, in the absence of perfusion defects. Sympathetic nervous fibers are more susceptible to oxygen privation than cardiomyocytes and the occurrence of myocardial ischemia may thus lead to transient sympathetic denervation.^362^ The fact that recovery of inervation may be a slower process also makes it possible to use myocardial scintigraphy with MIBG-^123^I as a marker of ischemic memory in patients whose chest pain has resolved few hours or days prior.

Regional alterations of cardiac sympathetic activity may also be seen in primary arrhythmic conditions, in the absence of CAD,^363^ in Brugada syndrome,^364^ in hypertrophic cardiomyopathy,^365,366^ and in arrhythmogenic RV dysplasia.^367^ These findings in patients with primary arrhythmias support the potential use of cardiac scintigraphy with MIBG-^123^I for identifying patients at a risk of sudden cardiac death, who may benefit from ICD implant.

#### 13.3.3. Cardiotoxicity Due to Chemotherapy

Over the past decades, there have been great advances in cancer diagnosis and treatment, offering oncology patients reduced mortality, increased survival, and better quality of life. On the other hand, progress in oncological treatment results in higher exposure to the cardiotoxic effects of chemotherapy. Screening for the occurrence of cardiotoxicity (CTX) is highly recommended before, during, and after the completion of chemotherapy. Several methods and diagnostic indexes have been suggested for the detection of CTX and therapeutic strategy planning. Although serial measure of LVEF by conventional echocardiogram is the most utilized strategy for monitoring myocardial damage, it does not appear to be sensitive enough to detect patients with risks of developing significant CTX in early phases of chemotherapy administration.^323^ The potential use of cardiac adrenergic imaging for monitoring the cardiotoxic effects of chemotherapy has been debated.^368-370^ There is evidence that reduced cardiac uptake of MIBG-^123^I precedes ejection fraction deterioration.^371^

A recent study has shown that, following 1 year of treatment with anthracyclines, late HMR was the strongest parameter of scintigraphy with MIBG-^123^I. This index correlates with conventional echocardiography variables and global indexes of radial and longitudinal strain, in addition to doses of galectin-3 in patients with breast cancer treated with anthracyclines. Altered late HMR was a predictor of abnormality in the global radial strain index on ECG.^369^

Cardiac scintigraphy with MIBG-^123^I was performed in 20 women with breast cancer and normal LVEF who had undergone treatment with anthracycline derivatives, associated and not associated with trastuzumab. It was observed that anthracycline use with trastuzumab promoted higher frequency and intensity of cardiac adrenergic hyperactivity.^367^ Carrió et al.^371^ identified abnormal MIBG-^123^I uptake in patients who used anthracyclines, where HMR of MIBG-^123^I decreased as the cumulative dose of this medication increased.^371^

The degree of cardiac uptake of MIBG-^123^I may thus be an early marker of CTX. However, multicenter studies with higher case numbers and standardized exam protocols comparing the evaluation of cardiac sympathetic activity with MIBG-^123^I before and after treatment need to be carried out in order to clarify these findings further.

#### 13.3.4. Cardiac Autonomic Dysfunction in Diabetes Mellitus (DM)

In patients with DM, there is evidence of cardiac denervation in the absence of clinical manifestations.^372^ Diabetic autonomic neuropathy has been implied to be a cause of sudden cardiac death, with or without associated myocardial ischemia. Patients with DM and reduced HMR of MIBG-^123^I have an increased risk for clinical progression of HF.^373^ It is, however, not yet clear whether cardiac adrenergic imaging may improve clinical outcome in patients with diabetes.

#### 13.3.5 Cardiac Transplant

Cardiac scintigraphy with MIBG-^123^I may be useful for evaluation of reinnervation in transplanted hearts. It identifies ventricular sympathetic reinnervation,^345^ which slowly develops from the cardiac base several months following surgery, and it is observed in 40% of patients 1 year following transplant.^374^ Even though the clinical implications and the mechanisms of cardiac reinnervation have yet to be completely made clear, restoration of cardiac sympathetic innervation probably increases physical capacity, due to improved HR and contractile function during exercise in patients with heart transplants.^374^ Evaluation of the process of cardiac reinnervation via scintigraphy with MIBG-^123^I seems to be useful for outpatient treatment of patients with heart transplants for the prescription of appropriate exercises, evaluation of the effect of physical training, and prediction of long-term survival.

#### 13.3.6. Takotsubo Syndrome

Takotsubo syndrome, also known as neurogenic cardiomyopathy, stress-induced cardiomyopathy, or broken heart syndrome,^375^ is characterized by transient left ventricular dysfunction, electrocardiographic alterations similar to those present in AMI, and minimal alterations in cardiac enzymes in the absence of obstructive CAD. It was described in 1991 in Japan^375^ and denominated “Takotsubo” owing to the similarity of the morphological aspect which the LV assumes to a type of trap used to capture octopuses in Japan (round in the bottom and narrow in the upper part). It has recently been recognized as a new entity within the spectrum of acute coronary syndromes.^376-378^ Its real frequency is unknown, but it is estimated that it represents 1% to 2% of cases that present at the emergency room with acute coronary syndrome.^377,379-381^ It generally affects post-menopausal women (95% of cases occur in women between the ages of 60 and 80), and it is rarely (< 3%) seen in women under the age of 50 or in men. In up to 80% of cases, the syndrome is associated with previous events which produced strong physical or emotional stress, such as separations, financial loss, conflicts, loss of a loved one, illness of a loved one, severe disease, surgery, etc. In some cases, however, no preceding physical or emotional stress may be identified.

Several physiopathological mechanisms have been proposed as participants in generating the syndrome, such as occult atherosclerotic disease, multiple coronary spasms, endothelial dysfunction, and microvascular disease. Nevertheless, the most accepted hypothesis is an excess of sympathetic stimulation, with elevated circulating catecholamines causing dynamic obstruction of LV outflow and resulting in short periods of ischemia and ventricular “stunning.”^377,379,380,382-384^ In fact, excessive sympathetic activity with pronounced plasma elevation of catecholamines has been found in almost 75% of patients with Takotsubo syndrome.^379^

The reason why Takotsubo syndrome occurs much more frequently in women after menopause is unknown. Several explanations have been proposed, such as the influence of sexual hormones on the sympathetic neurohumoral axis^379,385^ and coronary vasoreactivity;^379,386^ higher vulnerability of women to myocardial stunning, mediated by the sympathetic system;^379,387^ and alterations in endothelial function following menopause, in response to reduced estrogen levels.^388^

Clinical presentation is characterized by intense, acute chest pain (similar to that of infarction), dyspnea, ischemic ST-segment alterations (ST-segment elevation and/or inversion of T waves and pathological Q waves), mild increase in cardiac enzymes, and segmental systolic dysfunction in the apex and middle third of the LV, with base hyperkinesis, in the absence of obstructive epicardial coronary disease.

The most accepted criteria for diagnosis are currently those proposed by the Mayo Clinic in 2008:^389,390^

- Transient hypokinesis, akinesis, or dyskinesis in LV mid-segments, with or without apical involvement.
- Regional abnormalities that extend beyond epicardial vascular distribution, often with a precipitating factor.
- Absence of CAD or evidence of acute plaque rupture.
- New ECG abnormalities (ST-segment elevation and/or T-wave inversion) or mild elevation in cardiac troponin (disproportional to the degree of LV dysfunction).
- Absence of pheochromocytoma and myocarditis.

**Figure 55:**
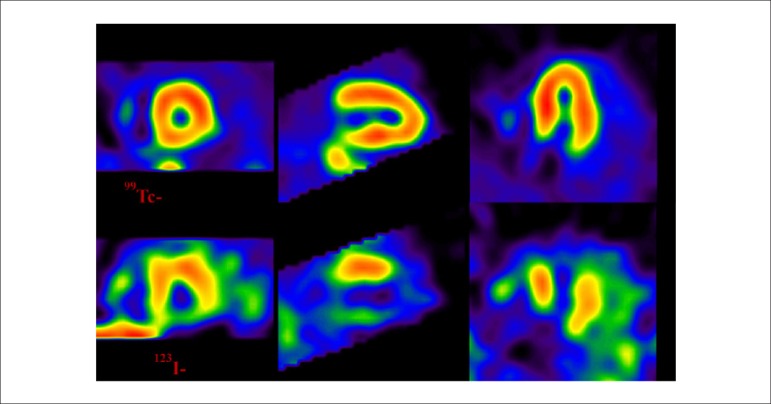
Patient with acute coronary syndrome (ACS). Normal myocardial perfusion scintigraphy (MPS) with Sestamibi-^99m^Tc (top row). Myocardial scintigraphy (MS) with MIBG-^123^I (bottom row) demonstrates uptake defects in apical segments, suggestive of Takotsubo syndrome. Reduced tracer uptake is additionally observed in the inferior wall.

Evaluation via myocardial scintigraphy with MIBG-^123^I shows defects in the uptake of MIBG generally in the apex, with normal myocardial perfusion observed on perfusion scintigraphy with Sestamibi-^99m^Tc (Figure 55). Semiquantitative analysis has also demonstrated reduced HMR and increased washout of MIBG-^123^I. Abnormalities on myocardial scintigraphy with MIBG-^123^I may be detected hours to days following ischemic injury.^391^ For this reason, alterations observed on myocardial scintigraphy with MIBG-^123^I suggest a physiopathological explanation for this syndrome.^392,393^ Prognosis of affected patients is generally favorable. In the vast majority of cases, the LV dysfunction is transient, and complete recovery commonly occurs in around 8 weeks. In rare cases, dysfunction may be accentuated, evolving to cardiogenic shock, ventricular arrhythmia, and death (< 1% intra-hospital mortality).^394^

Personal source.

### 13.4. Final Considerations

The diagnostic and prognostic potential for evaluating the autonomic nervous system with nuclear cardiology is great. A growing amount of evidence has shown that cardiac scintigraphy with MIBG-^123^I may assist in selection of patients for more sophisticated HR treatments, such as CRT, as well as new medical approaches, and ICD implants for primary prevention. It is also a valuable tool for cardiovascular risk stratification (potentially lethal ventricular arrhythmias, progression of HF, and cardiac death). Due to the high sensitivity of autonomic nervous system fibers to ischemic injury and delayed recovery, myocardial scintigraphy with MIBG-^123^I is also useful as an ischemic memory marker or for the recognition of Takotsubo syndrome. Greater clinical experience with this method will, however, be necessary, with the aim of improving positive and negative predictive values, for the sake of greater differentiation of patients with low and high risks, thus contributing to more effective use of medical resources. Japan is the only country where the utility of this imaging technique has been characterized in guidelines. Data related to cost-effectiveness are still limited, and low availability in clinical practice make it difficult to use on a large scale.

## 14. New Applications of Nuclear Cardiology

### 14.1. Introduction

The applications of nuclear cardiology go beyond MPS for ischemic heart disease. Some of the indications which will be discussed are not relatively recent in the literature, but they are still little utilized within our context. In comparison to conventional investigation methods, new non-invasive methods of nuclear medicine in cardiology have the potential to improve early detection of affected myocardium, allowing for quantification of disease activity, orienting therapeutic interventions, and monitoring success of treatment.

### 14.2. Endocarditis

**Figure 56:**
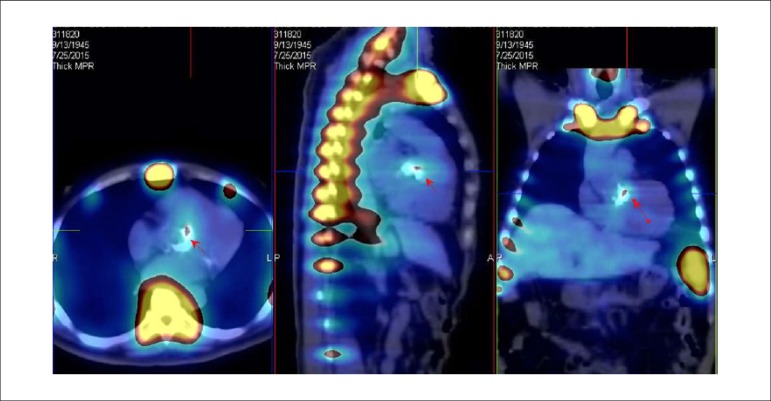
Images from scintigraphy with labeledleukocytes with SPECT/CT demonstrate anomalous accumulation in the area of the percutaneously implanted aortic valve (arrows). Transesophageal echocardiogram was inconclusive, and blood culture was positive for Staphylococcus aureus. The patient was diagnosed with prosthetic valve infective endocarditis.

Early diagnosis of infectious endocarditis (IE) continues to be challenging. The pathology should, essentially, be suspected in the presence of fever of unknown origin, especially in association with laboratory signs of infection, anemia, microscopic hematuria, or manifestations of septic embolism. The modified Duke criteria, which are considered a reference, include clinical, microbiological, and echocardiography findings, resulting in a general sensitivity around 80%.^395^ Some limitations, however, stand out, especially in patients with prosthetic valves (PV) and cardiac implantable electronic devices (CIED),^396^ implying inadequate classification of up to 24% of patients with proven IE.^395^ Advanced imaging techniques for early, sensitive diagnosis of IE are, in fact, valuable tools in clinical practice. The combination of both evaluation of myocardial metabolism of glucose via PET/CT using a glucose analogue labeled with ^18^F, fluorodeoxyglucose (FDG) (FDG-^18^F - PET/CT), and modified Duke criteria resulted in increased sensitivity, without large alterations in specificity.^397^ Although FDG-^18^F - PET/CT is not reliable for evaluation of native valve endocarditis,^398^ it may accurately diagnose endocarditis in valve prostheses and its systemic complications.^399^ In recognition of its utility, FDG-^18^F - PET/CT was included in the European Society of Cardiology Guidelines, in 2015, as a diagnostic criterion (class of recommendation IIb) for IE in patients with valve prostheses.^400^ One option for further improving FDG-^18^F - PET/CT imaging is the incorporation of angio-CT (PET/angio-CT), resulting in sensitivity, specificity, positive predictive value, and negative predictive values of 91%, 91%, 93%, and 88%, respectively.^401^ As a more specific alternative to FDG-^18^F - PET/CT, guidelines on IE include scintigraphy using marked leukocytes with SPECT/CT imaging. SPECT/CT is the combination of nuclear medicine tomography imaging (SPECT) and anatomical imaging via CT, greatly increasing diagnostic accuracy. However, notwithstanding the proven value of this technique for detecting endocarditis^402,403^ (Figure 56), its widespread application is compromised due to limited sensitivity and the difficulty of locating inflammatory foci, but the very high specificity of scintigraphy with marked leukocytes for infection, when using SPECT/CT imaging, may be particularly useful in cases where diagnosis remains uncertain following echocardiography and FDG-^18^F - PET/CT, especially in patients who have undergone cardiac surgery over the past 2 months.^404-407^ As an additional possibility, the simultaneous combination of scintigraphy with marked leukocytes and MPS, acquired to improve localization of infectious points in relation to the valve plane defined by perfusion. Limitations to performing SPECT/CT with marked leukocytes are: the need for a specific structure with laminar flow, the manipulation of blood components, procedure duration, and inferior spatial resolution in relation to PET/CT.^400^

Personal source (courtesy of Dr. Alan Chambi).

Furthermore, new bacteria-specific tracers have become available, such as carbohydrates, which are metabolized exclusively by bacteria or antibodies directed against components of the bacterial cell membrane. For example, the protein component of the pilin structure of *Enterococcus faecalis* is being developed.^408^ This recent study has demonstrated the superior quality of images and another possibility for differentiating between infectious and inflammatory causes of endocarditis.

**Figure 57:**
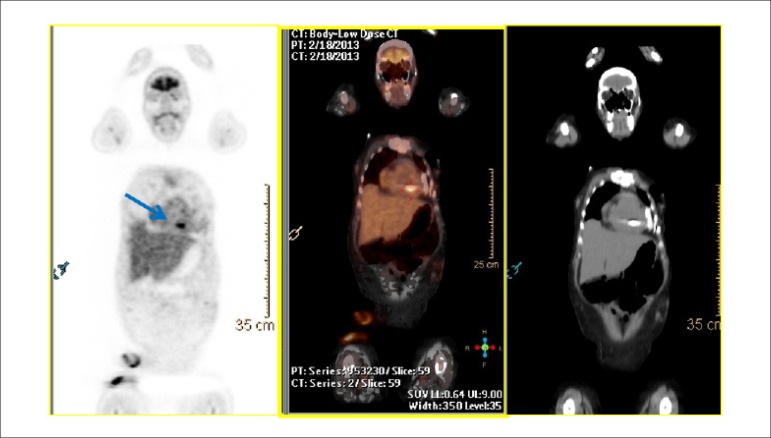
Male patient, age 65, with pacemaker implant 4 months prior (removed due to subcutaneous pocket infection); new implantation with the distal end of the right chambers. He evolved with dyspnea and fever 20 days prior; blood culture was positive for S. aureus. FDG-^18^F - PET/CT study was positive for endocarditis in the implant site; maximum standard uptake value (SUV) = 8.1.

Source: INCOR, FMUSP, SP.

**Figure 58:**
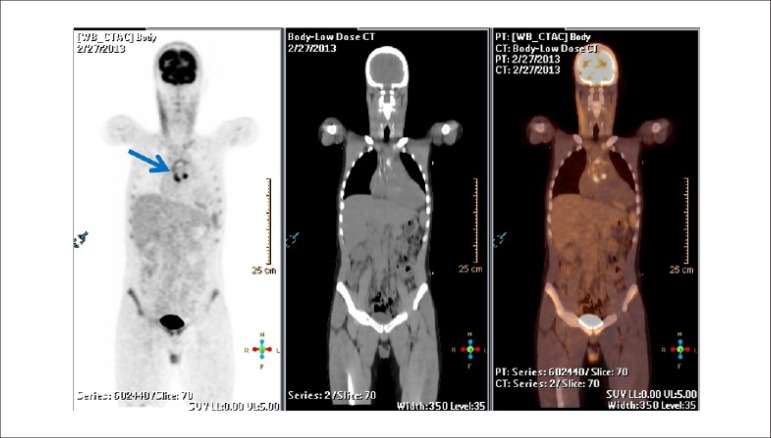
Male patient, age 19, with biological prosthesis in the aorta and mitral annuloplasty for 45 days. He evolved with fever, bacteremia, and blood culture positive for S. Epidermidis. FDG-^18^F - PET/CT study was positive for infection in the aortic prosthesis; maximum standard uptake value (SUV) = 9.7.

Source: INCOR, FMUSP, SP.

CIED have been increasingly used over recent years,^400^ with elevated rates of infection (1% to 3%), and they are associated with 1-year mortality over 10%.^409^ Doppler echocardiography is the first line imaging method for evaluation of suspected CIED infection, but its use is limited for investigating infection in extra-cardiac leads and device pockets. Both FDG-^18^F - PET/CT and SPECT/CT scintigraphy with marked leukocytes have demonstrated additional value for diagnosis of infections related to CIED or pacemaker. FDG-^18^F - PET/CT has been shown to be especially useful for diagnosing device pocket infections, but it is less reliable for diagnosing infections in the metallic device.^410,411^ The presence of a focal hotspot is considered the best criterion for infection,^412^ (Figures 57 and 58). It is worth noting that exam accuracy depends on patient preparation and post-implant interval, which is the case with applications involving FDG-^18^F. Mild FDG-^18^F uptake has been reported to be nonspecific in patients with CIED or pacemaker with no suspicion of acute-phase infection (≤ 2 months) following cardiac surgery.^410^ Moreover, attenuation correction artifacts due to metallic implants should be avoided by means of close evaluation of images without attenuation correction.

Both FDG-^18^F - PET/CT and scintigraphy with marked leukocytes via SPECT/CT seem to be beneficial in diagnosing infections related to ventricular assist devices (VAD).^413,414^ FDG-^18^F - PET/CT is especially sensitive to infection in these devices. In a small retrospective study, sensitivity to VAD infection was 100%, and specificity was 80%. Furthermore, in 85% of cases, PET imaging had an impact on clinical management of patients.^415^

The role of FDG-^18^F - PET/CT in investigating extracardiac complications of infection was also studied. In a retrospective analysis of patients with suspected CIED infection, the performance of full body PET also identified septic embolism or infection disseminated into other sites in 28% of cases.^416^ These results were confirmed in a prospective study on known device endocarditis.^417^ In this cohort, FDG-^18^F - PET/CT found septic embolism in 10 patients (29%), including 7 cases of spondylodiscitis, 4 of which were not clinically visible and which resulted in significant modifications to therapy.

Guided myocardial biopsy may be another application of FDG-^18^F - PET/CT, as shown in other diseases.^418^ Furthermore, MR and PET/CT seem to be complementary in nature.^419^ Investigation of the incremental value of PET/MR, a new integrated imaging modality, may have great potential for diagnosing endocarditis.

### 14.3. Myocarditis

The most common causes of myocarditis are viral infections. Other causes include other types of infections, autoimmune disorders, or drug interactions. Clinical manifestations of myocarditis are highly variable, ranging from subclinical disease to sudden death. This spectrum also reflects the extent to which this histological disease’s severity, etiology, and stage of clinical presentation may vary. Inflammation of the myocardium may be focal or diffused, involving any of the cardiac chambers. Endomyocardial biopsy is currently the gold standard for diagnosis, but it has a low sensitivity (20-30%) and significant associated risk.^420^ MR is considered the imaging method of reference for non-invasive diagnosis of myocarditis, given that it allows for detection of several characteristics such as inflammatory hyperemia and edema, necrosis, and myocardial scarring, alterations in ventricular size and geometry, regional and global abnormalities in the movement of walls, and identification of pericardial effusion.^421^ MR criteria for diagnosis of myocarditis have been summarized in what are known as the Lake Louise Criteria.^422^ MR, however, has limitations that are particularly evident in chronic myocarditis, with low diagnostic precision (50% accuracy).^423^

Using FDG-^18^F - PET/CT, following adequate patient preparation with a carbohydrate-free diet, it is possible to visualize acute inflammation suggestive of active myocarditis. PET imaging may help distinguish active and chronic forms of the disease, following established working protocols.^424,425^ In a prospective study of 65 patients with suspected myocarditis, FDG-^18^F - PET was in agreement with MR findings.^426^ MR and FDG-^18^F - PET/CT seem to be complementary in nature.^419^ For this reason, cardiac PET/MR has potential as a diagnostic tool for myocarditis and a new field of research.^427-429^

### 14.4. Pericarditis

There are multiple causes of acute or chronic pericardial inflammation, including infections (viral, bacterial, or fungal), myocardial infarction, trauma, malign diseases (primary pericardial neoplasm, pericardial metastases, or paraneoplastic syndrome), autoimmune or inflammatory diseases, and metabolic disorders (uremia). Pericarditis may also be iatrogenic, as a collateral effect of medication. Radiotherapy or idiopathic causes are other possible origins. Although its etiology is variable, the pericardium’s response to different causes is not specific. Inflammation of pericardial layers and increased production of pericardial fluids are the most common, and they often manifest as chest pain. In the same manner, Doppler echocardiography stands out as a priority for diagnosis and therapeutic follow-up of pericarditis, externalizing findings such as pericardial effusion and thickness. Generally, CT and MR also allow for evaluation of pericardial effusion and thickness, allowing for better differentiation of pericardium and pericardial fluid.^430^

The use of FDG-^18^F - PET/CT in pericarditis is generally complementary, and it demonstrates the ability to detect inflammatory tissue, even in the absence of obvious anatomical changes.^431,432^ Non-infectious inflammatory pericarditis shows mild to moderate FDG-^18^F - PET uptake in the pericardium, with diffuse or focal uptake pattern. The literature is still scarce on the utility of FDG-^18^F - PET/CT for differential diagnosis of the underlying causes of this pathology. Some studies relate the possibility of differentiating infectious/inflammatory pericardial disease and neoplastic/metastatic disease, given that malignity, generally, presents intense metabolic activity.^433^ Constrictive or effusive pericarditis, an uncommon complication of chemotherapy, may also present pericardial uptake of FDG-^18^F, with mild intensity and wide distribution.^431^ Only a few case reports are available in the literature, and larger studies are still necessary to determine the accuracy of FDG-^18^F - PET/CT for pericarditis.

### 14.5. Cardiac Sarcoidosis

**Figure 59:**
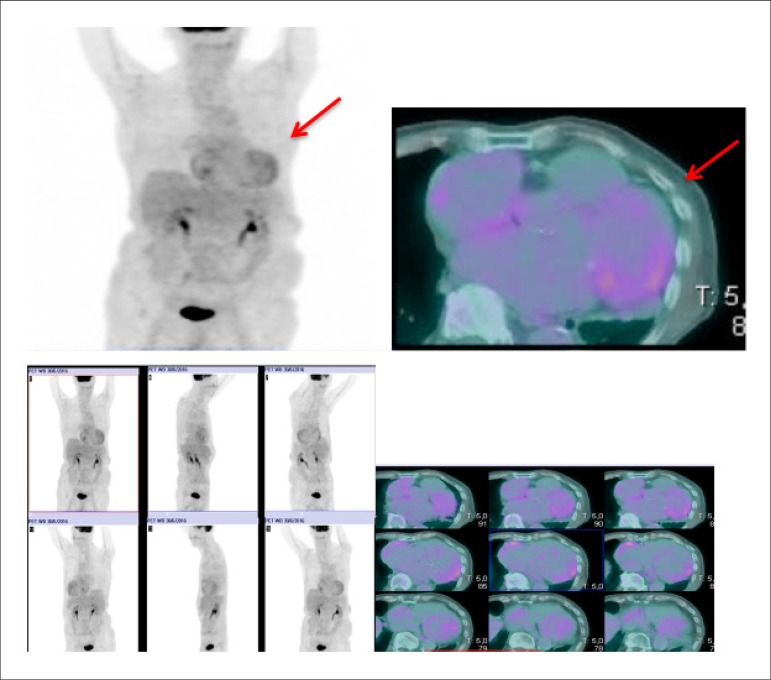
FDG-^18^F - PET/CT with suppression protocol for myocardial glucose uptake (diet), maximum-intensity-projection imaging (left) and coregistration with CT (right). Patient, age 88, with heart failure, reduced ejection fraction, and ventricular tachycardia. Endomyocardial biopsy was compatible with sarcoidosis. Images demonstrated abnormal tracer uptake in the right and left ventricles (arrows). Following immunosuppression, the patient showed clinical improvement and disappearance of abnormalities.

Sarcoidosis is a granulomatous disease whose etiology is unknown. It most commonly affects the lymphatic ganglia and the lungs, but it may involve any system of organs.^434^ The heart is frequently affected,^435,436^ and this represents one of the main causes of death due to this pathology in Japan and the USA.^437^ Due to its multifocal aspect and the irregular manner in which sarcoidosis affects the myocardium, the sensitivity of endomyocardial biopsy is extremely low (20% to 30%).^438^ In comparison with MR, the advantages of FDG-^18^F - PET/CT include the value of functional metabolic information, the detection of active inflammation, the potential for identifying cardiac and extracardiac involvement (Figure 59) of sarcoidosis, and the possibility of performing imaging in patients with CIED or renal insufficiency. In order to evaluate extracardiac involvement, it is important to perform full-body imaging.

Personal source (courtesy of Dr. Evandro T. Mesquita).

Sarcoidosis normally manifests as an irregular focal uptake pattern. FDG-^18^F - PET/CT has demonstrated that it detects active cardiac and extracardiac forms reliably, with sensitivity between 81% and 89% and specificity between 78% and 82%, respectively.^439,440^ It is necessary to pay attention to the patient preparation required for image acquisition in these cases. It is essential for the patient’s diet to be low in carbohydrates and rich in fat the day before the exam and for the patient to be in fasting conditions in order to guarantee that there is no physiological uptake in the myocardium.

**Figure 60:**
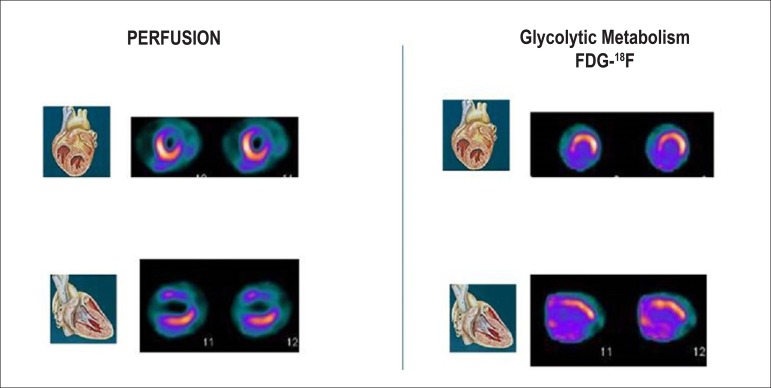
Patient with cardiac sarcoidosis. Myocardial perfusion scintigraphy (MPS) with MIBI-^99m^Tc (left), showing evidence of accentuated persistent hypoperfusion in the anterior and anterolateral walls of the left ventricle; FDG-^18^F - PET imaging (right) for metabolic study shows that regions with apparent fibrosis were in fact inflammation due to sarcoidosis.

Source: INCOR, FMUSP, SP.

FDG-^18^F - PET/CT may often be combined with MPS synchronized with ECG (Figure 60), with the objective of ruling out CAD or even identifying resting perfusion defects suggestive of inflammation-induced tissue damage.^441,442^

In addition to this, FDG-^18^F - PET/CT in combination with perfusion imaging has shown evidence of prognostic capability in patients with sarcoidosis,^443^ orienting myocardial biopsy,^418^ and demonstrating valor for predicting response and monitoring therapy.^444^

### 14.6. Cardiac Amyloidosis

The CA is a rare form of cardiomyopathy. Frequently subdiagnosed, it is characterized by extracellular deposition of fibrils, composed of varied serum protein subunits, which have low molecular weight. Although more than 30 different amyloid proteins have been described, the 2 that most frequently infiltrate the heart are: light chain immunoglobulin (AL) and transthyretin (TTR). The AL and TTR forms possess different clinical courses, prognoses, and distinct forms of treatment. In the AL form, fibrils are composed of light-chain immunoglobulins and produced by a population of plasma cell clones located in the bone marrow. In the TTR form, deposits are made up of anomalous monomers or dimers of the hepatic tetrameric protein whose origin may be related to genetic mutations of familial origin (mutated TTR or [mTTR]) or the wild type, formerly known as the senile type (sTTR). More than 100 known mutations are related to mTTR and to autosomal dominant inheritance, which may affect individuals in any age group, especially middle-aged men. The most common manifestation of CA is HF with preserved ejection fraction. In its final stage, it is present as restrictive cardiomyopathy, implying very poor prognosis. Definitive diagnosis requires amyloid deposits on endomyocardial biopsy or, in patients with suggestive cardiac findings, amyloid deposits on histological exams of other tissues (e.g., abdominal fat, rectum, or kidneys).^445^

Echocardiography is the initial non-invasive exam of choice for diagnosing CA, but its specificity is limited.^446^However, complementary sequence with MR, which has satisfactory sensitivity, may suggest a pattern of cardiomyopathy due to amyloid deposition, except in patients with moderate to severe kidney disease.

It has been reported that scintigraphy with intravenous administration of bisphosphonate radiotracers labeled with 99m-technetium (Pyrophosphate-^99m^Tc is the most used in Brazil) localizes cardiac amyloid deposition. It is considered sensitive and highly specific for TTR CA, identifying the disease early at onset.^447,448^ One hypothesis for the binding of these bone markers to amyloid fibers is related to the higher quantity of calcium present in TTR protein, in relation to AL. In a recent multicenter study which included 1,217 patients with suspected CA, the combination of moderate to accentuated increase in myocardial uptake of the radiotracer and the absence of specific monoclonal protein in blood serum or urine, had specificity and positive predictive value of 100% for the TTR form of CA. However, scintigraphy with bisphosphonate radiotracers does not reliably detect other types of CA, and it cannot be used quantitatively for therapeutic monitoring.^418^

**Figure 61:**
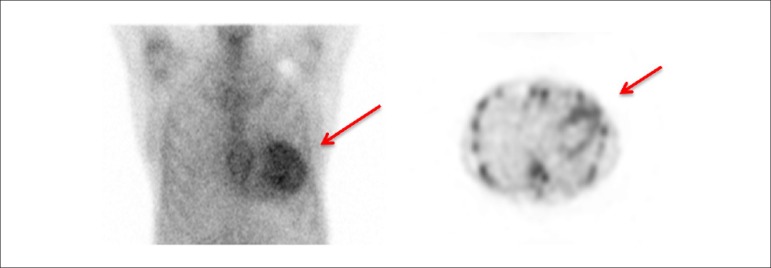
Scintigraphy with Pyrophosphate-^99m^Tc (left: planar imaging of the anterior thorax; right: axial cross section of the tomography image) demonstrated severe radiopharmaceutical uptake in the left ventricle (arrows) in a patient with confirmed transthyretin cardiac amyloidosis.

**Figure 62:**
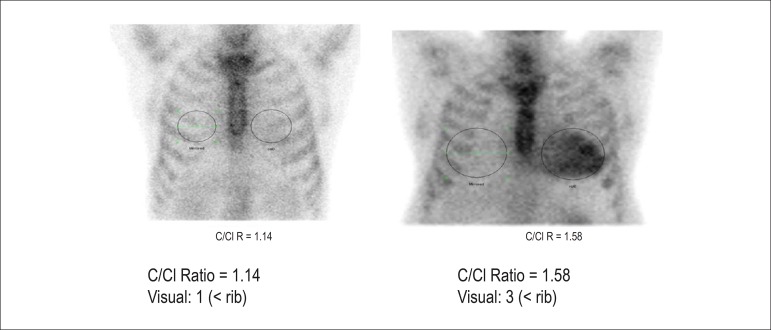
Quantification of uptake in planar imaging of anterior thorax scintigraphy with Pyrophosphate-^99m^Tc. The image on the left represents a negative study, without uptake in the cardiac area. The image on the right represents a study positive for amyloidosis, with accentuated diffuse uptake in the left ventricle. For the purpose of quantification, circular regions of interest (ROI) have been drawn in both hemithoraxes, and the uptake ratio of the radiopharmaceutical between the ROI in the cardiac area (C) and the ROI in the contralateral (Cl) hemithorax. Results over 1.5 suggest TTR amyloidosis. The visual uptake score was equal to 1 in the image on the left and 3 in the image on the right.

Source: INCOR, FMUSP, SP.

The intensity of the concentration of Pyrophosphate-^99m^Tc in the cardiac area is correlated to the amyloid subtype. Degree of concentration is compared to bone uptake in the ribcage, considering the following: degree 3, greater uptake than the ribs; degree 2, equal to the intensity of concentration in the ribs; degree 1, lower concentration than in the ribs; and degree zero, no significant cardiac tracer concentration. Severely increased concentration (degrees 2 and 3) (Figure 61) is strongly associated with TTR CA, to the extent that some authors suggest dispensing cardiac biopsy in these situations. Less intense increased concentration (degree 1) and absence of increased concentration suggest the AL form, when there is clinical suspicion. Semiquantitative analysis of radiotracer uptake should also be performed (Figure 62).^449^

Personal source (courtesy of Dr. Rafael Willain Lopes).

A potential bone radiotracer for PET amyloid imaging is sodium fluoride labeled with ^18^F, or Sodium fluoride-^18^F. It has been described in single case series, whereas another study identified no increase in uptake of this tracer with TTR CA,^450-452^ indicating the need for further studies to investigate the potential value of PET/CT with Sodium fluoride-^18^F for CA.

A small amount of available data has demonstrated the limited application of FDG-^18^F - PET/CT for evaluation of CA.^453,454^ Up to the present moment, the most promising alternatives include other specific amyloid markers, such as the Pittsburgh B compound labeled with carbon-11 (PIB-^11^C),^455,456^ as well as other compounds labeled with ^18^F, such as Florbetapir^457,458^ and Florbetaben.^459^ All studies have reported promising results for diagnosis of CA, given that PIB-^11^C presents uptake in AC. It has additionally been demonstrated that PIB-^11^C has lower uptake in patients who have been treated with chemotherapy, in comparison with patients still undergoing treatment. Thus, PIB-^11^C - PET has the potential to be used for therapeutic monitoring of patients with light-chain CA as a marker of disease activity.^460^

### 14.7. Final Considerations

New applications of nuclear medicine in cardiology (Table 32) represent an important area which is little explored in our context. They have the capability to detect functional alterations in these pathologies, indicating whether a disease is or active or not and assisting in therapeutic monitoring. Cardiologists’ knowledge of these applications will be essential to their proper use and to the dissemination of these diagnostic methods.

**Table 32:** Exam types and main scintigraphy findings of new applications of nuclear cardiology

Pathology	Exam	Main findings
TTR CA (hereditary or wild)	Scintigraphy with ^99m^Tc-pyrophosphate	Shows moderate to severe radiotracer uptake; high accuracy for detection of TTR form (PPV 100%); allows for early diagnosis; reflects extent of deposit; prognostic marker
Light chain CA	Scintigraphy with ^99m^Tc-pyrophosphate	Absence of uptake or slight cardiac uptake
Sarcoidosis	FDG-^18^F - PET/CT	Cardiac hypermetabolism demonstrating active in-flammation
	Myocardial perfusion scintigraphy	Persistent myocardial hy-poperfusion suggestive of tissue damage due to in-flammation
Endocarditis	FDG-^18^F - PET/CT	Hypermetabolism in areas of infection
	Scintigraphy with marked leukocytes	High uptake in areas of infection

CA: cardiac amyloidosis; PPV: positive predictive value; TTR: transthyretin amyloidosis.

## Figures and Tables

**Table 21:** Percent probability of obstructive coronary artery disease, considering the presence of chest pain, sex, and age. Adapted from Diamond GA, Forrester JS and the Brazilian Cardiology Society's Third Guidelines on Exercise Testing^117,118^

Age	Non-anginal chest pain		Atypical angina		Typical angina	
	Men	Women	Men	Women	Women	Men
30-39	4	2	34	12	76	26
40-49	13	3	51	22	87	55
50-59	20	7	65	31	93	73
60-69	27	14	72	51	94	86

## Footnotes

* To access the Addendum bibliography, go to: https://sbc-portal.s3.sa-east-1.amazonaws.com/diretrizes/Publicacoes/2020/Bibliografia-adendo/Bibliografia_ADENDO_INGLES_24-01-2020.pdf
